# Differential impacts of juvenile hormone, soldier head extract and alternate caste phenotypes on host and symbiont transcriptome composition in the gut of the termite *Reticulitermes flavipes*

**DOI:** 10.1186/1471-2164-14-491

**Published:** 2013-07-19

**Authors:** Ruchira Sen, Rhitoban Raychoudhury, Yunpeng Cai, Yijun Sun, Verena-Ulrike Lietze, Drion G Boucias, Michael E Scharf

**Affiliations:** 1 Department of Entomology, Purdue University, West Lafayette, IN, USA; 2 Interdisciplinary Center for Biotechnology Research, University of Florida, Gainesville, FL, USA; 3 Entomology and Nematology Department, University of Florida, Gainesville, FL, USA; 4 Current Address: Research Center for Biomedical Information Technology, Shenzhen Institutes of Advance Technology, Chinese Academy of Sciences, Shenzhen, China; 5 Current Address: Department of Microbiology and Immunology & New York State Center of Excellence in Bioinformatics and Life Sciences, The State University of New York at Buffalo, Buffalo, NY 14203, USA

**Keywords:** Metagenomics, Soldier head extract, Microarray, Caste differentiation, Live soldier, Live reproductive

## Abstract

**Background:**

Termites are highly eusocial insects and show a division of labor whereby morphologically distinct individuals specialize in distinct tasks. In the lower termite *Reticulitermes flavipes* (Rhinotermitidae), non-reproducing individuals form the worker and soldier castes, which specialize in helping (*e.g.*, brood care, cleaning, foraging) and defense behaviors, respectively. Workers are totipotent juveniles that can either undergo status quo molts or develop into soldiers or neotenic reproductives. This caste differentiation can be regulated by juvenile hormone (JH) and primer pheromones contained in soldier head extracts (SHE). Here we offered worker termites a cellulose diet treated with JH or SHE for 24-hr, or held them with live soldiers (LS) or live neotenic reproductives (LR). We then determined gene expression profiles of the host termite gut and protozoan symbionts concurrently using custom cDNA oligo-microarrays containing 10,990 individual ESTs.

**Results:**

JH was the most influential treatment (501 total ESTs affected), followed by LS (24 ESTs), LR (12 ESTs) and SHE treatments (6 ESTs). The majority of JH up- and downregulated ESTs were of host and symbiont origin, respectively; in contrast, SHE, LR and LS treatments had more uniform impacts on host and symbiont gene expression. Repeat “follow-up” bioassays investigating combined JH + SHE impacts in relation to individual JH and SHE treatments on a subset of array-positive genes revealed (i) JH and SHE treatments had opposite impacts on gene expression and (ii) JH + SHE impacts on gene expression were generally intermediate between JH and SHE.

**Conclusions:**

Our results show that JH impacts hundreds of termite and symbiont genes within 24-hr, strongly suggesting a role for the termite gut in JH-dependent caste determination. Additionally, differential impacts of SHE and LS treatments were observed that are in strong agreement with previous studies that specifically investigated soldier caste regulation. However, it is likely that gene expression outside the gut may be of equal or greater importance than gut gene expression.

## Background

The successful maintenance of social insect colonies relies on the efficiency of workers. Functions like foraging, cleaning, brood care, colony defense, etc. are carried out by individuals, which are often differentiated into worker sub-castes that each performs specific duties. Such differentiation is either morphological, where physical features determine the tasks (polyphenism), or age-based, where workers carry out different functions at different ages (polyethism) [1]. In both types of caste differentiation, juvenile hormone (JH) is known to play a significant role in most social insects [2]. Experimental application of JH to workers of different social insects has been shown to induce two major types of changes; such as (i) stimulating young workers to carry out functions that are usually performed by older individuals [3] or (ii) inducing workers to go through physical changes and differentiate from one phenotype to another [4].

Termite colonies contain one or more pairs of reproductives and a large number of non-reproductives that are morphologically differentiated into workers, nymphs and soldiers. Workers and nymphs carry out foraging, brood care and cleaning while soldiers, with bigger and stronger mandibles, are dedicated to colony defense. As hemimetabolous insects, termites go through several juvenile instars before reaching adulthood; in each of these juvenile instars JH is presumed to perform its stereotypical “status quo” function [2]. In lower termites, including *Reticulitermes flavipes*, helper castes are juvenile stages composed of both workers, which are eyeless and wingless, and nymphs, which are immature imagoes [5]. Nymphs and workers diverge from undifferentiated “larvae” after the second instar. Workers have three alternate developmental trajectories that include (i) status quo molts into workers, (ii) molts into presoldiers (followed by terminal soldier differentiation), or (iii) molts into apterous neotenic reproductives. Nymphs, conversely, have two alternate developmental trajectories that include molts into (i) brachypterous neotenic reproductives or (ii) adult imagoes that eventually become primary reproductives that start incipient colonies. Aside from two caste-regulatory genes and two soldier-derived primer pheromones linked to presoldier caste regulation [5-7], relatively little is known about the molecular mechanisms of caste differentiation in termites and the factors that initiate this process.

Different juvenile hormone analogues (JHAs) have been tested for their effect on caste differentiation in many species of termites through various bioassay methods [8]. Most of these experiments demonstrated increased production of presoldiers, intercastes and pseudoimagoes and increased or decreased production of neotenic reproductives. The application of JH also resulted in defaunation of protozoan symbionts and bacterial endosymbionts, inhibition of ecdysis and feeding, atrophication of the prothoracic gland, and apparent toxicity [8]. It can thus be hypothesized that JH and host-symbiont interactions within the gut may impact caste differentiation processes.

In *R. flavipes* and other species of the genus *Reticulitermes,* JH and JHAs predominantly induce presoldier differentiation [7,9-12]. Moreover, Elliott and Stay [13] have suggested that, in *R. flavipes*, workers that are destined to become presoldiers have a 2.5-fold higher JH titer compared to workers destined to become neotenic reproductives. Park and Raina [14,15] also reported an increased titer of JH in presoldiers and new soldiers of a closely related species, *Coptotermes formosanus*. Soldiers are known to have inhibitory effects on presoldier differentiation mediated by soldier-derived primer pheromones in lower termites [16-18]. Two primer pheromone candidates, ɣ-cadinene (CAD) and its aldehyde ɣ-cadinenal (ALD) have been identified in *R. flavipes* soldier head extract (SHE), which, when applied in combination with JH, increases soldier caste differentiation [7,12]. However, SHE alone does not impact caste differentiation, survivorship, or any other aspect of worker biology [7,12,18]. Also, two fat body-expressed hexamerin-encoding genes (*Hex-1* and *Hex-2*) play a key role in maintaining a developmental status quo in workers by being JH-inducible, sequestering JH and thereby promoting high worker caste proportions [11,19-22].

In addition to endocrine effects, social effects on gene expression have been investigated to some extent in *R. flavipes.* For example, being held with soldiers increases levels of the primer pheromone ALD by 10× in workers [7] and such workers are less likely to undergo presoldier formation [7,18]. However, with respect to reproductive effects, while some phenotypic impacts have been noted in *R. speratus*[37], in *R. flavipes* the impact on workers of being held with live reproductives is not known*.* Termite biology, however, is also influenced by the numerous symbionts that are harbored in the termite gut. In *R. flavipes*, these symbionts consist of both pro- and eukaryotes and include over 5,000 ribotypes of prokaryotes [23] and 11–12 different protists [24]. The protists, especially, have been shown to be in a nutritional symbiosis with *R. flavipes*[25]. Despite this emerging understanding, no studies have yet focused on the worker termite gut or its resident symbionts as potential molecular determinants of caste differentiation. Since the lower-termite gut environment is centrally important to nutrition, physiology and symbiotic relationships with protists and bacteria, we hypothesized that gene expression in this environment is substantially altered in response to caste-regulatory factors and caste composition. Raychoudhury *et al.*[26] recently tested the effects of different diets on gene expression of both the termite gut and protist symbionts using an oligonucleotide cDNA microarray. Here we use the same protocol to test the effects of hormones (*i.e.*, JH), primer pheromones (SHE) and the presence of live reproductives (LR) and live soldiers (LS) on host and symbiont gene expression in the gut of *R. flavipes* workers. We show that JH has the most pronounced effect while SHE treatment and the presence of live reproductives or soldiers have much lower impacts on gene expression in the gut environment. We further investigated the expression level of a few selected upregulated and downregulated genes (as found by microarray) in termites independently treated with JH, SHE and the JH + SHE combination. Lastly, we report details on a previously un-annotated 50-kDa midgut protein gene that was the most significantly JH-upregulated EST.

## Results

### JH-presoldier induction bioassays

Presoldier induction bioassays were conducted on one Florida colony used for microarray studies (B1) and another from Indiana used in “follow up” qPCR bioassays (WI-9). After a limited 24-hr exposure period identical to that employed in microarray and follow up bioassays, the B1 and WI-9 colonies had average ± std. deviation presoldier formation of 90.0 ± 7.1% and 91.1 ± 11.5% after 25 days (n = 4-5 per colony). No presoldier formation (0%) occurred in acetone controls.

### Numbers of differentially expressed transcripts and their annotation

**Figure 1:**
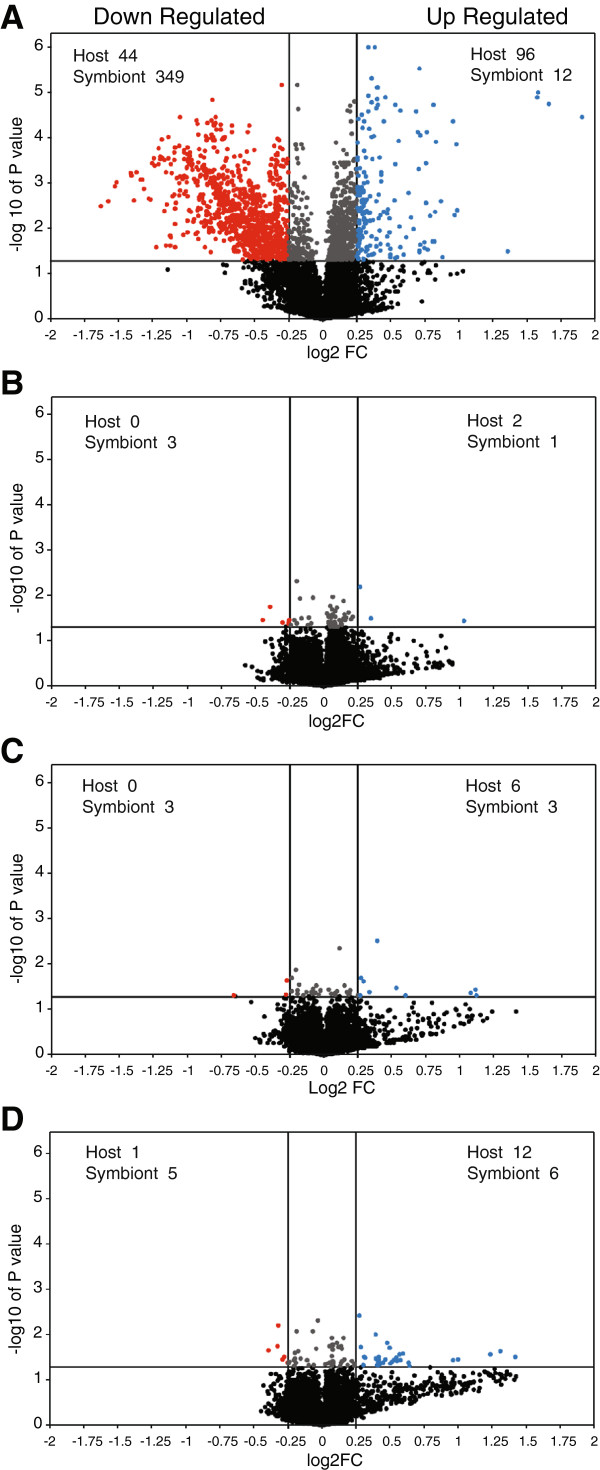
**Volcano plots illustrating microarray results.** Shown are numbers of significantly (*P* <0.05) upregulated (blue; ≥1.19-fold) and downregulated (red; ≤0.84-fold) microarray positions from four treatments that included juvenile hormone **(A)**, soldier head extract **(B)**, live reproductives **(C)** and live soldiers **(D)**. The fold ratios on the x-axis represent the ratio of EST abundance for each of the four treatments relative to untreated controls. The numbers of contigs formed from ESTs of host or symbiont origin are given in each panel.

A total of 10,990 distinct transcripts, represented in 15,208 array positions, were studied in this microarray experiment (see Methods for details). To visualize the array data, we calculated a fold change ratio for every array position based on its normalized average intensity in each treatment (*i.e.*, JH, SHE, LS, LR) divided by its normalized average intensity in the control (acetone) treatment. We selected array positions that had significant fold ratios (*P* <0.05) and mean log_2_ fold change ratios ≤ −0.25 and ≥0.25 (emphasized in the Volcano plots in Figure 1) which corresponded to actual fold changes of <0.84 and >1.19, respectively. Array positions with ≥0.25 mean log_2_ fold change ratio represented ESTs which were upregulated by the various treatments, and array positions with ≤ −0.25 mean log_2_ fold change ratio represented ESTs which were downregulated by the various treatments.

### Effects of juvenile hormone III (JH)

A total of 179 and 981 JH up- and downregulated microarray positions were identified. After contiging the upregulated and downregulated ESTs separately (90% similarity level), we found 96 and 12 unique sequences of host and symbiont origin, respectively, in the upregulated set; in the downregulated set, we found 44 and 349 unique sequences of host and symbiont origin, respectively, along with 6 sequences of mixed origin (Table 1, Figure 1). The term “EST” is used hereafter to refer to both contigs and non-contiguous orphan EST “singletons”. Significantly more upregulated ESTs were from the host termite; whereas, significantly more downregulated ESTs were from protist symbionts (G test for independence, G = 242.7, *P* <0.001). Out of 108 JH upregulated ESTs, 74 could be annotated by BLASTx against the nr database through BLAST2GO, and 332 out of 399 downregulated ESTs could be annotated (Tables 2 and 3, Additional file 1: Table S1A, S1B).

**Table 1:** Number of upregulated and downregulated ESTs in different treatments compared to acetone controls

**Treatment**	**Upregulated**		**Downregulated**		**Total unique ESTs and contigs**
	**Host**	**Symbiont**	**Host**	**Symbiont**	
JH	158 (96)	21 (12)	82 (44)	899 (349)	501
SHE	2	1	0	5 (3)	6
LR	7 (6)	3	0	3	12
LS	22 (12)	15 (6)	1	5	24

The numbers in parentheses are numbers of unique sequences that were found after contiging at 90% sequence similarity.

**Table 2:** KEGG pathways upregulated by JH treatment

	**Pathway**	**Enzyme**	**Ezyme ID**	**Accession#**	**Origin**
1	Amino sugar and nucleotide sugar metabolism	GDP-L-fucose synthase	EC:1.1.1.271	FL638281	Host
2	Arginine and proline metabolism	aldehyde dehydrogenase (NAD+)	EC:1.2.1.3	FL638461	Host
3	Ascorbate and aldarate metabolism	aldehyde dehydrogenase (NAD+)	EC:1.2.1.3	FL638461	Host
4	Beta-Alanine metabolism	aldehyde dehydrogenase (NAD+)	EC:1.2.1.3	FL638461	Host
5	Chloroalkane and chloroalkene degradation	aldehyde dehydrogenase (NAD+)	EC:1.2.1.3	FL638461	Host
6	Fatty acid metabolism	aldehyde dehydrogenase (NAD+)	EC:1.2.1.3	FL638461	Host
	Fructose and mannose metabolism	GDP-L-fucose synthase	EC:1.1.1.271	FL638281	Host
7	Glycerolipid metabolism	aldehyde dehydrogenase (NAD+)	EC:1.2.1.3	FL638461	Host
8	Glycolysis Gluconeogenesis	aldehyde dehydrogenase (NAD+)	EC:1.2.1.3	FL638461	Host
		glyceraldehyde-3-phosphate dehydrogenase (phosphorylating)	EC:1.2.1.12	FL642856	Symbiont
9	Histidine metabolism	aldehyde dehydrogenase (NAD+)	EC:1.2.1.3	FL638461	Host
10	Limonene and pinene degradation	aldehyde dehydrogenase (NAD+)	EC:1.2.1.3	FL638461	Host
11	Lysine degradation	aldehyde dehydrogenase (NAD+)	EC:1.2.1.3	FL638461	Host
12	Methane metabolism	peroxidase	EC:1.11.1.7	FL637172	Host
13	Pentose and glucuronate interconversions	aldehyde dehydrogenase (NAD+)	EC:1.2.1.3	FL638461	Host
14	Phenylalanine metabolism	peroxidase	EC:1.11.1.7	FL637172	Host
15	Phenylpropanoid biosynthesis	peroxidase	EC:1.11.1.7	FL637172	Host
16	Porphyrin and chlorophyll metabolism	ferroxidase	EC:1.16.3.1	FL636458	Host
17	Propanoate metabolism	aldehyde dehydrogenase (NAD+)	EC:1.2.1.3	FL638461	Host
18	Purine metabolism	guanylate cyclase	EC:4.6.1.2	FL639621, FL639829	Host
19	Pyruvate metabolism	aldehyde dehydrogenase (NAD+)	EC:1.2.1.3	FL638461	Host
20	Retinol metabolism	retinal dehydrogenase	EC:1.2.1.36	FL638191	Host
21	Tryptophan metabolism	aldehyde dehydrogenase (NAD+)	EC:1.2.1.3	FL638461	Host
22	Tyrosine metabolism	dopamine beta-monooxygenase	EC:1.14.17.1	FL640448	Host
23	Valine, leucine and isoleucine degradation	aldehyde dehydrogenase (NAD+)	EC:1.2.1.3	FL638461	Host

Pathways were obtained from sequences corresponding to upregulated spots from the array. Annotations were done with BLAST2GO. The table also indicates the library of origin (host/symbiont) of the sequences.

**Table 3:** KEGG pathways downregulated by JH treatment

	**Pathway**	**Enzyme**	**Ezyme ID**	**Accession #**	**Origin**
1	Amino sugar and nucleotide sugar metabolism	glucose-6-phosphate isomerase	EC:5.3.1.9	FL643307	Symbiont
2	Beta-Alanine metabolism	dihydropyrimidine dehydrogenase (NADP+)	EC:1.3.1.2	FL641335	Symbiont
3	Butanoate metabolism	hydroxymethylglutaryl-CoA synthase	EC:2.3.3.10	FL641384	Symbiont
4	C5-Branched dibasic acid metabolism	succinate---CoA ligase (ADP-forming)	EC:6.2.1.5	FL641410, FL643051, FL644288, FL645720	Symbiont
5	Carbon fixation in photosynthetic organisms	malate dehydrogenase (oxaloacetate-decarboxylating) (NADP+)	EC:1.1.1.40	FL642239, FL642831, FL645272, FL645325	Symbiont
		triose-phosphate isomerase	EC:5.3.1.1	FL642787	Symbiont
		phosphoglycerate kinase	EC:2.7.2.3	FL643941	Symbiont
		fructose-bisphosphate aldolase	EC:4.1.2.13	FL644124	Symbiont
6	Carbon fixation pathways in prokaryotes	succinate---CoA ligase (ADP-forming)	EC:6.2.1.5	FL641410, FL643051, FL644288, FL645720	Symbiont
		ATP citrate synthase	EC:2.3.3.8	FL641410, FL643051, FL644288, FL645720	Symbiont
7	Citrate cycle (TCA cycle)	succinate---CoA ligase (ADP-forming)	EC:6.2.1.5	FL641410, FL643051, FL644288, FL645720	Symbiont
		ATP citrate synthase	EC:2.3.3.8	FL641410, FL643051, FL644288, FL645720	Symbiont
		succinate---CoA ligase (GDP-forming)	EC:6.2.1.4	FL641410, FL643051, FL644288, FL645720	Symbiont
		succinate---CoA ligase (GDP-forming)	EC:6.2.1.4	FL641015, FL643186	Symbiont
		succinate---CoA ligase (GDP-forming)	EC:6.2.1.4	FL642416, FL643639	Symbiont
		phosphoenolpyruvate carboxykinase (GTP)	EC:4.1.1.32	FL641109, FL642604, FL643416	Symbiont
8	Cysteine and methionine metabolism	cysteine synthase	EC:2.5.1.47	FL643652	Symbiont
9	Drug metabolism - other enzymes	dihydropyrimidine dehydrogenase (NADP+)	EC:1.3.1.2	FL641335	Symbiont
10	Fructose and mannose metabolism	6-phosphofructokinase	EC:2.7.1.11	FL641526	Symbiont
		6-phosphofructokinase	EC:2.7.1.11	FL642304	Symbiont
		diphosphate---fructose-6-phosphate 1-phosphotransferase	EC:2.7.1.90	FL642304	Symbiont
		triose-phosphate isomerase	EC:5.3.1.1	FL642787	Symbiont
		fructose-bisphosphate aldolase	EC:4.1.2.13	FL644124	Symbiont
11	Galactose metabolism	6-phosphofructokinase	EC:2.7.1.11	FL641526	Symbiont
		6-phosphofructokinase	EC:2.7.1.11	FL642304	Symbiont
12	Glycerolipid metabolism	triacylglycerol lipase	EC:3.1.1.3	FL636678	Host
13	Glycolysis/Gluconeogenesis	phosphoenolpyruvate carboxykinase (GTP)	EC:4.1.1.32	FL641109, FL642604, FL643416	Symbiont
		6-phosphofructokinase	EC:2.7.1.11	FL641526	Symbiont
		6-phosphofructokinase	EC:2.7.1.11	FL642304	Symbiont
		triose-phosphate isomerase	EC:5.3.1.1	FL642787	Symbiont
		glucose-6-phosphate isomerase	EC:5.3.1.9	FL643307	Symbiont
		phosphoglycerate kinase	EC:2.7.2.3	FL643941	Symbiont
		fructose-bisphosphate aldolase	EC:4.1.2.13	FL644124	Symbiont
		phosphopyruvate hydratase	EC:4.2.1.11	FL645458	Symbiont
		phosphopyruvate hydratase	EC:4.2.1.11	FL645652	Symbiont
14	Inositol phosphate metabolism	triose-phosphate isomerase	EC:5.3.1.1	FL642787	Symbiont
15	Methane metabolism	6-phosphofructokinase	EC:2.7.1.11	FL641526	Symbiont
		6-phosphofructokinase	EC:2.7.1.11	FL642304	Symbiont
		fructose-bisphosphate aldolase	EC:4.1.2.13	FL644124	Symbiont
		ferredoxin hydrogenase	EC:1.12.7.2	FL644639	Symbiont
		phosphopyruvate hydratase	EC:4.2.1.11	FL645458	Symbiont
		phosphopyruvate hydratase	EC:4.2.1.11	FL645652	Symbiont
16	Nitrogen metabolism	NADH:ubiquinone reductase (H + −translocating)	EC:1.6.5.3	FL645309	Symbiont
17	Oxidative phosphorylation	NADH:ubiquinone reductase (H + −translocating)	EC:1.6.5.3	FL645309	Symbiont
18	Pantothenate and CoA biosynthesis	dihydropyrimidine dehydrogenase (NADP+)	EC:1.3.1.2	FL641335	Symbiont
19	Pentose phosphate pathway	6-phosphofructokinase	EC:2.7.1.11	FL641526	Symbiont
		6-phosphofructokinase	EC:2.7.1.11	FL642304	Symbiont
		glucose-6-phosphate isomerase	EC:5.3.1.9	FL643307	Symbiont
		fructose-bisphosphate aldolase	EC:4.1.2.13	FL644124	Symbiont
20	Propanoate metabolism	succinate---CoA ligase (ADP-forming)	EC:6.2.1.5	FL641410, FL643051, FL644288, FL645720	Symbiont
		succinate---CoA ligase (GDP-forming)	EC:6.2.1.4	FL641410, FL643051, FL644288, FL645720	Symbiont
		succinate---CoA ligase (GDP-forming)	EC:6.2.1.4	FL641015, FL643186	Symbiont
		succinate---CoA ligase (GDP-forming)	EC:6.2.1.4	FL642416, FL643639	Symbiont
21	Purine metabolism	adenosinetriphosphatase	EC:3.6.1.3	FL643974, FL644936, FL645181	Symbiont
		adenosinetriphosphatase	EC:3.6.1.3	FL642059, FL644213, FL645137	Symbiont
		adenosinetriphosphatase	EC:3.6.1.3	FL644210	Symbiont
		adenosinetriphosphatase	EC:3.6.1.3	FL644425	Symbiont
22	Pyrimidine metabolism	dihydropyrimidine dehydrogenase (NADP+)	EC:1.3.1.2	FL641335	Symbiont
		dihydroorotate dehydrogenase (quinone)	EC:1.3.5.2	FL641335	Symbiont
23	Pyruvate metabolism	malate dehydrogenase (oxaloacetate-decarboxylating) (NADP+)	EC:1.1.1.40	FL642239, FL642831, FL645272, FL645325	Symbiont
		malate dehydrogenase (oxaloacetate-decarboxylating)	EC:1.1.1.38	FL642239, FL642831, FL645272, FL645325	Symbiont
		phosphoenolpyruvate carboxykinase (GTP)	EC:4.1.1.32	FL641109, FL642604, FL643416	Symbiont
24	Starch and sucrose metabolism	phosphorylase	EC:2.4.1.1	FL642617	Symbiont
		glucose-6-phosphate isomerase	EC:5.3.1.9	FL643307	Symbiont
		cellulase	EC:3.2.1.4	FL645330	Symbiont
25	Steroid biosynthesis	cholesterol Delta-isomerase	EC:5.3.3.5	FL645075	Symbiont
26	Synthesis and degradation of ketone bodies	hydroxymethylglutaryl-CoA synthase	EC:2.3.3.10	FL641384	Symbiont
27	T cell receptor signaling pathway	phosphoprotein phosphatase	EC:3.1.3.16	FL643487	Symbiont
28	Terpenoid backbone biosynthesis	hydroxymethylglutaryl-CoA synthase	EC:2.3.3.10	FL641384	Symbiont
29	Valine, leucine and isoleucine degradation	hydroxymethylglutaryl-CoA synthase	EC:2.3.3.10	FL641384	Symbiont

Pathways were obtained from sequences corresponding to upregulated spots from the array. Annotations were done with BLAST2GO. The table also indicates the library of origin (host/symbiont) of the sequences.

### Effects of SHE (soldier head extract)

Three and 5 SHE up- and downregulated array positions, respectively, were identified. Each of the 3 upregulated ESTs was unique, *i.e.,* they did not form any contig at 90% similarity, whereas the 5 downregulated ESTs contiged into 3 unique sequences. In the upregulated set, 2 out of 3 ESTs were of host origin while all 3 downregulated contig sequences were of symbiont origin (Table 1, Figure 1). Only 1 host EST out of the 3 upregulated ESTs and 3 symbiont downregulated ESTs could be annotated with BLASTx searches of the nr database (Additional file 1: Table S2A, S2B).

### Effects of live reproductives (LR)

Ten and 3 LR up- and down regulated array positions were identified. Only 2 upregulated host ESTs formed contigs while all other ESTs were unique. In the upregulated set, 6 (out of 9) ESTs were of host origin while all 3 sequences in the downregulated set were of symbiont origin (Table 1, Figure 1). Among the upregulated ESTs, 4 host ESTs and 1 symbiont EST could be annotated by BLASTx against the nr database (Additional file 1: Table S3A); whereas all 3 downregulated symbiont ESTs had nr database matches (Additional file 1: Table S3B).

### Effects of live soldiers (LS)

The presence of live soldiers had a more pronounced impact on worker gut gene expression. A total of 37 and 6 LS up- and downregulated array positions, respectively, were identified (Figure 1). These 43 array positions contiged into 12 host and 6 symbiont-derived up- and downregulated sequences, respectively. The ESTs in the downregulated set did not form any contigs and only 1 out of these 6 was of host origin. Among the upregulated ESTs, 6 and 4 host and symbiont ESTs, respectively, could be annotated by BLASTx against the nr database through BLAST2GO. In the downregulated set, only 2 symbiont ESTs set could be annotated (Additional file 1: Table S4A and B).

### Gene ontology and KEGG pathway analyses with BLAST2GO and DAVID

Gene Ontology (GO) terms were obtained through BLAST2GO and were performed in the three categories *Molecular Function*, *Cellular Location* and *Biological Process* for the JH, SHE, LR and LS datasets, with results for each passing EST shown in Additional file 1: Table S1-S4. In addition, *Molecular Function* GO results were tallied across all 4 treatment categories and are summarized in Table 4. As expected, the highest numbers of GO-*Molecular Function* terms were in the JH up- and downregulated categories (411 and 91 terms). In most other treatments categories, numbers of passing ESTs had proportional numbers of associated GO-*MolecularFunction* terms; however, the SHE downregulated category, which had only 2 passing ESTs (*dna replication licensing factor mcm7* and *serine/threonine-protein kinase mph1*), had 19 GO-*Molecular Function* terms.

**Table 4:** Summary of molecular function gene ontology (GO) terms obtained for annotated expressed sequence tags (ESTs) with BLASTx for treatments with juvenile hormone (JH), soldier head extract (SHE), exposure to live soldiers (LS) and exposure to live reproductive (LR)

**Molecular function**	**JH**		**SHE**		**LS**		**LR**		
**Term**	**Up**	**Down**	**Up**	**Down**	**Up**	**Down**	**Up**	**Down**	**Totals**
Nucleotide binding	8	86		3	3	1			101
Hydrolase activity	4	65		1	1		1		72
Protein binding	7	60		1	1				69
Catalytic activity	17	39			1				57
Binding	15	41					1		57
Structural molecule activity	3	37			1				41
Peptidase activity	10	14					1		25
Transferase activity	3	14		1					18
Enzyme regulator activity	2	12							14
Protein kinase activity	1	10		1		1			13
Kinase activity	2	9		1					12
Calcium ion binding	1	10							11
Electron carrier activity	2	6							8
Transporter activity	3	1			1				5
Actin binding	2	3							5
Carbohydrate binding	2	3							5
Atpase activity					4				4
ATP binding				2	2				4
Receptor activity	3								3
Lipid binding	2	1							3
Nucleoside-triphosphatase activity				1	2				3
Structural constituent of chitin-based cuticle	2								2
Triglyceride lipase activity	2								2
DNA binding				1				1	2
Metal ion binding					1				1
Zinc ion binding					1				1
ATP-dependent DNA helicase activity				1					1
Chitin binding								1	1
DNA helicase activity				1					1
DNA-dependent atpase activity				1					1
GTP binding				1					1
Gtpase activity				1					1
Protein serine/threonine kinase activity				1					1
Single-stranded DNA binding				1					1
Structural constituent of cuticle			1						1
**Totals**	**91**	**411**	**1**	**19**	**18**	**2**	**3**	**2**	**547**

The total numbers of molecular functions obtained from ESTs upregulated by LS and downregulated by SHE show an interesting contrast, which supports the LS and SHE + JH bioassay and gene expression results of Tarver *et al*. [7,12,18]. UP, up-regulated terms, DOWN, down-regulated terms.

Next, KEGG pathway analysis was carried out on the JH dataset to understand the general effects of JH (Tables 2 and 3) on cellular metabolism in the gut transcriptome. Only the JH dataset was examined because of the relatively large numbers of genes available as compared with the SHE, LR and LS datasets. All annotated upregulated ESTs were of host origin, while all but one of the downregulated ESTs were of symbiont origin. Aldehyde dehydrogenase and peroxidase enzymes were abundant in the upregulated dataset and found to be involved in various pathways that included intermediary and amino acid metabolism and cuticle biosynthesis (Table 2). The downregulated KEGG dataset was much more diverse but most notably included enzymes and pathways linked to terpenoid biosynthesis (*e.g.*, *HMG Co-A reductase*; Table 3).

Finally, the program DAVID [27] was used for further bioinformatic analyses of ESTs with identifiable homologs in *Drosophila melanogaster*. DAVID utilizes the background annotation information from various sequenced genomes to assign enrichment scores. Out of the 74 and 332 annotated up- and downregulated ESTs, respectively, 50 and 156 *D. melanogaster* homologs were obtained. (Additional file 1: Table S1A, and S1B). This result suggests JH-related pathways are well conserved between *D. melanogaster* and *R. flavipes*. We present three sets of analyses with JH treatment which show enrichment of GO-*Molecular Function* terms in the sequences that were downregulated from both host and symbiont libraries and upregulated from the host library. From the symbiont library there were only three annotated ESTs that were upregulated, which proved insufficient for enrichment scores. Additional file 1: Table S1C shows the GO-*Molecular Function* terms that were enriched from the upregulated host fraction. In general, molecular functions related to iron binding, peptidase activity and cuticle formation were enriched. However, molecular functions related to peptidase activity were also enriched among the downregulated host ESTs (Additional file 1: Table S1D). Clearly, ESTs with similar molecular functions were both up- and downregulated by JH treatment; however, further details cannot be elucidated by the present analyses. The downregulated sequences from the symbiont fraction, conversely, show a clear trend of shutting down vital cellular functions like translation, transcription and other enzymatic activities (Additional file 1: Table S1E). Whether this reflects the capability of the protists to detect the presence of JH and the ensuing molt of the workers towards non-feeding pre-soldier phenotypes, or other host functions which suppress their cellular activities, remains to be investigated.

### Candidate genes of interest

The JH microarray dataset contained 501 significantly differentially expressed EST contigs (Table 1, Additional file 1: Table S1A, B). The most highly JH up- and downregulated of these ESTs encoded homologs of a host *50 kDa midgut protein* (*50MGP*), which was upregulated 2.9-fold with JH treatment, and a protist symbiont *cysteine synthase a*, which was downregulated 3.1-fold with JH treatment. Other highly upregulated genes in the JH dataset included *nli interacting factor-likephosphatase*, *Arylsulfatase B*, and several apolipoproteins, chymotrypsins and serine proteases. Two *cytochrome P450* homologs from families 6 and 4 were upregulated in the JH dataset and five others from families 6 and 4 were downregulated. Genes related to cuticle formation were upregulated by JH, including *larval* and *pupal cuticle proteins*, *resilin*, *Tyramine beta hydroxylase* and *Dopamine N-acetyl transferase*. The JH dataset also contained a number of upregulated host genes with links to phosphate related post-translational modification, *e.g.*, *nli interacting factor-likephosphatase*, seven de-phosphorylating phosphatases, seventeen kinases (predicted to add phosphate groups), and an insulin receptor homolog with predicted kinase activity.

SHE arrays revealed only 6 ESTs with significant differential expression. The most highly SHE up- and downregulated transcripts were an un-annotated host gene upregulated 2.0-fold and a symbiont *serine/threonine-protein kinase mph1* homolog that was 1.4-fold downregulated. Also, a DNA replication factor, *dna replication licensing factor mcm7*, was downregulated 1.2-fold by SHE treatment.

LR arrays revealed 12 significantly differentially expressed ESTs. The two most highly LR upregulated genes were both from the host and had ~2.2-fold increased expression; the first was a *serine protease 13* homolog, and the second had no significant database matches. The most LR downregulated gene (1.6-fold) encoded a symbiont *linker histone h1 and h5 family* protein.

LS arrays revealed 25 differentially expressed ESTs. With 2.7-fold up-regulation, a host-derived *venom allergen 3-like* homolog with predicted protease functions was the most highly LS upregulated EST. Five additional upregulated ESTs had predicted carbohydrate-active and immune functions; these ESTs included two *alpha amylase* homologs (2.0- to 2.4-fold), two *lysozyme* homologs (1.4 and 1.5-fold), and a *C-type lectin* homolog (1.5-fold). The most downregulated ESTs in the LS dataset included 1 host and 1 symbiont EST with no significant database matches.

### 50 kDa midgut protein: cDNA sequence and amino acid translations

As noted above, the *50MGP* gene was the most highly JH upregulated gene identified in this study. The full length *50MGP* cDNA was assembled from six overlapping EST contigs [28] and verified by database searches and independent resequencing as described under Methods. The *50MGP* cDNA and translated amino acid sequences (Genbank Accession No. KC751537) are provided in Additional file 2: Figure S1. The cDNA sequence contains at least 60 base pairs (bp) of 5′ untranslated region (UTR) before the ATG start codon, a 1419-bp open reading frame, and a 3′ UTR of 133 bp between the “taa” termination codon and the first base of the poly-A tail. The 3′ UTR also contains an “aataa” polyadenylation signal 20 bp upstream of the poly-A tail.

The translated *50MGP* protein sequence had 473 amino acids (AA) and begins with a 19-AA secretory signal peptide “MKTQAILIAAVALLLGTEG”, indicating the mature protein is soluble and secreted. The mature protein without signal peptide contains 454 AA with a predicted mass of 49.9 kDa and isoelectric point (pI) of 7.09. The translated AA sequence has 17 predicted phosphorylation sites on 10 Ser, 4 Thr and 3 Tyr residues (see gray shading in Additional file 2: Figure S1). There are no predicted glycosylation sites. The *50MGP* protein has predicted function as a cell-envelope enzyme with lyase and/or ligase activity. Gene ontology categories predicted for the full length AA sequence, from most to least probable, are “immune response”, “stress response” and “signal transduction.”

**Figure 2:**
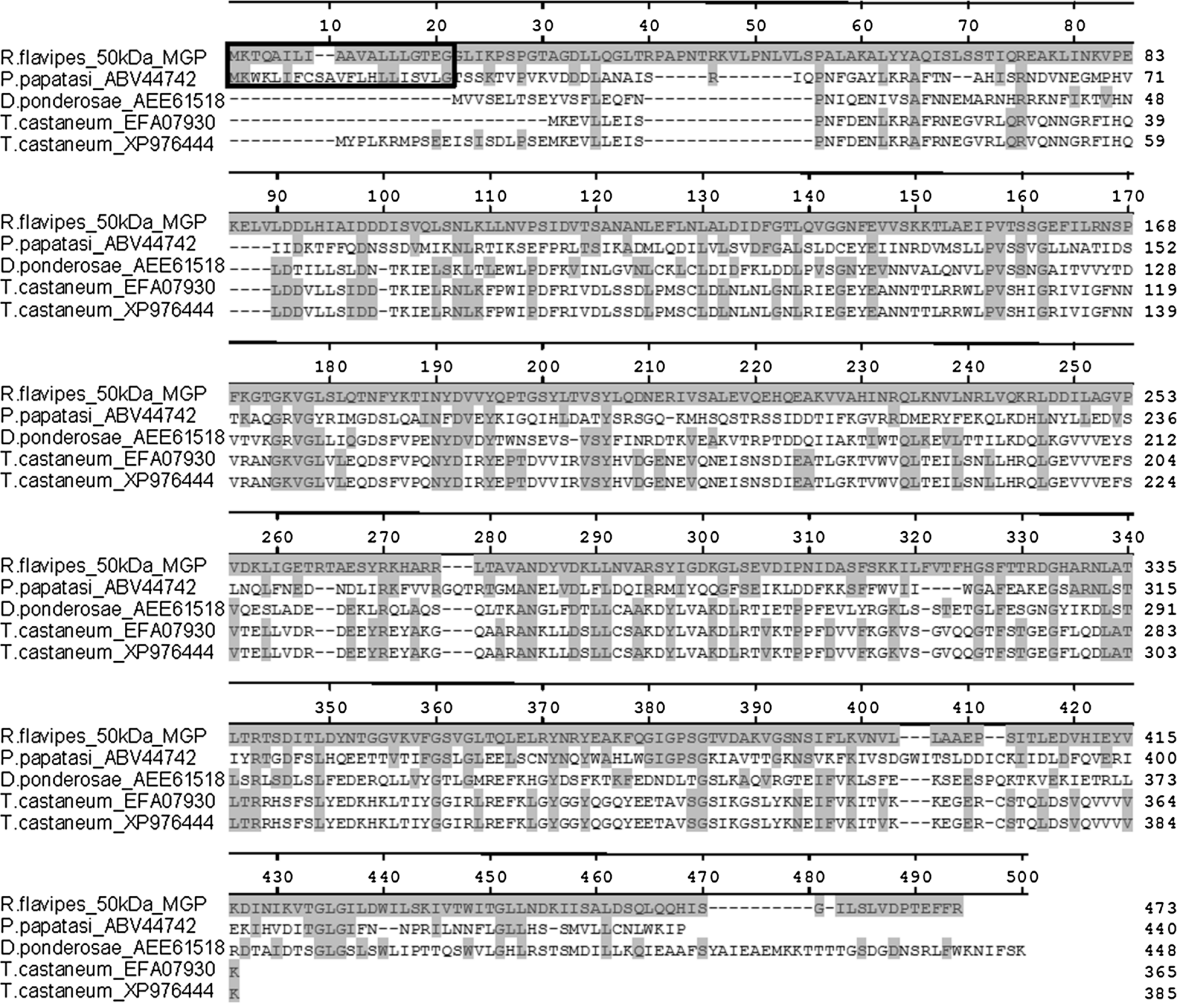
**A ClustalW multiple alignment of homologous *50 kDa Midgut Protein *sequences from various insects, including *R. flavipes *(current study), *Phlebotomous papatasi *(GenBank accession No. ABV44742), *Dendroctonus ponderosae *(AEE61518) and *Tribolium castaneum *(EFA 07930, XP976444). ** Gray shaded amino acids are identical to the *R. flavipes* sequence. Signal peptides for the *R. flavipes* and *P. papatasi* sequences are enclosed in boxes.

A ClustalW alignment of homologous 50MGP proteins from various insects is shown in Figure 2. The alignment contained all homologs that could be identified by BLASTx searches of the GenBank nr database as of Nov 2012. Included in the alignment were homologs sharing 20-35% AA identity from *R. flavipes*, the sandfly *Phlebotomous papatasi*, the bark beetle *Dendroctonus ponderosae*, and the red flour beetle *Tribolium castaneum* (two homologs). There were 38 invariant AA residues in the alignment, with *P. papatasi* sharing the most identity with *R. flavipes* (24.5%), followed by *D. ponderosae* (22.8%), and *T. castaneum* (22.2 and 21.6%). All homologs are equally rich in phosphorylation sites. The sandfly *50MGP* is most similar to the termite *50MGP* protein; it shares a secretion signal peptide, predicted cell envelope interaction, and predicted ligase/lyase, immune, stress response and signal transduction functions with the termite protein.

### Validation of microarray results by qRT-PCR

#### Correlation analysis

**Figure 3:**
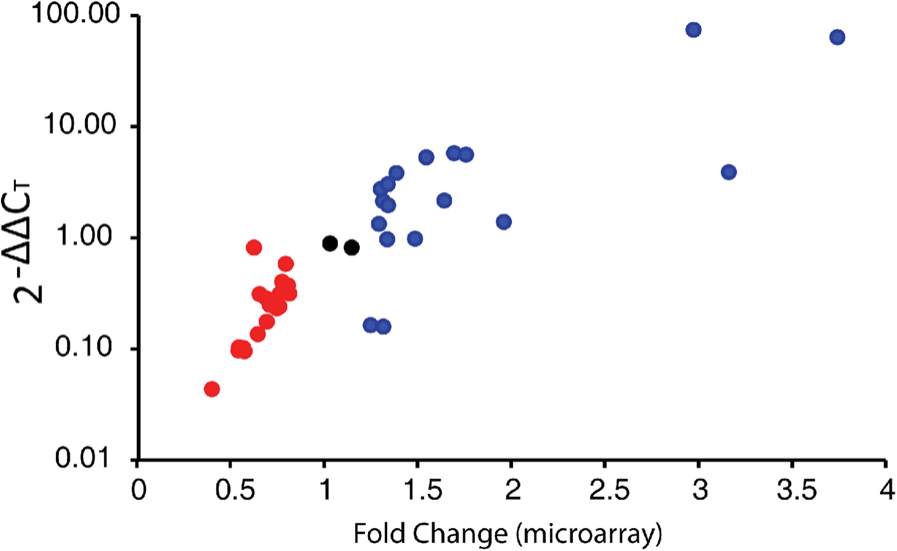
**Significant positive correlation between fold change and threshold cycle (C**_**T**_**) values (Spearman rank correlation, R**_**s**_ **= 0.874, N = 40, *P *<0.001).** Each data point represents 2^-ΔΔ^C_T_ of quantitative real-time PCR performed on a subset of sequences to verify the robustness of microarray results. Red and blue spots represent down- and upregulated transcripts, respectively. The black spots represent transcripts that were neutral to the treatment.

A subset of 52 ESTs was used to validate relative expression levels determined from microarray hybridization data (Figure 3). This subset included 18 and 22 JH up- and downregulated ESTs, 1 and 2 SHE up- and downregulated ESTs, 3 and 6 LR up- and downregulated ESTs, and 6 LS upregulated ESTs. Template cDNA used in these qRT-PCR reactions was reverse-transcribed from the original RNA samples used for microarray hybridizations. A test of correlation was carried out between the 2^-ΔΔ^C_T_ values (obtained from qPCR C_T_ values) and microarray fold change values of the JH up- and downregulated ESTs. The ΔΔC_T_ values were positively correlated (Spearman Rank Correlation, R_s_ = 0.874, *P* <0.001) with array fold change values as expected since higher fold change indicates presence of more transcripts, which provides smaller C_T_ values. The sample sizes for other treatments were too small for conducting statistical tests; however, ΔΔC_T_ values for all but one tested ESTs showed similar negative correlation trends (*i.e.*, there were negative ΔΔC_T_ values for upregulated ESTs and positive ΔΔC_T_ values for downregulated ESTs, Additional file 3: Table S5).

### “Follow-up” bioassays investigating select candidate genes in JH, SHE and JH + SHE treatments

**Figure 4:**
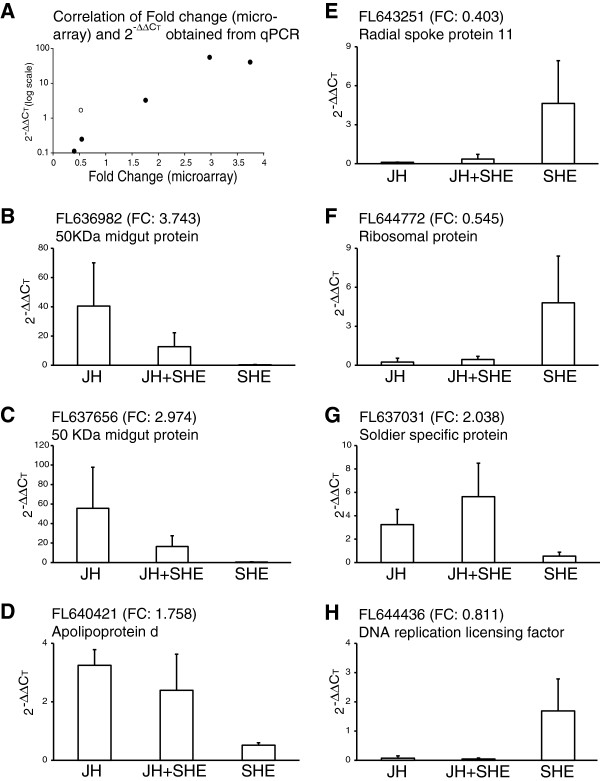
**qPCR reassessment for seven ESTs after treatments with JH, JH + SHE and SHE from the follow-up bioassay. A)** Shows a positive correlation of qPCR results from follow-up bioassays against microarray fold change results for the six JH influenced ESTs (Spearman rank correlation, R_s_ = 0.89, df = 5, P = 0.02). The empty circle represents an EST that was downregulated by both JH and SHE. **B-H)** Show expression profiles for three JH upregulated ESTs **(B**, **C** and **D)**, two JH downregulated ESTs **(E**, **F** and **H)**, one SHE downregulated EST **(G)** and one SHE upregulated EST **(H)**. The expression level is presented as 2^-ΔΔ^C_T,_ calculated from qPCR cycle threshold value. The microarray fold change values are provided in the parentheses for each EST. FL644436 **(G)** was downregulated by both JH (fold change: 0.543) and SHE (fold change: 0.811).

We also reassessed the microarray results using qRT-PCR with cDNA samples from “follow-up” bioassays that exposed new worker termites to JH and SHE for 1 day. Additionally, a combination treatment of JH + SHE that was not included in microarray studies was evaluated. SHE extracts were prepared as described previously and verified by HPLC [7]; Additional file 2: Figure S2). ESTs tested in this experiment included *50MGP* (two ESTs; FL636982 and FL636656), *Apolipoprotein d* (FL640421), *Radial Spoke Protein* (FL643521), *Unknown Ribosomal Protein* (FL644772), *DNA Replication Licensing Factor* (FL644436), and *SoldierSpecific Protein* “*NtSp1-like*” (FL637031). First, a correlation analysis comparing follow-up bioassay results showed a high degree of correlation with microarray results (R_s_ = 0.874; Figure 4A). Second, expression levels for all ESTs examined showed generally opposite effects between JH and SHE treatments, *i.e.*, when transcript levels were upregulated by JH they were downregulated by SHE, and vice-versa (Figure 4B-4G). With the exception of the *DNA Replication Licensing Factor* (FL644436) and *Soldier Specific Protein* “*NtSp1-like*” ESTs, expression levels in JH + SHE treatments were always intermediate between JH and SHE treatments (Figure 4B-4F). Also, the two *50MGP* ESTs showed highly similar expression profiles (Figure 4B & C). However, only the two *50MGP* ESTs showed significant variation among treatments (Kruskal-Wallis test, *P* < 0.05); nevertheless, we avoid over-interpreting this result due to our small sample sizes (3 biological replicates). The latter *50MGP* results also provide additional support that these two ESTs represent portions of the same cDNA.

## Discussion

### Overview

This study used a microarray-based approach to compare the effects of a morphogenetic hormone (JH), soldier-derived primer pheromones (SHE), live reproductives (LR) and live soldiers (LS) on worker gut gene expression. We focused on worker termites because in lower termites like *R. flavipes* (i) workers compose >90% of colonies, (ii) their guts house both eukaryotic and prokaryotic symbionts, (iii) they are responsible for the majority of lignocellulose digestion, and (iv) workers are totipotent juveniles that retain the capacity to differentiate into both soldier and reproductive caste phenotypes [1,20,29,30]. Our central hypothesis is that the worker gut and its eukaryotic symbionts are potential molecular determinants of termite caste differentiation, eusocial polyphenism and, ultimately, social structure.

We found that variable numbers of expressed genes were affected among the four treatment categories (Table 1). JH treatment had the largest impact on gut gene expression (501 total ESTs affected), followed by LS (24 ESTs), LR (12 ESTs) and SHE (6 ESTs). The JH induced change is consistent with JH’s well-established ability to induce worker-to-soldier caste differentiation in lower termites [4], as well as presoldier induction assays in which 90-91% JH-induced presoldier differentiation was observed on a sub-sample of two colonies (see *Results*). Interestingly, the majority of JH upregulated genes were from the host termite, whereas the majority of JH downregulated genes were from protist symbionts. While the complement of upregulated host genes seems to provide insights into caste-regulatory physiology (discussed below), the down-regulation of symbiont genes by JH seems more likely an indicator of protist susceptibility to host hormones or a purging of symbionts in response to rising hormone titers [8,31]. The comparatively weak impact of SHE on gut gene expression is consistent with its lack of impacts on caste differentiation when applied alone [12,18]. Similarly, the greater impact on gene expression in LS treatments is consistent with the more pronounced impacts of live soldiers on limiting JH-dependent caste differentiation [7,18]. Finally, the unexpectedly small number of genes impacted in LR treatments is consistent with worker-derived apterous neotenic reproductives being relatively uncommon in *Reticulitermes* colonies [32], as well as mounting evidence of a genetic (rather than environmental) basis for reproductive differentiation in lower termites [33-36]. However, it may also be possible the 12 genes identified in LR treatments are mediating volatile primer pheromone signals coming from *Reticulitermes* neotenic females [37].

As shown by volcano plots (Figure 1), the SHE, LR and LS treatments had a number of upregulated array positions with large magnitudes of change but a lack of statistical significance. These profiles imply physiological heterogeneity among the test worker populations for individuals that are responsive to SHE, reproductive and soldier-based cues, and suggest a need for targeted research to better understand physiological variability among individuals in termite colonies. Also, as a contrast to directly comparing numbers of differentially expressed genes among the four treatment categories, GO analyses were performed to gain insights based on predicted cellular location, biological process and molecular function of differentially expressed genes. In particular, GO-*Molecular Function* analyses revealed some notable trends (Table 4). First, despite having only two passing genes, the SHE downregulated category had 19 GO-*Molecular Function* terms suggesting broad pleiotropic impacts by these two passing genes. Also, the numbers of GO-*Molecular Function* terms in the SHE downregulated and LS upregulated categories are consistent with previous bioassay results showing contrasting impacts by these two treatments (*i.e.,* SHE + JH induces higher levels of caste differentiation, and live soldiers limit JH-dependent caste differentiation [7,12,18]).

Regarding the small expression fold changes obtained in the current study, we conclude this to be a result of the microarray-based platform that was used. This conclusion is based on the larger fold-change expression magnitudes obtained using qPCR relative to the microarray platform in the current study (Tables 2 and 3, Figure 4), as well as two previous studies that compared feeding impacts on gut gene expression using microarrays and quantitative pyrosequencing (showing higher expression by qPCR and pyrosequencing [26,39]). In the sections that follow, we further discuss candidate gene trends, related genes passing in multiple treatment categories, insights into termite social regulation and symbiosis, and overall conclusions.

### Candidate gene trends

The most highly JH up- and downregulated ESTs encoded homologs of a host *50 kDa midgut protein* “*50MGP*” and a protist symbiont *cysteine synthase a*. The *cysteine synthase a* transcript was also the most highly abundant transcript identified previously with paper (cellulose) feeding [26], which was the control condition (with acetone) in the current study. Cysteine synthases catalyze production of acetate, which is an important metabolic intermediate in the termite gut [38]. The *50MGP* gene is novel to the current study and discussed under Conclusions.

A majority of JH downregulated ESTs were of symbiont origin and are considered indicators of general symbiont decline in response to JH treatment. Most notably, the list of downregulated symbiont genes includes five GHF7 cellulases. Because GHF7 cellulases play important roles in *R. flavipes* digestion [26,39], this trend suggests compromised digestive capabilities in association with JH-induced caste morphogenesis. The remaining JH downregulated symbiont ESTs are not discussed hereafter but can be found in Table 3.

The second and third most highly upregulated ESTs in the JH dataset were of host origin and included a *nli interacting factor-likephosphatase* with predicted protein dephosphorylation function and an *Arylsulfatase B* homolog with predicted functions in cleaving sulfate groups from glycosaminoglycans, including N-acetyl monosaccharides that compose gut integument [40]. Other noteworthy genes in the JH upregulated dataset include two *Apolipoproteins* and three *chymotrypsin/serine proteases* with potential roles in lipid- and protease-based developmental signaling similar to JH responsive proteases identified previously [41,42]. Additionally, two *cytochromeP450* homologs from families 6 and 4 were upregulated in the JH dataset and five others from the same families were downregulated. Interestingly, the cytochrome P450 *Cyp15F1*, which has been implicated in JH-dependent presoldier induction in *R. flavipes* and is inducible in the gut by wood feeding [6,26], was not impacted by JH treatment in the current study.

ESTs related to cuticle formation were also upregulated with JH treatment, including *larval* and *pupal cuticle proteins*[21,43], *resilin*[44], and enzymes potentially regulating melanin biosynthesis such as *Tyramine beta hydroxylase* and *Dopamine N-acetyl transferase*[45,46]. Possibly related to the high number of phosphorylation sites predicted on the translated *50MGP* protein, the JH dataset also contained a number of upregulated ESTs with links to phosphate-related post-translational modification; for example, (i) the *nli interacting factor-likephosphatase* noted above, (ii) other phosphatases potentially involved in de-phosphorylation, (iii) kinases potentially involved in protein phosphorylation, and (iv) an *insulin receptor* homolog [47] with predicted kinase activity.

The most highly SHE upregulated EST was a host gene with no significant GenBank nr database match; however, translated searches of the GenBank EST database revealed numerous un-annotated homologs of this EST in other termite and cockroach cDNA libraries. The most SHE downregulated EST was a symbiont *serine/threonine-protein kinase mph1* homolog related to mitotic function [48]. Also, an important DNA replication factor, *DNA replication licensing factor mcm7*[49], was downregulated by SHE treatment. These two SHE downregulated genes had a large number GO-*Molecular Function* terms associated with them, suggesting broad pleiotropic impacts.

The two most highly LR upregulated ESTs were from the host termite; one with no database matches and the other was a *serine protease 13* homolog. The most downregulated EST in the LR dataset encoded a symbiont *Linker histone h1 and h5 family protein* involved in DNA binding and regulation of chromatin structure [50].

Finally, the most highly LS upregulated EST was a *venom allergen 3-like* homolog, similar to a venom protease from the ant *Solenopsis invicta*[51]. Other highly upregulated host ESTs from LS arrays had links to carbohydrate hydrolysis/binding and immune function. These ESTs included *alpha amylase* homologs, *lysozyme* homologs from GHF 13 and 22, and a *C-type lectin* homolog from CBM Family 13 [52]. The most downregulated ESTs in the LS dataset included one host and one symbiont EST, neither of which had significant database matches.

### Similar ESTs passing in multiple microarray treatment categories

Kinases were a highly represented gene family in the JH dataset and other kinases also appeared in the LS, LR and SHE datasets. Of these kinases, only a *serine/threonine-protein kinase mph1* homolog appeared in more than one dataset (JH and SHE), and was downregulated in both cases. An *Arylsulfatase B* homolog was highly induced in the JH dataset, and was also upregulated in the LS dataset. A *c-type lectin precursor* presumably involved in carbohydrate binding was upregulated in the JH and LS datasets. A number of protease, peptidase and/or chymotrypsin homologs were differentially expressed among the JH, LR and LS datasets; however, while each of these datasets had highly differentially expressed protease-related ESTs, the composition among each dataset was unique. Nonetheless, it is noteworthy that JH-responsive proteases have previously been identified in insect guts [41]. Lastly, host *alpha amylase* homologs were upregulated in the LS and LR datasets, but all were distinct. Thus, in summary, major functional protein categories sampled across treatment categories include kinases, proteases, and various carbohydrate active proteins (amylases, arylsulfatases, and lectins).

### New insights into termite social regulation and symbiosis

Although JH and SHE may not be ingested by termites in nature, the simple technique of placing them on JH or SHE-treated diet ensured intake of JH and SHE by the termites. We expect that JH and SHE are ingested and absorbed by the cuticles of the termites exposed to treated filter paper. Also, because trophallaxis plays such a large role in termite sociality [1], this study in part, investigates what could be happening if termites were to acquire JH by trophallaxis [53]. We found significant JH-dependent expression changes in gut-associated genes from both the host termite and protist symbionts, as well as significant long-term presoldier induction in JH bioassays conducted on a sub-sample of colonies used in the current study (see Results). These correlated gene expression and phenotypic changes were elicited after just one day of treatment, illustrating the strong impacts of JH even after a short exposure. JH is a known caste-regulatory hormone, and termites are known to go through morphological changes after experimental JH exposure [4]; however, the mechanisms by which JH influences gene expression in termites are not well understood. This study takes us one step further in this interpretation. We have shown that JH generally up-regulates host gene expression and generally down-regulates that of protist symbionts. We do not expect that JH directly influences protist gene expression but it can influence the purging of gut contents [8,31], and our results might be indicative of a general decline of protist populations (dissections, performed post-hoc, revealed that some JH treated termites indeed lacked any gut content or had purged guts; results not shown). However, the changes brought upon by the absence of protists in the termite gut also have to be considered. The most significant effect in this respect would likely be on digestion, implying that an absence of regular nutrients could play a role in caste regulation [4,11,54]; for example, several protist GHF7 cellulases were downregulated by JH exposure (Table 3). It should also be noted that our cDNA libraries were made with only gut-associated ESTs; therefore the differentially expressed genes shown here must be only a fraction of the changes triggered by JH treatment in the whole body of termites.

We also found that exposure to SHE and LS triggered expression changes for comparatively small numbers of genes relative to JH. Previous studies showed that SHE and LS by themselves do not elicit any significant impacts on caste differentiation [7,12,18], and the current results are consistent with those findings. Also, relatively small numbers of symbiont genes were downregulated by SHE and LS treatments, supporting the idea that protist populations are not dramatically affected by these factors and thus do not mediate their signals.

## Conclusions

This research took a microarray-based approach to test the hypothesis that the worker termite gut and its eukaryotic protist symbionts are potential molecular determinants of caste differentiation. This study builds on a prior study by Tarver *et al*. [18] that investigated whole-body expression of 49 host genes in response to JH, SHE and LS treatments. Four treatments were investigated in the current study that included JH, SHE, LR and LS. With respect to JH, our hypothesis is strongly supported. However, for SHE, LR and LS treatments our hypothesis is not well supported. These results suggest that, if they have impacts at all, the SHE, LR and LS treatments might (i) be acting more substantially beyond 1-day exposure periods [18], (ii) be acting in other body regions or tissues than gut, and/or (iii) have minimal impacts on expression of genes that influence caste differentiation and caste homeostasis. Alternatively, as suggested by GO-*Molecular Function* analyses, some of the small numbers of gut genes impacted by SHE, LR and LS treatments may actually have broad pleiotropic impacts. Despite these vastly different impacts on gut gene expression among treatments, major functional categories of responsive genes sampled across treatment categories encoded integumental proteins, kinases, proteases, and various carbohydrate-active proteins (amylases, arylsulfatases, and lectins).

This study also revealed a novel *50MGP* cDNA as the most highly JH-inducible transcript in the *R. flavipes* gut. Multiple ESTs for this gene assembled into an apparent full-length cDNA that shares many common features with other host termite cDNAs [28,39]. While the function of the translated *50MGP* is unknown, it is predicted to have a large number of phosphorylation sites, a well-defined secretory signal sequence, and share a predicted JH-binding region with a homologous protein from the bark beetle *Dendroctonus ponderosae*[55]. Other known insect homologs of this protein are from the sand fly *Phlebotomous papatasi*[56] and the red flour beetle *Tribolium castaneum*[57]. The translated *50 kDa protein* has predicted immune, stress response and signal transduction functions; future functional studies will investigate links to these processes and others.

Finally, previous studies testing the JH + SHE combination revealed unexpected increases in soldier caste differentiation relative to treatments with JH alone, whereas treatments with SHE alone had no such impacts [7,12,18]. In the current study, we conducted “follow-up” bioassays and qRT-PCR on 6 significant genes from JH and SHE microarrays to investigate possible interactions. Interestingly, in these treatments JH and SHE had opposite impacts, and in most cases the JH + SHE combination resulted in gene expression intermediate between JH and SHE alone. These results and those of Tarver *et al*. [18] suggest next-generation transcriptome sequencing as a potentially informative approach for investigating whole-body gene expression impacts by JH + SHE treatments, as well as individual SHE components. This study in general provides important new insights into molecular determinants underlying termite caste polyphenism and homeostasis, including symbiont population decline in association with the caste differentiation process.

## Methods

### Colony maintenance and bioassays

All colonies were verified as *R. flavipes* by mitochondrial 16S rRNA sequencing as described by Szalanski *et al*. [58] (data not shown). Colonies were maintained in darkness in sealed plastic boxes containing wet pine wood shims and brown paper towels, within an environmental chamber kept at 22°C and 60-100% relative humidity (RH). Bioassays were conducted in darkness at 27°C and 60-100% RH. The termites used for microarray analysis originated from five established laboratory colonies at the University of Florida, Entomology and Nematology Department, in Gainesville, FL: (1) B1#1 (established 05/20/2009); (2) B2 (06/03/2010); (3) K2 (07/11/2007); (4) K5 (08/02/2008); and (5) K9 (06/29/2010). Bioassays for microarray studies were performed in August-September, 2010. Additionally, JH-presoldier induction bioassays were conducted on workers from one Florida colony used for microarray studies (B1#1) and another from Indiana (WI-9). These bioassays used 100 μg JH III (Sigma-Aldrich; Milwaukee, WI) per assay dish or acetone alone for controls and followed the methods of Tarver *et al*. [7,12], except only a 24-hr exposure was used. Six and four independent replicates per treatment were done for the WI-9 and B1#1 colonies. Finally, we conducted a second “follow-up” bioassay experiment to see the effect of JH, SHE and JH along with SHE on selected genes. Termites used in this bioassay originated from a single colony collected from Purdue University, West Lafayette, IN, USA (“WI-9” established in 2012).

### Hormonal microarrays

The hormonal bioassays were conducted as previously described by Tarver *et al.*[12,18]. Twenty workers (pseudergates) were used per assay and given moist filter paper discs (Whatman #3 filter discs, GE Healthcare Bio-Sciences Corp., Piscataway, NJ) as food. The filter paper discs received either JH III or SHE or acetone (for control). JH III (93% purity; Sigma; St. Louis, MO) was applied on one filter paper disc per assay and at 150 μg per disc in 150 μl acetone. SHE was prepared by homogenizing soldier heads in acetone with a Tenbroeck glass homogenizer and applied at two soldier head equivalents per disc in 150 μl acetone. Acetone (150 μl) was applied on discs for control assays. After applying the solution, acetone was evaporated from the discs for 20 minutes at room temperature prior to each assay. The filter papers were then moistened with 150 μl of distilled water and placed in 3.5-cm diameter Petri dishes with the termites.

### Social treatment microarrays

In social treatments, groups of twenty workers were maintained with either two soldiers or two neotenic reproductives originating from the same colony. Moist filter paper discs were offered as food. Termites were maintained for one day in 3.5-cm diameter Petri dishes in darkness at 27°C and 60-100% RH.

### Gut extraction and RNA isolation

Both social and hormonal treatments were conducted for 24 hr, and the workers were then cold-immobilized, surface-sterilized by a serial rinse in 0.3% sodium hypochlorite (once) and sterilized water (twice), and dissected on Parafilm® to collect digestive tracts including salivary glands. Digestive tracts were transferred into RLA Lysis Buffer (Promega, Madison, WI) and stored at −70°C until RNA isolation. RNA Extraction and cDNA Synthesis was done according to Raychoudhury *et al*. [26].

### Microarray hybridizations

Experiments were designed after MIAME guidelines, and microarray data obtained in these studies were deposited at ArrayExpress [http://www.ebi.ac.uk/arrayexpress (accession number E-MTAB-1417)]. A type II microarray [59] design used with a common-reference strategy [60]. The common reference consisted of a normalized blend of all RNA samples included in the experiment. This common reference was co-hybridized against each replicate sample on single microarrays. Dye swaps [59] were performed between replicate samples and references to check for potential dye impacts on spot intensity. Twenty-five total microarray hybridizations were performed and consisted of each of five colonies treated with JH, SHE, acetone and exposed to live reproductives (LR) and live soldiers (LS).

### Statistical analyses

The Matlab statistics toolbox was used for statistical analysis of the intensity data of 25 hybridizations from five different treatments [JH, SHE, exposure to live reproductives (LR), live soldiers (LS) and acetone (A) for control]. Before comparative analysis, the individual signal intensity values obtained from the microarray probes were log-transformed (using 2 as the base) and normalized among all individual samples included in the study. Normalization was accomplished by scaling the individual log-transformed signal intensities so that each dataset had comparable lower, median and upper quartile values [61]. After the data were normalized, Student’s t-tests were used to make probe-by-probe comparisons among each treatment and control (JH vs. A, SHE vs. A, LR vs. A and LS vs. A). In each comparison, a *p*-value and fold change was computed for all microarray loci. In addition to *p*-values, *q*-values were computed [62]. While the *p*-value measures the minimum statistical false positive rate incurred when setting a threshold for test significance, the *q*-value measures the minimum false discovery rate incurred when calling that test significant [62]. A volcano plot for each comparison was generated that displays the negative log_10_-transformed *p*-value versus log_2_-transformed fold change for each array locus (Figure 1).

### Bioinformatic analyses

Contig generation: All significantly differentially expressed array positions that met the fold change criteria in each bioassay were selected and processed through Sequencher (Gene Codes Corporation, Ann Arbor, MI) with a minimum match percentage of 95 to generate contigs. The generated contigs and the remaining orphan sequences were used for further analyses.

Gene Ontology analysis using BLAST2GO: The selected contigs and the orphan sequences were analyzed using the program BLAST2GO [63] for identification and annotation. By using the inbuilt BLASTx algorithm, these sequences were used as queries in BLASTx searches against the GenBank non-redundant (nr) database with an *e*-value cut-off of ≤ 1e-03 (last performed 06, 2012). The putative identification, annotation, and Gene Ontology (GO) terms [64] for the sequences were also obtained through BLAST2GO. JH, SHE, LR and LS influenced sequences are listed in supporting information Additional file 1: Tables S1-S4.

### Validation of microarray fold change data by quantitative real-time PCR

The fold change data from the microarray results were validated by performing sets of quantitative real-time PCRs (qRT-PCR) with a CFX-96 Real-time System (Bio-Rad, Hercules, CA) using the SYBR green detection method (SensiMix SYBR & Fluorescein one-step PCR reagent; Bioline, Taunton, MA). Fifty-two different sequences (Additional file 3: Table S6) with varying degrees of fold change were used to design primer sequences using the web-based tool Real-time Design (http://www.biosearchtech.com/realtimedesign). The housekeeping gene *lim-1* was used as a reference gene [18,26]. Two μl of total RNA (from aliquots of 10 ng/μl) were taken from the original mRNA pools used for microarray hybridizations from all five colonies (5 treatments each) to synthesize cDNA using the iScript cDNA kit (Bio-Rad, Hercules, CA). Triplicate qRT-PCR reactions were performed for each of the 25 sets of cDNA samples, along with a no-cDNA negative control, across the 52 primer sets (Additional file 3: Table S6). Cycling conditions were an initial step of 95°C for 3 minutes followed by 39 cycles of 95°C for 20 seconds, 56°C for 45 seconds and 68°C for 50 seconds. Quantification was performed by first generating a standard curve of primer amplification efficiency, using whole-gut cDNA from colony B1#1 with a five-fold dilution series, and then extrapolating the experimental samples onto the curve. Each triplicate sample was averaged to one data point for ease of graphical representation. The mean delta threshold cycle (ΔC_T_) was calculated for each data point by subtracting it from the average C_T_ values of *lim-1*. Then a ΔΔC_T_ value was calculated by subtracting average acetone data point from JH, SHE, LR or LS (see formula below for JH). These ΔΔC_T_ values were plotted against the corresponding fold change levels from the microarray studies, and a correlation test was conducted.

ΔΔCT=15∑j=1513∑i=13PJHi−13∑i=13lim1JHi−15∑j=1513∑i=13PAi−13∑i=13lim1JHi

j = number of biological replicates,

i = number of technical replicates,

P = given primer, *lim1* = *lim1* primer;

JH_i_ = C_T_ value of the ith technical replicate from the JH treated termite gut cDNA,

A_i_ = C_T_ value of the ith technical replicate from the acetone treated termite gut cDNA

### “Follow-up” bioassays

Follow-up bioassays were conducted with JH III and freshly made SHE. The SHE was analyzed on HPLC to confirm whether the chemical peaks, and thus overall composition, matched with previous studies of Tarver *et al.*[7]. As Additional file 2: Figure S2 shows, the composition of SHE matched with that used in prior work demonstrating SHE transfer and efficacy by Tarver *et al.*[7]. Filter paper discs were soaked with one of the following: 200 μl acetone (control), JH III (65% purity; Santa Cruz Biotechnology Inc, Santa Cruz, CA) at 100 μg/μl of acetone, SHE (2 head equivalents in 100 μl acetone) and a combination of JH III (100 μg/100 μl of acetone) and SHE (2 head equivalent in 100 μl acetone). Then all filter papers were air dried and moistened with 150 μl of distilled water before offering to the termites in 6.5-cm diameter Petri dishes. Twenty workers were used per assay with three replicates for each treatment. Individual guts were extracted into RNA lysis buffer, and RNA was extracted as described above. cDNAs were produced using the iScript cDNA synthesis kit (Bio-Rad, Hercules, CA). These cDNAs were used for qPCR with *lim1* and seven other primers that were selected due to very high or very low fold change ratios from JH and SHE microarray data (Figure 4). The ΔC_T_ values (for control and treatment) acquired from the qPCR were used to calculate the 2^-ΔΔC^_T_ values to compare the results for different treatments.

### 50 kDa Midgut protein sequencing and sequence analysis

The full-length cDNA sequence for the *50MGP* gene was assembled from five overlapping clones/ESTs from a previously described gut cDNA library (GenBank accession nos. FL639806, FL636982, FL638011, FL638525 and FL637656 from Tartar *et al.*[28]). The contig sequence (GenBank no. KC751537) was verified by alignments having >99% identity with seven ESTs obtained from different *R. flavipe*s phenotype cDNA libraries [65]. Portions of the ORF sequence were further verified by PCR amplification of cDNA fragments spanning nucleotides 41–537, 366–1104, 510–807 and 656–1126, using the forward and reverse primers described in Additional file 3: Table S6. Database searches were performed at NCBI (http://www.ncbi.nlm.nih.gov/) using BLASTn and BLASTx under default settings. Peptide translations were made using SDSC Biology Workbench (http://workbench.sdsc.edu/). Signal peptide, N-glycosylation, phosphorylation, protein functional category and enzyme class analyses were performed using the SignalP, NetNGlyc 1.0, NetPhos and ProtFun servers available at http://www.cbs.dtu.dk/services/. Protein alignments were generated using ClustalW in the Lasergene software package (DNAstar; Madison, WI).

## Competing interests

The authors declare that they have no competing interests.

## Authors’ contributions

RS and RR did the bioinformatic analyses, ran qRT-PCRs, surveyed the library fidelity, did statistical analyses and drafted the paper. MES conceived, designed and conducted the experiments and participated in drafting the manuscript. VUL and DGB participated in the original design of the experiment and conducted bioassays and RNA isolation for the microarray. YC and YS analysed the microarray data. All authors read and approved the final manuscript.

## Supplementary Material

Additional file 1 **Identity, fold change and gene ontology terms for passing host and symbiont genes from JH microarrays (Additional file **1**: Table S1-S4).** Click here for file

Additional file 2 **50 kDa midgut protein translation (Additional file **2**: Figure S1) and HPLC analysis of soldier head extract (Additional file **2**: Figure S2).** Click here for file

Additional file 3 **Summary of ΔΔC**_**T **_**values for repeat bioassay qPCRs (Additional file**3**: Table S5) and the list of primers used for qPCR validations (Additional file**3**: Table S6).** Click here for file
