# Supplementary material for: Differential impacts of juvenile hormone, soldier head extract and alternate caste phenotypes on host and symbiont transcriptome composition in the gut of the termite Reticulitermes flavipes
Source: BMC Genomics. 2013 Jul 19;14:491. doi: 10.1186/1471-2164-14-491 (PMC3731027; doi:10.1186/1471-2164-14-491)
Supplement: Additional file 1 — Identity, fold change and gene ontology terms for passing host and symbiont genes from JH microarrays (Additional file 1: Table S1-S4). [file 1471-2164-14-491-S1.docx]

Table S1A. JH up-regulated transcripts (Fold change ratio >1.18, with P<0.05). (NSM= No significant matches). Individual sequences and contigs are listed according to fold change. For contigs, average fold change values are provided. The putative sequence identities, gene ontology (GO) and enzyme codes were obtained from BLAST2GO. *Drosophila melanogaster* homologs were obtained by BLASTX.

| **S1A**  **Accession #** | **Putative Sequence Identity** | **Origin** | **Fold Change** | ***Drosophila melanogaster* homolog** | **GOs** | **Enzyme Codes** |
| --- | --- | --- | --- | --- | --- | --- |
| FL639806, FL637656, FL638011, FL638525, FL636982 | 50 kDa midgut protein | Host | 2.87 | NSM | - |  |
| FL635781 | nli interacting factor-like phosphatase family protein | Host | 2.57 | NSM | F:protein binding | - |
| FL638301 | arylsulfatase b | Host | 1.98 | 281363223 | F:catalytic activity; P:metabolic process | - |
| FL635557 | chymotrypsin-like protein | Host | 1.84 | NSM | F:peptidase activity; P:protein metabolic process; P:catabolic process | EC:3.4.21.0 |
| FL637599 | NSM | Host | 1.78 | NSM | - |  |
| FL640421 | outer membrane lipoprotein | Host | 1.76 | NSM | F:binding; P:transport; F:transporter activity; F:lipid binding; C:extracellular space; C:extracellular region; C:intracellular; F:lipid transporter activity; P:lipid metabolic process; P:lipid transport; F:protein binding |  |
| FL643739 | NSM | Symbiont | 1.75 | NSM | - |  |
| FL638790, FL638853, FL639359, FL640796 | NSM | Host | 1.73 | NSM | - |  |
| FL635023, FL637475, FL637852, FL637163 | apolipoprotein d-like | Host | 1.73 | NSM | F:binding | - |
| FL637296, FL636675 | jonah 65aiv | Host | 1.71 | 21358103 | F:peptidase activity | - |
| FL639349 | NSM | Host | 1.69 | NSM | - |  |
| FL638855 | serine proteinase stubble | Host | 1.64 | 19921666 | F:peptidase activity; P:protein metabolic process; P:catabolic process | EC:3.4.21.0 |
| FL638138, FL639027 | gly-rich protein | Host | 1.64 | NSM | - |  |
| FL635037 | NSM | Host | 1.63 | NSM | - |  |
| FL635011, FL637865, FL636990, FL635573 | c-type lectin precursor | Host | 1.63 | 28574695 | F:carbohydrate binding | - |
| FL636683, FL636699 | NSM | Host | 1.61 | NSM | - |  |
| DN792534 | larval cuticle protein | Host | 1.55 | 24639977 | F:structural molecule activity | - |
| Nymph_F11_T3_Not_in_GB | similar to AGAP010676-PA (*Tribolium castaneum*) | Host | 1.51 | NSM | F:molecular_function; P:biological_process; C:cellular_component |  |
| FL635645 | carboxypeptidase a-like | Host | 1.49 | 24583126 | F:peptidase activity; P:protein metabolic process; P:catabolic process | - |
| FL642595 | surface antigen -like | Symbiont | 1.48 | NSM | C:integral to membrane; C:membrane; C:plasma membrane |  |
| FL643436 | ser thr protein phosphatase family protein | Symbiont | 1.46 | 17136620 | P:multicellular organismal development; P:biological_process; F:protein binding; C:protein complex; C:intracellular; C:cytoplasm; C:chromosome; C:cytoskeleton; C:organelle; P:protein modification process; P:regulation of biological process; P:metabolic process; F:phosphoprotein phosphatase activity | - |
| FL638576 | serine 3-dehydrogenase | Host | 1.45 | 24641388 | P:metabolic process; F:catalytic activity; F:nucleotide binding | - |
| FL639795 | NSM | Host | 1.44 | NSM | - |  |
| FL641208 | xyppx repeat family protein | Symbiont | 1.41 | NSM | C:integral to membrane; C:membrane |  |
| FL639290 | plasma kallikrein-like | Host | 1.41 | 24657332 | F:peptidase activity; P:protein metabolic process; P:catabolic process | EC:3.4.21.0 |
| FL637610 | NSM | Host | 1.39 | NSM | - |  |
| FL637839, FL639576, FL636023 | NSM | Host | 1.39 | NSM | - |  |
| FL640754 | cytochrome p450 | Host | 1.39 | 24653734 | F:electron carrier activity; F:binding; P:metabolic process; F:catalytic activity | - |
| FL638880, FL639091, FL640694 | cytochrome p450 | Host | 1.38 | 17864130 | F:electron carrier activity; F:catalytic activity; F:binding; C:cell; P:metabolic process | - |
| FL639333 | NSM | Host | 1.38 | NSM | - |  |
| FL640223 | Hypothetical protein EAI_03143 (*Harpegnathos saltator*) | Host | 1.37 | NSM | C:membrane; P:ion transport |  |
| FL639210, FL636446 | NSM | Host | 1.35 | NSM | - |  |
| FL636115 | NSM | Host | 1.35 | NSM | - |  |
| FL640793 | NSM | Host | 1.35 | NSM | - |  |
| FL639931 | male sterility domain-containing | Host | 1.34 | 24654209 | F:catalytic activity; P:metabolic process | - |
| FL640448 | tyramine beta hydroxylase | Host | 1.34 | 28571135 | F:binding; P:cellular amino acid and derivative metabolic process; F:catalytic activity; P:metabolic process | EC:1.14.17.1 |
| FL638015 | NSM | Host | 1.33 | NSM | - |  |
| FL635347 | peptide methionine sulfoxide reductase | Host | 1.33 | 320545892 | F:catalytic activity; P:metabolic process; P:protein modification process | EC:1.8.4.12; EC:1.8.4.11 |
| FL639319 | ankyrin repeat domain-containing protein 17 | Host | 1.32 | NSM | F:protein binding | - |
| FL637993 | conserved Hypothetical protein (*Pediculus humanus corporis*) | Host | 1.31 | NSM | F:protein binding | - |
| FL640037 | zinc metalloproteinase | Host | 1.31 | 24643089 | F:peptidase activity | - |
| FL637824, FL638311, FL638494, FL636440, FL636362, FL637273, FL637330 | chondroitin proteoglycan-2 | Host | 1.31 | 19920772 | F:carbohydrate binding; C:extracellular region; P:carbohydrate metabolic process | - |
| FL638529 | eukaryotic translation initiation factor 4e binding protein | Host | 1.30 | 17137440 | F:protein binding; P:regulation of biological process; P:translation | - |
| FL636096 | guanine nucleotide-binding protein subunit beta-like | Host | 1.29 | 17137396 | F:receptor activity; F:kinase activity; P:metabolic process; P:signal transduction | - |
| FL637586, FL639041 | resilin isoform a | Host | 1.29 | 24654243 | F:structural constituent of cuticle; F:structural constituent of chitin-based cuticle; P:biological_process |  |
| FL635983 | lipase 3 | Host | 1.28 | 320544939 | P:lipid metabolic process; F:hydrolase activity; F:triglyceride lipase activity |  |
| CB518316 | NSM | Host | 1.28 | NSM | - |  |
| FL640757 | NSM | Host | 1.28 | NSM | - |  |
| FL639457, FL641215, FL643726 | major allergen Cr-PII (*Periplaneta americana*) | Host | 1.28 | NSM | - |  |
| FL636185 | predicted protein (*Nematostella vectensis*) | Host | 1.27 | 8161 | - |  |
| FL635431 | microtubule-associated protein jupiter-like | Host | 1.27 | 24646001 | F:cytoskeletal protein binding; C:cytoskeleton; C:protein complex | - |
| FL637749, FL639100, FL640426 | NSM | Host | 1.27 | NSM | - |  |
| FL635432 | NSM | Host | 1.27 | NSM | - |  |
| FL636966 | NSM | Host | 1.26 | NSM | - |  |
| FL636667 | NSM | Host | 1.26 | NSM | - |  |
| FL638856 | short-chain dehydrogenase | Host | 1.25 | 24640492 | F:catalytic activity; P:metabolic process; C:lipid particle; F:nucleotide binding | - |
| FL635609 | NSM | Host | 1.25 | NSM | - |  |
| FL641309 | succinyl- ligase | Symbiont | 1.25 | 21356231 | C:protein complex; C:mitochondrion; P:metabolic process; C:organelle; C:cytoplasm; P:generation of precursor metabolites and energy; P:catabolic process; F:nucleotide binding; F:protein binding; F:catalytic activity | EC:6.2.1.4 |
| FL637666 | pupal cuticle protein 20 | Host | 1.25 | 19922620 | F:structural molecule activity | - |
| FL640193 | inosine-uridine preferring nucleoside hydrolase | Host | 1.25 | 24641837 | F:hydrolase activity; F:hydrolase activity, hydrolyzing N-glycosyl compounds; P:biological_process; C:cellular_component |  |
| FL636340 | angiotensin-converting enzyme 2 | Host | 1.24 | 17137008 | P:biological_process; P:regulation of biological process; F:binding; F:peptidase activity; C:cell; P:protein metabolic process; P:catabolic process | EC:3.4.15.0 |
| FL645378 | NSM | Symbiont | 1.24 | NSM | - |  |
| FL641351 | cell surface protein | Symbiont | 1.24 | NSM | F:GTP binding |  |
| FL639659 | type-1 angiotensin ii receptor-associated | Host | 1.24 | NSM | F:receptor activity |  |
| FL637591, FL639354 | NSM | Host | 1.24 | NSM | - |  |
| FL636090 | lipase 1 | Host | 1.23 | 320544939 | P:lipid metabolic process; F:triglyceride lipase activity |  |
| FL638461 | aldehyde dehydrogenase | Host | 1.23 | 157738865 | C:cytosol; F:enzyme regulator activity; P:metabolic process; P:signal transduction; F:catalytic activity; F:lipid binding; P:lipid metabolic process; P:secondary metabolic process; P:biosynthetic process; P:carbohydrate metabolic process; P:generation of precursor metabolites and energy; P:catabolic process; P:cellular amino acid and derivative metabolic process | EC:1.2.1.36; EC:1.2.1.3 |
| FL639154, FL636006, FL636984, FL637448, FL640573 | Predicted protein (*Nematostella vectensis*) | Host | 1.23 | NSM | F:binding | - |
| FL638191 | aldehyde dehydrogenase | Host | 1.23 | 157738865 | F:catalytic activity; P:metabolic process; P:lipid metabolic process; P:secondary metabolic process | EC:1.2.1.36 |
| FL636719, , FL635394 | NSM | Host | 1.23 | NSM | - |  |
| FL638976, FL639406 | secreted ferritin g subunit | Host | 1.23 | 24651364 | P:ion transport; P:cellular homeostasis; F:binding; F:catalytic activity; P:metabolic process | - |
| FL638218, FL638231, FL639028, FL639150, FL639388, FL637293 | NSM | Host | 1.23 | NSM | - |  |
| CB518310 | NSM | Host | 1.23 | NSM | - |  |
| FL642856 | glyceraldehyde-3-phosphate dehydrogenase | Symbiont | 1.23 | 22023983 | C:cytoplasm; F:nucleotide binding; P:carbohydrate metabolic process; P:generation of precursor metabolites and energy; P:catabolic process; F:catalytic activity; P:metabolic process; P:biosynthetic process | EC:1.2.1.12 |
| FL638691, FL640056, FL636038 | serine proteinase stubble | Host | 1.22 | 19921666 | F:peptidase activity; P:protein metabolic process; P:catabolic process | EC:3.4.21.0 |
| FL638664, FL636501, FL636357, FL635501 | fk506-binding protein | Host | 1.22 | 17352457 | P:protein metabolic process; F:catalytic activity; F:calcium ion binding; P:behavior; F:binding | EC:5.2.1.8 |
| FL637528, FL638782, FL639330 | uncharacterized protein loc100187734 | Host | 1.22 | NSM | - |  |
| FL637476 | aspartate partial | Host | 1.22 | 19922362 | P:cellular amino acid and derivative metabolic process; F:transferase activity; P:biosynthetic process; F:binding | EC:2.6.1.0 |
| FL636458 | zinc metalloproteinase nas-13-like | Host | 1.22 | 24643089 | F:peptidase activity; P:protein metabolic process; P:catabolic process; F:binding | EC:3.4.24.0 |
| FL637485 | uncharacterized protein LOC100160144 (*Acyrthosiphon pisum*) | Host | 1.21 | NSM | C:spindle; C:nucleus; C:microtubule associated complex; C:microtubule; C:cytoplasm; P:positive regulation of microtubule polymerization; F:structural constituent of cytoskeleton; F:microtubule binding; C:cytosol; C:cytoskeleton |  |
| FL638281 | gdp-l-fucose synthase | Host | 1.21 | 19922778 | F:catalytic activity; P:cell death; P:biosynthetic process; P:carbohydrate metabolic process; P:nucleobase, nucleoside, nucleotide and nucleic acid metabolic process; F:binding; P:metabolic process; F:nucleotide binding | EC:1.1.1.271 |
| FL636297 | Hypothetical protein KGM_13894 (*Danaus plexippus*) | Host | 1.21 | 24640916 | - |  |
| FL637649 | cold-induced protein | Host | 1.21 | NSM | - |  |
| FL637359 | NSM | Host | 1.21 | NSM | - |  |
| FL638213 | eukaryotic translation initiation factor 3 subunit g | Host | 1.21 | NSM | F:nucleotide binding; F:translation factor activity, nucleic acid binding; C:ribosome; P:regulation of biological process; P:translation | - |
| FL636468 | Dentin matrix protein 4 (*Camponotus floridanus*) | Host | 1.21 | 221458707 | - |  |
| FL635183, FL635468, FL640726 | dopamine n isoform b | Host | 1.21 | 45552817 | P:behavior; F:transferase activity; F:transporter activity; P:cellular amino acid and derivative metabolic process; P:biosynthetic process; P:transport | - |
| FL639781, FL639987, FL640150 | i-type lysozyme | Host | 1.21 | 22024182 | F:hydrolase activity; P:carbohydrate metabolic process | EC:3.2.1.17 |
| FL637633, FL638043 | leukocyte elastase inhibitor-like | Host | 1.21 | 24585522 | P:protein metabolic process; P:regulation of biological process; P:catabolic process; P:biological_process; F:enzyme regulator activity | - |
| FL638164 | pavarotti | Host | 1.21 | 17136974 | P:cytoskeleton organization; P:signal transduction; P:biological_process; C:cytoskeleton; P:cell cycle | - |
| FL642032 | xyppx repeat family protein | Symbiont | 1.20 | NSM | C:integral to membrane; C:membrane |  |
| FL637713 | insulin receptor | Host | 1.20 | 24648768 | F:protein kinase activity; F:receptor activity; P:protein modification process; F:nucleotide binding; C:cell; P:signal transduction | EC:2.7.10.1 |
| FL637172 | chorion peroxidase-like | Host | 1.20 | NSM | F:antioxidant activity; F:catalytic activity; P:response to stress; F:binding; P:metabolic process | EC:1.11.1.7 |
| FL637044 | NSM | Host | 1.20 | NSM | - |  |
| FL640317 | sodium-coupled monocarboxylate transporter 2-like | Host | 1.20 | 19920916 | F:transporter activity; C:cell; P:transport | - |
| FL645220 | Hypothetical Protein TTHERM_00584930 (*Tetrahymena thermophila*) | Symbiont | 1.20 | NSM | F:kinase activity; P:phosphorylation; F:transferase activity; F:dextransucrase activity; F:transferase activity, transferring glycosyl groups |  |
| FL643383 | NSM | Symbiont | 1.20 | NSM | - |  |
| FL636417 | NSM | Host | 1.20 | NSM | - |  |
| FL643233 | NSM | Symbiont | 1.20 | NSM | - |  |
| FL636560 | NSM | Host | 1.20 | NSM | - |  |
| FL635998 | NSM | Host | 1.20 | NSM | - |  |
| FL638629 | NSM | Host | 1.20 | NSM | - |  |
| FL639621, FL639829 | angiotensin-converting enzyme (dipeptidyl carboxypeptidase) | Host | 1.20 | 17137008 | P:regulation of biological process; P:protein metabolic process; P:catabolic process; F:catalytic activity; P:biological_process; P:response to external stimulus; F:binding; F:nucleotide binding; P:multicellular organismal development; C:cell; F:peptidase activity; F:receptor binding; P:transport; C:extracellular space; F:actin binding; C:plasma membrane; C:endosome; P:cell differentiation; P:biosynthetic process; P:nucleobase, nucleoside, nucleotide and nucleic acid metabolic process; P:response to stress; P:anatomical structure morphogenesis; P:cell proliferation | EC:4.6.1.2; EC:3.4.15.0 |
| FL639045 | calponin transgelin | Host | 1.20 | 21355917 | F:actin binding; P:cytoskeleton organization | - |
| FL637933 | ribosomal protein l10ae | Host | 1.19 | 24662946 | F:structural molecule activity; F:RNA binding; P:translation; P:nucleobase, nucleoside, nucleotide and nucleic acid metabolic process; P:biological_process; C:ribosome | - |
| FL639955 | cauliflower | Host | 1.19 | NSM | P:transcription, DNA-dependent; F:protein binding; P:floral meristem determinacy; P:positive regulation of flower development; C:nucleus; F:transcription factor activity; F:DNA binding |  |
| FL636198 | secreted ferritin s subunit | Host | 1.19 | 24651362 | P:cellular homeostasis; F:binding; F:catalytic activity; P:ion transport; P:metabolic process | EC:1.16.3.1 |
| FL637002 | NSM | Host | 1.19 | NSM | - |  |

Table S1B. JH down-regulated transcripts (Fold change ratio <0.84, with P<0.05). (NSM= No significant matches). Individual sequences and contigs are listed according to fold change. For contigs, average fold change values are provided. The putative sequence identities, gene ontology (GO) and enzyme codes were obtained from BLAST2GO. *Drosophila melanogaster* homologs were obtained by BLASTX.

| **S1B**  **Accession #** | **Putative Sequence Identity** | **Origin** | **Fold Change** | ***Drosophila melanogaster* homolog** | **GOs** | **Enzyme Codes** |
| --- | --- | --- | --- | --- | --- | --- |
| FL643872 | NSM | Symbiont | 0.84 | NSM | - |  |
| FL639922 | NSM | Host | 0.84 | NSM | - |  |
| FL635838 | conserved plasmodium protein | Host | 0.84 | NSM | F:nucleic acid binding; F:nucleotide binding; F:transferase activity; F:binding; P:regulation of ARF protein signal transduction; F:ARF guanyl-nucleotide exchange factor activity; C:intracellular |  |
| FL642225 | NSM | Symbiont | 0.84 | NSM | - |  |
| FL644508 | proline-rich protein 13 | Symbiont | 0.84 | NSM | F:metal ion binding; F:zinc ion binding |  |
| FL639420 | dual oxidase maturation factor 1-like | Host | 0.84 | 19921356 | C:endoplasmic reticulum; P:protein transport; C:cell | - |
| FL635626 | cytochrome p450 | Host | 0.84 | 17864130 | F:electron carrier activity; F:binding; P:metabolic process; F:catalytic activity | - |
| FL639002 | lysosomal alpha-mannosidase (mannosidase alpha class 2b member 1) | Host | 0.84 | 24647249 | F:carbohydrate binding; P:carbohydrate metabolic process; F:hydrolase activity | - |
| FL642823 | g-box binding factor | Symbiont | 0.83 | NSM | C:chromatin; F:helicase activity; F:chromatin binding; F:hydrolase activity; F:DNA binding; C:nucleus; F:nucleic acid binding; P:chromatin assembly or disassembly; F:ATP binding; C:membrane; C:plasma membrane; F:protein kinase activity; P:protein amino acid phosphorylation; F:unfolded protein binding; P:protein folding; F:heat shock protein binding |  |
| FL642796 | NSM | Symbiont | 0.83 | NSM | - |  |
| FL638288, FL639566 | serine protease | Host | 0.83 | 17986085 | - |  |
| FL635796 | NSM | Host | 0.83 | NSM | C:extracellular region; P:carbohydrate metabolic process; F:carbohydrate binding | - |
| FL644310 | cellulose-binding family ii | Symbiont | 0.83 | NSM | P:carbohydrate metabolic process; F:hydrolase activity; F:carbohydrate binding | - |
| FL644338 | adp-ribosylation factor 1 | Symbiont | 0.83 | 17864182 | F:signal transducer activity; C:Golgi apparatus; F:protein binding; P:viral reproduction; F:nucleotide binding; P:cellular homeostasis; P:nucleobase, nucleoside, nucleotide and nucleic acid metabolic process; P:catabolic process; C:cytosol; P:regulation of biological process; P:response to stress; P:response to biotic stimulus; P:signal transduction; P:transport; C:cytoplasm; F:hydrolase activity; C:plasma membrane; P:organelle organization; P:protein transport | - |
| FL637811 | small GTP-binding protein (*Trichomonas vaginalis* G3) | Host | 0.83 | 17137088 | F:nucleotide binding; P:signal transduction; F:hydrolase activity; P:nucleobase, nucleoside, nucleotide and nucleic acid metabolic process; P:catabolic process; P:protein transport; P:transport; C:intracellular; C:cell | - |
| FL643344 | Hypothetical protein (*Trichomonas vaginalis* G3) | Symbiont | 0.83 | NSM | - |  |
| FL640219 | late trypsin | Host | 0.83 | 17648133 | F:peptidase activity; P:protein metabolic process; P:catabolic process | EC:3.4.21.0 |
| FL637356 | NSM | Host | 0.83 | NSM | - |  |
| FL643185 | metal-dependent protein hydrolase | Symbiont | 0.83 | NSM | - |  |
| FL638116, FL639895, FL635192, FL636514, FL636992 | calpain-2 catalytic subunit | Host | 0.83 | 28574468 | F:protein binding; P:protein metabolic process; P:catabolic process; F:peptidase activity; C:intracellular | - |
| FL639946 | NSM | Host | 0.83 | NSM | - |  |
| FL637045 | NSM | Host | 0.83 | NSM | - |  |
| FL641162, FL643116 | RNA-binding protein musashi homolog rbp6-like | Symbiont | 0.83 | 62484464 | F:nucleic acid binding; F:nucleotide binding | - |
| FL641517, FL645106 | RNA-binding protein musashi homolog rbp6-like | Symbiont | 0.83 | 62484464 | F:nucleic acid binding; F:nucleotide binding | - |
| FL643201 | NSM | Symbiont | 0.83 | NSM | - |  |
| FL644077, FL644689 | uba ts-n domain containing protein | Symbiont | 0.82 | NSM | F:protein binding | - |
| FL637464, FL638806, FL637696, FL637715, FL638437, FL638688 | CG10407-PA-like protein (*Blattella germanica*) | Host | 0.82 |  | - |  |
| FL643065 | alpha-tubulin | Symbiont | 0.82 | 17136564 | C:cytoskeleton; C:protein complex; P:cellular component organization; P:nucleobase, nucleoside, nucleotide and nucleic acid metabolic process; P:catabolic process; P:biological_process; F:hydrolase activity; F:structural molecule activity; C:cytoplasm; F:nucleotide binding | - |
| FL641195 | adp-ribosylation factor 1 | Symbiont | 0.82 | 17864182 | F:signal transducer activity; C:Golgi apparatus; F:protein binding; P:viral reproduction; F:nucleotide binding; P:cellular homeostasis; P:nucleobase, nucleoside, nucleotide and nucleic acid metabolic process; P:catabolic process; C:cytosol; P:regulation of biological process; P:response to stress; P:response to biotic stimulus; P:signal transduction; P:transport; C:cytoplasm; F:hydrolase activity; C:plasma membrane; P:organelle organization; P:protein transport | - |
| FL641650 | alpha-tubulin 1 | Symbiont | 0.82 | 17136564 | C:cytoskeleton; C:protein complex; P:cellular component organization; P:nucleobase, nucleoside, nucleotide and nucleic acid metabolic process; P:catabolic process; P:biological_process; F:hydrolase activity; F:structural molecule activity; C:cytoplasm; F:nucleotide binding | - |
| FL643953 | polyubiquitin | Symbiont | 0.82 | 361584485 | C:intracellular; F:protein binding | - |
| FL643282 | translation initiation factor if-3 | Symbiont | 0.82 | NSM | F:translation factor activity, nucleic acid binding; P:translation | - |
| CB518317ot_in_GBot_in_GB | NSM | Host | 0.82 | NSM | - |  |
| FL642442 | NSM | Symbiont | 0.82 | NSM | - |  |
| FL645162 | NSM | Symbiont | 0.81 | NSM | - |  |
| FL637722 | beta tubulin | Host | 0.81 | 158739 | C:cytoskeleton; C:protein complex; P:cellular component organization; P:nucleobase, nucleoside, nucleotide and nucleic acid metabolic process; P:catabolic process; P:biological_process; F:hydrolase activity; F:structural molecule activity; F:nucleotide binding | - |
| FL641335 | dihydropyrimidine dehydrogenase | Symbiont | 0.81 | NSM | C:cytosol; P:nucleobase, nucleoside, nucleotide and nucleic acid metabolic process; P:metabolic process; C:cell; P:response to external stimulus; F:catalytic activity; P:biological_process; P:response to endogenous stimulus; F:nucleotide binding; F:protein binding; F:binding; P:catabolic process; P:biosynthetic process; P:cellular amino acid and derivative metabolic process | EC:1.3.1.2; EC:1.3.5.2 |
| FJ184563, FJ184567, FJ184572, FJ184577, FJ184582, FJ184587, FJ18459, 1FJ184596, FJ184600 | termicin | Host | 0.81 | NSM | - |  |
| FL639685 | retinol dehydrogenase 11-like | Host | 0.81 | 24640117 | P:metabolic process; F:catalytic activity | - |
| FL637416 | NSM | Host | 0.81 | NSM | - |  |
| FL638690 | NSM | Host | 0.81 | NSM | - |  |
| FL639527, FL635709, FL640326 | serine protease | Host | 0.81 | 17986085 | F:peptidase activity; P:protein metabolic process; P:catabolic process | EC:3.4.21.0 |
| FL645643 | ras-related c3 botulinum toxin substrate 1 (rho small gtp binding protein rac1) | Symbiont | 0.81 | 17136856 | C:plasma membrane; F:nucleotide binding; P:response to external stimulus; P:response to stress; P:viral reproduction; P:regulation of biological process; P:cytoskeleton organization; P:transport; P:cellular component organization; P:biological_process; P:anatomical structure morphogenesis; P:cell differentiation; P:multicellular organismal development; P:embryonic development; C:cell; C:cytosol; P:signal transduction; P:lipid metabolic process; P:cell death; P:response to biotic stimulus; C:Golgi apparatus; P:biosynthetic process; P:DNA metabolic process; C:cytoplasmic membrane-bounded vesicle; P:metabolic process; P:behavior; F:protein binding; F:enzyme regulator activity; P:cell proliferation; P:response to abiotic stimulus; F:hydrolase activity; P:nucleobase, nucleoside, nucleotide and nucleic acid metabolic process; P:catabolic process; P:protein transport | - |
| FL642267 | ef-hand calcium-binding domain-containing protein 10-like | Symbiont | 0.80 | NSM | F:calcium ion binding; F:cAMP-dependent protein kinase regulator activity; P:signal transduction |  |
| FL643895 | NSM | Symbiont | 0.80 | NSM | - |  |
| FL643914 | NSM | Symbiont | 0.80 | NSM | - |  |
| FL641333 | elongation factor-1 partial | Symbiont | 0.80 | 7915 | F:translation factor activity, nucleic acid binding; P:nucleobase, nucleoside, nucleotide and nucleic acid metabolic process; P:catabolic process; F:hydrolase activity; F:nucleotide binding; C:ribosome; P:regulation of biological process; P:translation | - |
| FL637482, FL637655, FL638942, FL638962, FL640250, FL636311, FL637380, FL640405, FL640640 | lipase 3 | Host | 0.80 | 19921102 | F:hydrolase activity | - |
| FL642116 | Hypothetical protein (*Trichomonas vaginalis* G3) | Symbiont | 0.80 | NSM | C:flagellum |  |
| FL643266 | voltage gated chloride channel domain-containing protein | Symbiont | 0.80 | NSM | P:chloride transport; F:voltage-gated chloride channel activity; C:membrane; P:transmembrane transport; F:ion channel activity; P:protein transport; P:protein modification process; C:cell outer membrane; P:oxidation reduction; F:oxidoreductase activity; F:2-alkenal reductase activity |  |
| FL643553, FL643882 | eukaryotic translation elongation factor 2 | Symbiont | 0.80 | 24585711 | C:cytosol; F:translation factor activity, nucleic acid binding; P:nucleobase, nucleoside, nucleotide and nucleic acid metabolic process; P:catabolic process; F:protein binding; F:hydrolase activity; F:nucleotide binding; C:ribosome; P:regulation of biological process; P:translation | - |
| FL642304 | 6-phosphofructokinase | Symbiont | 0.80 | 28573326 | F:kinase activity; C:cytosol; C:protein complex; P:carbohydrate metabolic process; P:generation of precursor metabolites and energy; P:catabolic process; P:biosynthetic process; P:nucleobase, nucleoside, nucleotide and nucleic acid metabolic process; P:secondary metabolic process | EC:2.7.1.11; EC:2.7.1.90 |
| FL643406 | viral a-type inclusion | Symbiont | 0.80 | NSM | C:organelle; C:intracellular; P:biological_process | - |
| FL643042, FL644524 | elongation factor-1 partial | Symbiont | 0.80 | 17137572 | F:translation factor activity, nucleic acid binding; P:nucleobase, nucleoside, nucleotide and nucleic acid metabolic process; P:catabolic process; F:hydrolase activity; F:nucleotide binding; C:ribosome; P:regulation of biological process; P:translation | - |
| FL639066, FL639952 | 17-beta hydroxysteroid dehydrogenase, putative (*Pediculus humanus corporis*) | Host | 0.80 | 24640492 | P:metabolic process; F:catalytic activity; F:nucleotide binding | - |
| FL644135 | dynein heavy chain family protein | Symbiont | 0.80 | NSM | P:microtubule-based movement; F:sequence-specific DNA binding; F:transcription factor activity; P:ATP catabolic process; F:nucleotide binding; F:nucleoside-triphosphatase activity; F:ATP binding; F:ATPase activity; F:microtubule motor activity; C:dynein complex; P:regulation of transcription, DNA-dependent; P:transcription, DNA-dependent |  |
| FL640529, FL642242, FL642763, FL644706 | beta tubulin | Mixed | 0.80 | 158739 | C:cytoskeleton; C:protein complex; P:cellular component organization; P:nucleobase, nucleoside, nucleotide and nucleic acid metabolic process; P:catabolic process; P:biological_process; F:hydrolase activity; F:structural molecule activity; F:nucleotide binding | - |
| FL643993 | Hypothetical protein (*Trichomonas vaginalis* G3) | Symbiont | 0.79 | NSM | F:protein binding | - |
| FL638546 | ump-cmp kinase-like | Host | 0.79 | 17647141 | F:kinase activity; P:nucleobase, nucleoside, nucleotide and nucleic acid metabolic process; F:nucleotide binding | - |
| FL640609 | alpha-tubulin 1 | Host | 0.79 | 17737821 | C:cytosol; P:cellular component organization; C:cytoskeleton; C:protein complex; P:nucleobase, nucleoside, nucleotide and nucleic acid metabolic process; P:catabolic process; P:biological_process; F:protein binding; F:hydrolase activity; F:structural molecule activity; F:nucleotide binding | - |
| FL635381 | beta tubulin | Host | 0.79 | 158739 | C:cytoskeleton; C:protein complex; P:cellular component organization; P:nucleobase, nucleoside, nucleotide and nucleic acid metabolic process; P:catabolic process; P:biological_process; F:hydrolase activity; F:structural molecule activity; F:nucleotide binding | - |
| FL644210 | flagellar inner arm dynein 1 heavy chain alpha | Symbiont | 0.79 | NSM | F:motor activity; C:cilium; C:organelle; C:intracellular; C:cytoskeleton; C:protein complex; P:cellular component organization; P:nucleobase, nucleoside, nucleotide and nucleic acid metabolic process; P:catabolic process; F:nucleotide binding; P:biological_process; F:hydrolase activity | EC:3.6.1.3 |
| FL641463 | beta-tubulin | Symbiont | 0.79 | 24655737 | C:cytoskeleton; C:protein complex; P:cellular component organization; P:nucleobase, nucleoside, nucleotide and nucleic acid metabolic process; P:catabolic process; P:biological_process; F:hydrolase activity; F:structural molecule activity; C:cytoplasm; F:nucleotide binding | - |
| FL642081, FL645543 | ras-related c3 botulinum toxin substrate 1 precursor | Symbiont | 0.79 | 17136856 | C:plasma membrane; F:nucleotide binding; P:regulation of biological process; P:cytoskeleton organization; P:anatomical structure morphogenesis; P:cellular component organization; P:cell differentiation; P:multicellular organismal development; P:transport; P:embryonic development; C:cell; C:cytosol; P:lipid metabolic process; P:cell death; C:Golgi apparatus; P:biosynthetic process; P:DNA metabolic process; C:cytoplasmic membrane-bounded vesicle; P:behavior; P:response to external stimulus; F:protein binding; F:enzyme regulator activity; P:biological_process; P:signal transduction; P:cell proliferation; P:response to stress; P:response to abiotic stimulus; P:metabolic process; F:hydrolase activity; P:nucleobase, nucleoside, nucleotide and nucleic acid metabolic process; P:catabolic process; P:protein transport | - |
| FL641258, FL641495, FL643362, FL645702 | alpha tubulin 1a | Symbiont | 0.79 | 17136564 | P:protein metabolic process; C:cytosol; P:cell cycle; P:cellular component organization; C:cytoskeleton; C:protein complex; P:nucleobase, nucleoside, nucleotide and nucleic acid metabolic process; P:catabolic process; P:biological_process; F:protein binding; F:hydrolase activity; F:structural molecule activity; F:nucleotide binding | - |
| FL635492 | NSM | Host | 0.79 | NSM | F:nucleotide binding; C:cytoskeleton; C:protein complex; P:biological_process; P:cellular component organization | - |
| FL643546 | adenylate kinase 7 | Symbiont | 0.79 | NSM | F:kinase activity; P:metabolic process; F:binding | - |
| FL645123 | c2 domain containing protein | Symbiont | 0.79 | 62484516 | C:cell; F:protein binding | - |
| FL645469 | Similar to predicted protein, partial (*Hydra magnipapillata*) | Symbiont | 0.79 | NSM | C:intracellular; F:binding | - |
| FL637234, FL642373, FL644000, FL644982 | beta tubulin | Mixed | 0.78 | 158739 | C:cytoskeleton; C:protein complex; P:cellular component organization; P:nucleobase, nucleoside, nucleotide and nucleic acid metabolic process; P:catabolic process; P:biological_process; F:hydrolase activity; F:structural molecule activity; F:nucleotide binding | - |
| FL637223, FL641085, FL641671, FL641977 | alpha tubulin 1a | Mixed | 0.78 | 320545892 | P:protein metabolic process; C:cytosol; P:cell cycle; P:cellular component organization; C:cytoskeleton; C:protein complex; P:nucleobase, nucleoside, nucleotide and nucleic acid metabolic process; P:catabolic process; P:biological_process; F:protein binding; F:hydrolase activity; F:structural molecule activity; F:nucleotide binding |  |
| FL636678 | lipase 3 | Host | 0.78 | 320544939 | F:hydrolase activity; P:catabolic process; P:lipid metabolic process | EC:3.1.1.3 |
| FL642707 | homology domain containing protein | Symbiont | 0.78 | NSM | F:phosphoric ester hydrolase activity; F:zinc ion binding |  |
| FL638604 | cytochrome p450 6k1 | Host | 0.78 | 24652913 | C:endoplasmic reticulum; C:cell; F:electron carrier activity; F:catalytic activity; F:binding; P:metabolic process | - |
| FL645517 | 60s ribosomal protein | Symbiont | 0.78 | 21357053 | F:structural molecule activity; P:translation; C:ribosome | - |
| FL643909 | Hypothetical protein (*Trichomonas vaginalis* G3) | Symbiont | 0.78 | NSM | - |  |
| FL636097 | NSM | Host | 0.78 | NSM | - |  |
| FL644184 | Hypothetical protein (*Trichomonas vaginalis* G3) | Symbiont | 0.78 | NSM | - |  |
| FL644348 | NSM | Symbiont | 0.77 | NSM | - |  |
| FL637679, FL638973, FL635317, FL635708 | chymotrypsin 1 | Host | 0.77 | 21358103 | P:metabolic process; F:peptidase activity | - |
| FL641143, FL642323, FL642644 | malate dehydrogenase | Symbiont | 0.77 | 24583394 | F:catalytic activity; P:generation of precursor metabolites and energy; P:catabolic process; P:metabolic process; P:carbohydrate metabolic process; F:nucleotide binding | EC:1.1.1.0 |
| FL642315 | lecithin:cholesterol acyltransferase family protein | Symbiont | 0.77 | NSM | P:lipid metabolic process; F:transferase activity | - |
| FL635363 | alpha-tubulin | Host | 0.77 | 320545892 | C:cytoskeleton; C:protein complex; P:cellular component organization; P:nucleobase, nucleoside, nucleotide and nucleic acid metabolic process; P:catabolic process; P:biological_process; F:hydrolase activity; F:structural molecule activity; C:cytoplasm; F:nucleotide binding | - |
| FL643129 | chromosome segregation atpase-like protein | Symbiont | 0.77 | NSM | - |  |
| FL643378, FL644287 | glycosyl hydrolase family7 | Mixed | 0.77 | NSM | P:carbohydrate metabolic process; F:hydrolase activity | EC:3.2.1.0 |
| FL643316 | PREDICTED: Hypothetical protein LOC100446713 (*Pongo abelii*) | Symbiont | 0.77 | NSM | - |  |
| FL644889 | peptidyl-prolyl cis-trans isomerase | Symbiont | 0.77 | 17647301 | P:protein metabolic process; P:protein modification process; F:catalytic activity | EC:5.2.1.8 |
| FL641350 | coiled-coil domain-containing protein 111-like | Symbiont | 0.77 | NSM | F:transferase activity; P:biosynthetic process; P:DNA metabolic process | - |
| FL641078, FL642144, FL642196, FL642340, FL642655, FL642742, FL642924, FL643885, FL644327, FL644945, FL645287, FL645750 | glycosyl hydrolase family7 | Symbiont | 0.77 | NSM | P:carbohydrate metabolic process; F:hydrolase activity | EC:3.2.1.0 |
| FL645651 | leucine rich repeat family | Symbiont | 0.77 | NSM | F:GTP binding |  |
| FL642258 | NSM | Symbiont | 0.77 | NSM | - |  |
| FL641032 | NSM | Symbiont | 0.77 | NSM | - |  |
| FL643062, FL644351 | glycosyl hydrolase family7 | Symbiont | 0.77 | NSM | P:carbohydrate metabolic process; F:hydrolase activity | EC:3.2.1.0 |
| FL644124 | fructose- -bisphosphate aldolase | Symbiont | 0.76 | NSM | P:carbohydrate metabolic process; P:generation of precursor metabolites and energy; P:catabolic process; F:catalytic activity; F:binding; P:biosynthetic process; P:nucleobase, nucleoside, nucleotide and nucleic acid metabolic process; P:secondary metabolic process; P:metabolic process | EC:4.1.2.13 |
| FL642165 | NSM | Symbiont | 0.76 | NSM | - |  |
| FL641060 | NSM | Symbiont | 0.76 | NSM | - |  |
| FL641253 | translation elongation factor 1 beta | Symbiont | 0.76 | 24583273 | F:enzyme regulator activity; C:ribosome; C:cytoskeleton; C:protein complex; F:translation factor activity, nucleic acid binding; C:cytoplasm; P:biological_process; P:regulation of biological process; P:translation | - |
| FL639157 | cytochrome p450 | Host | 0.76 | 17864130 | F:binding; C:cell; F:catalytic activity; F:electron carrier activity; P:protein metabolic process; P:metabolic process | - |
| FL638775 | NSM | Host | 0.76 | NSM | - |  |
| FL645565 | clan family cathepsin l-like cysteine peptidase | Symbiont | 0.76 | 24653516 | F:peptidase activity; P:protein metabolic process; P:catabolic process | - |
| FL642475, FL645459, FL641094, FL644179, FL644516 | elongation factor-1 partial | Symbiont | 0.76 | 7915 | F:translation factor activity, nucleic acid binding; P:nucleobase, nucleoside, nucleotide and nucleic acid metabolic process; P:catabolic process; F:hydrolase activity; F:nucleotide binding; C:ribosome; P:regulation of biological process; P:translation | - |
| FL643559 | proteasome subunit alpha type-2 | Symbiont | 0.76 | 17737927 | C:protein complex; C:intracellular; P:protein metabolic process; P:catabolic process; F:peptidase activity | EC:3.4.25.0 |
| FL641092 | ankyrin repeat protein (*Trichomonas vaginalis* G3) | Symbiont | 0.76 | NSM | F:metal ion binding; P:oxidation reduction; F:binding; P:cellular amino acid metabolic process; F:oxidoreductase activity; P:metabolic process; F:catalytic activity; F:zinc ion binding |  |
| FL643641 | Hypothetical protein (*Trichomonas vaginalis* G3) | Symbiont | 0.76 | NSM | - |  |
| FL643685, FL644257 | alpha-tubulin 1 | Symbiont | 0.76 | 17136564 | C:cytoskeleton; C:protein complex; P:cellular component organization; P:nucleobase, nucleoside, nucleotide and nucleic acid metabolic process; P:catabolic process; P:biological_process; F:hydrolase activity; F:structural molecule activity; C:cytoplasm; F:nucleotide binding | - |
| FL641461 | nucleotidyltransferase family protein | Symbiont | 0.76 | NSM | P:biosynthetic process; F:transferase activity | EC:2.7.7.0 |
| FL644756 | tetratricopeptide repeat protein 21b | Symbiont | 0.75 | NSM | F:protein binding | - |
| FL644055 | malate dehydrogenase | Symbiont | 0.75 | 24583394 | F:catalytic activity; P:generation of precursor metabolites and energy; P:catabolic process; P:metabolic process; P:carbohydrate metabolic process; F:nucleotide binding | EC:1.1.1.0 |
| FL644412 | regulator of g-protein signaling 10-like | Symbiont | 0.75 | NSM |  | - |
| FL644277 | Hypothetical protein (*Trichomonas vaginalis* G3) | Symbiont | 0.75 | NSM | P:vesicle-mediated transport; C:membrane |  |
| FL643045 | viral a-type inclusion protein | Symbiont | 0.75 | NSM | F:ATP binding; F:nucleotide binding; F:microtubule motor activity; P:microtubule-based movement; C:microtubule |  |
| FL642809 | Hypothetical protein IMG5_091710 (*Ichthyophthirius multifiliis*) | Symbiont | 0.75 | NSM | - |  |
| FL644029 | charged multivesicular body protein 4b | Symbiont | 0.75 | 24658229 | C:plasma membrane; P:protein transport | - |
| FL642377, FL644007 | ribosomal protein l8 | Symbiont | 0.75 | 17864318 | C:intracellular; F:structural molecule activity; C:ribosome; P:translation | - |
| FL643941 | phosphoglycerate kinase | Symbiont | 0.75 | 17136394 | C:cytoplasm; F:kinase activity; P:metabolic process; P:carbohydrate metabolic process; P:generation of precursor metabolites and energy; P:catabolic process; F:nucleotide binding; P:biosynthetic process | EC:2.7.2.3 |
| FL645049 | agc family protein kinase | Symbiont | 0.75 | NSM | F:kinase activity; F:ATP binding; F:protein kinase activity; P:phosphorylation; P:protein amino acid phosphorylation; F:zinc ion binding; P:protein ubiquitination; F:ubiquitin-protein ligase activity |  |
| FL643080 | dockerin type 1 | Symbiont | 0.75 | NSM | F:hydrolase activity, hydrolyzing O-glycosyl compounds; P:carbohydrate metabolic process; P:polysaccharide catabolic process |  |
| FL643474, FL643482 | Hypothetical protein (*Trichomonas vaginalis* G3) | Symbiont | 0.75 | NSM | - |  |
| FL635556 | transmembrane 9 superfamily protein member | Host | 0.74 | NSM | C:cell | - |
| FL644683 | chromosome segregation atpase-like protein | Symbiont | 0.74 | NSM | P:oxidation reduction; F:oxidoreductase activity; F:hydrolase activity; P:peptidyl-tyrosine dephosphorylation; F:protein tyrosine phosphatase activity; F:2-alkenal reductase activity; C:cytoplasm; C:cytosol; C:Golgi apparatus; F:calcium ion binding; P:intracellular protein transport; C:intracellular; C:spindle; C:nucleus; C:mitochondrion; C:microtubule; C:microtubule organizing center; P:cellular response to peptide hormone stimulus; C:plasma membrane; C:cytoskeleton |  |
| FL641290, FL642667 | NSM | Symbiont | 0.74 | NSM | - |  |
| FL643288, FL645697 | peroxidase ppod1 | Symbiont | 0.74 | NSM | F:actin binding | - |
| FL645388 | Hypothetical protein (*Trichomonas vaginalis* G3) | Symbiont | 0.74 | NSM | F:binding |  |
| FL643049 | psp-related protein | Symbiont | 0.74 | NSM | - |  |
| FL645353 | glycosyl hydrolase family7 | Symbiont | 0.74 | NSM | F:hydrolase activity; P:carbohydrate metabolic process | EC:3.2.1.0 |
| FL635444 | Hypothetical protein BRAFLDRAFT_87654 (*Branchiostoma floridae*) | Host | 0.74 | NSM | - |  |
| FL642416, FL643639 | succinate- ligase | Symbiont | 0.74 | 21356231 | F:catalytic activity; C:organelle; C:cytoplasm; F:nucleotide binding; P:generation of precursor metabolites and energy; P:catabolic process; C:protein complex | EC:6.2.1.4 |
| FL637840 | cytochrome p450 | Host | 0.74 | 17647305 | F:binding; F:catalytic activity; P:metabolic process; F:electron carrier activity | - |
| FL645330 | mannan endo- -beta-mannosidase | Symbiont | 0.74 | NSM | P:carbohydrate metabolic process; F:binding; F:hydrolase activity | EC:3.2.1.4 |
| FL642422 | predicted protein (*Nematostella vectensis*) | Symbiont | 0.74 | NSM | - |  |
| FL638095 | small GTP-binding protein (*Trichomonas vaginalis*) | Host | 0.74 | 17137088 | F:nucleotide binding; P:signal transduction; C:intracellular; P:protein transport; F:hydrolase activity; P:nucleobase, nucleoside, nucleotide and nucleic acid metabolic process; P:catabolic process; C:cell | - |
| FL645652 | enolase | Symbiont | 0.73 | 24580914 | C:cell; C:cytosol; C:protein complex; F:catalytic activity; F:binding; P:carbohydrate metabolic process; P:generation of precursor metabolites and energy; P:catabolic process; P:biosynthetic process; P:cellular amino acid and derivative metabolic process | EC:4.2.1.11 |
| FL638256 | leucine rich repeat family protein | Host | 0.73 | NSM | - |  |
| FL642519 | tpr domain containing protein | Symbiont | 0.73 | NSM | F:binding |  |
| FL642048 | protein yipf5-like | Symbiont | 0.73 | NSM | C:membrane |  |
| FL643273 | NSM | Symbiont | 0.73 | NSM | - |  |
| FL644136 | aminotransferase, classes I and II family protein(*Trichomonas vaginalis* G3) | Symbiont | 0.73 | 24580972 | F:transferase activity; P:cellular amino acid and derivative metabolic process; P:biosynthetic process; F:binding | EC:2.6.1.0 |
| FL644429 | pwp1 homolog (*S. cerevisiae*) | Symbiont | 0.73 | NSM | F:protein binding | - |
| FL642833, FL645405 | ribosomal protein l10a | Symbiont | 0.73 | 24662946 | C:nucleolus; F:structural molecule activity; C:plasma membrane; P:multicellular organismal development; C:ribosome; C:cytosol; P:translation; P:biological_process; F:RNA binding | - |
| FL641023, FL643056 | flagellar associated protein | Symbiont | 0.73 | NSM | C:flagellum; F:calcium ion binding; C:membrane |  |
| FL643947 | Hypothetical protein (*Trichomonas vaginalis* G3) | Symbiont | 0.73 | NSM | - |  |
| FL644792 | succinyl-diaminopimelate desuccinylase | Symbiont | 0.73 | NSM | F:hydrolase activity | - |
| FL643640 | Hypothetical protein (*Trichomonas vaginalis* G3) | Symbiont | 0.73 | NSM | - |  |
| FL643139 | NSM | Symbiont | 0.73 | NSM | - |  |
| FL643151 | Hypothetical protein (*Trichomonas vaginalis* G3) | Symbiont | 0.73 | NSM | - |  |
| FL644591 | sel1 repeat-containing protein | Symbiont | 0.73 | 21355295 | F:protein binding | - |
| FL641163 | muscle-specific protein 20-like | Symbiont | 0.72 | NSM | F:protein binding | - |
| FL641628, FL644428 | dynein heavy chain axonemal | Symbiont | 0.72 | 221458016 | F:motor activity; C:cytoskeleton; C:protein complex; P:biological_process; P:metabolic process | - |
| FL635318 | NSM | Host | 0.72 | NSM | - |  |
| FL644613 | nad-dependent epimerase dehydratase family protein | Symbiont | 0.72 | 24649454 | F:catalytic activity; P:metabolic process; F:binding; F:nucleotide binding | - |
| FL644643 | ankyrin repeat protein | Symbiont | 0.72 | 161082089 | F:catalytic activity; F:protein binding | - |
| FL643768 | CoA-substrate-specific enzyme activase (*Treponema brennaborense* DSM 12168) | Symbiont | 0.72 | NSM | - |  |
| FL644353 | actin-related protein 2 3 complex subunit 4 | Symbiont | 0.72 | 20129261 | C:cytoskeleton; C:protein complex; F:protein binding; F:actin binding; P:cytoskeleton organization | - |
| FL641149 | phospholipase c-like 2 | Symbiont | 0.72 | NSM | F:protein binding | - |
| FL645167 | ribosomal protein l18 | Symbiont | 0.72 | 21357009 | P:embryonic development; P:multicellular organismal development; C:ribosome; F:structural molecule activity; P:biological_process; P:reproduction; P:growth; P:transport; P:cellular component organization; P:translation | - |
| FL641816 | rab gdp dissociation inhibitor alpha | Symbiont | 0.72 | 24583049 | P:biological_process; F:catalytic activity; P:protein transport; F:enzyme regulator activity | - |
| FL642946, FL645126 | 40s ribosomal protein s16 | Symbiont | 0.72 | 19922746 | P:cytoskeleton organization; P:cell cycle; F:structural molecule activity; C:lipid particle; C:ribosome; C:cytosol; P:translation; P:biological_process | - |
| FL643117, FL643127 | 60s ribosomal protein l8 | Symbiont | 0.72 | 17864318 | C:ribosome; F:structural molecule activity; P:translation; P:biological_process | - |
| FL644005 | Hypothetical protein (*Trichomonas vaginalis* G3) | Symbiont | 0.72 | NSM | - |  |
| FL642745 | ribosomal protein l29 | Symbiont | 0.72 | 24639917 | F:structural molecule activity; C:ribosome; P:translation | - |
| FL645747 | NSM | Symbiont | 0.72 | NSM | - |  |
| FL645628 | leucine-rich repeat-containing protein 56-like | Symbiont | 0.71 | 24667178 | F:protein binding | - |
| FL642223 | Hypothetical protein Shel_04070 (*Slackia heliotrinireducens* DSM 20476) | Symbiont | 0.71 | NSM | F:protein binding | - |
| FL641366 | 60s ribosomal protein l26- | Symbiont | 0.71 | 21357853 | F:structural molecule activity; P:translation; C:ribosome | - |
| FL642239, FL642831, FL645272, FL645325 | me1 protein |  | 0.71 | 6634090 | C:cytosol; F:nucleotide binding; P:transcription; P:regulation of biological process; P:response to endogenous stimulus; C:cell; C:mitochondrion; F:binding; C:nucleoplasm; C:protein complex; P:metabolic process; F:DNA binding; F:catalytic activity; P:cellular component organization; F:protein binding; P:generation of precursor metabolites and energy; P:catabolic process | EC:1.1.1.40; EC:1.1.1.38 |
| FL643134 | Hypothetical protein (*Trichomonas vaginalis* G3) | Symbiont | 0.71 | NSM | - |  |
| FL641254 | NSM | Symbiont | 0.71 | NSM | F:protein binding | - |
| FL644169 | sel1 repeat protein | Symbiont | 0.71 | NSM | F:protein binding | - |
| FL641410, FL643051, FL644288, FL645720 | adhesin protein ap33-1 | Symbiont | 0.71 | 28574296 | F:catalytic activity; C:organelle; C:cytoplasm; F:nucleotide binding; F:transferase activity; C:protein complex; P:generation of precursor metabolites and energy; P:catabolic process; F:binding | EC:6.2.1.4; EC:2.3.3.8; EC:6.2.1.5 |
| FL642787 | triosephosphate isomerase | Symbiont | 0.71 | 28572006 | P:biosynthetic process; P:carbohydrate metabolic process; F:catalytic activity | EC:5.3.1.1 |
| FL642708 | NSM | Symbiont | 0.71 | NSM | - |  |
| FL644565 | 60s ribosomal protein l10 | Symbiont | 0.71 | 221513692 | F:structural molecule activity; C:ribosome; P:translation | - |
| FL642354 | malate dehydrogenase | Symbiont | 0.71 | 24583394 | F:catalytic activity; C:cytosol; P:generation of precursor metabolites and energy; P:catabolic process; P:metabolic process; P:carbohydrate metabolic process; F:nucleotide binding | EC:1.1.1.0 |
| FL642483 | solute carrier family 25 member 42 | Symbiont | 0.71 | NSM | C:mitochondrion; C:cell; F:binding; P:transport | - |
| FL643966 | Hypothetical protein (*Trichomonas vaginalis* G3) | Symbiont | 0.71 | NSM | - |  |
| FL635753 | cytochrome p450 | Host | 0.71 | 24652913 | C:cell | - |
| FL642527 | vacuolar protein sorting protein | Symbiont | 0.71 | NSM | F:protein binding; F:binding | - |
| FL641052 | predicted protein (*Nematostella vectensis*) | Symbiont | 0.71 | NSM | - |  |
| FL645138 | ribosomal protein s14 | Symbiont | 0.71 | 17975579 | C:ribosome; F:structural molecule activity; P:translation; P:biological_process | - |
| FL643487 | ser thr protein phosphatase | Symbiont | 0.70 | 17864424 | F:phosphoprotein phosphatase activity; P:protein modification process | EC:3.1.3.16 |
| FL642334 | heat shock protein | Symbiont | 0.70 | 17647529 | P:protein metabolic process; P:response to stress; P:response to biotic stimulus; C:microtubule organizing center; F:nucleotide binding; F:protein binding; F:hydrolase activity; P:response to abiotic stimulus | - |
| FL643823 | Predicted-protein: TAR1-like (*Glycine max*) | Symbiont | 0.70 | NSM | C:mitochondrion; F:molecular_function; P:regulation of cellular respiration |  |
| FL644735 | 60s acidic ribosomal protein p1 | Symbiont | 0.70 | 17136320 | C:ribosome; F:structural molecule activity; P:translation; P:biological_process | - |
| FL645489 | NSM | Symbiont | 0.70 | NSM | - |  |
| FL642371 | glycosyl hydrolase family7 | Symbiont | 0.70 | NSM | F:hydrolase activity; P:carbohydrate metabolic process | EC:3.2.1.0 |
| FL644472 | NSM | Symbiont | 0.70 | NSM | - |  |
| FL643928 | 40s ribosomal protein s25 | Symbiont | 0.70 | NSM | C:ribosome |  |
| FL644713 | mannan endo- -beta-mannosidase | Symbiont | 0.70 | NSM | P:carbohydrate metabolic process; F:binding; F:hydrolase activity | EC:3.2.1.4 |
| FL641022, FL643868 | 60s ribosomal protein l7 | Symbiont | 0.70 | 24583248 | C:nucleolus; C:plasma membrane; F:protein binding; C:cell wall; C:vacuole; C:ribosome; C:cytosol; C:plastid | - |
| FL643939 | NSM | Symbiont | 0.70 | NSM | - |  |
| FL641211 | NSM | Symbiont | 0.70 | NSM | - |  |
| FL645362 | surface antigen -like | Symbiont | 0.70 | NSM | - |  |
| FL644787, FL645013 | serine threonine-protein kinase nek1 | Symbiont | 0.70 | 21358073 | F:nucleotide binding; P:protein modification process; F:protein kinase activity; F:protein binding; P:cellular amino acid and derivative metabolic process | EC:2.7.11.0 |
| FL644440 | e3 ubiquitin-protein ligase fancl | Symbiont | 0.70 | NSM | F:zinc ion binding; F:metal ion binding; P:biological_process; C:cellular_component |  |
| FL645235 | px domain containing protein | Symbiont | 0.70 | NSM | F:protein binding; P:cell communication; F:lipid binding | - |
| FL644869 | protein fam91a1-like | Symbiont | 0.69 | 21356633 | - |  |
| FL643103 | mob1 phocein family protein | Symbiont | 0.69 | 28571820 | C:cytoplasm; P:protein modification process; F:enzyme regulator activity; P:metabolic process; P:regulation of biological process; C:nucleus; F:protein binding | - |
| FL643792 | Hypothetical protein (*Trichomonas vaginalis* G3) | Symbiont | 0.69 | NSM | C:cell | - |
| FL645152 | 40s ribosomal protein s8 | Symbiont | 0.69 | 24651181 | F:structural molecule activity; C:ribosome; C:cytosol; P:translation; P:nucleobase, nucleoside, nucleotide and nucleic acid metabolic process | - |
| FL643287 | ribosomal protein l17 | Symbiont | 0.69 | 18921137 | P:cytoskeleton organization; P:cell cycle; C:ribosome | - |
| FL643923 | prefoldin subunit 3 | Symbiont | 0.69 | 24645921 | P:protein metabolic process; C:cytosol; C:protein complex; F:protein binding | - |
| FL644908 | Hypothetical protein (*Trichomonas vaginalis* G3) | Symbiont | 0.69 | NSM | P:transcription initiation from RNA polymerase II promoter; F:RNA polymerase II transcription factor activity; C:transcription factor TFIIA complex |  |
| FL643048, FL644154 | NSM | Symbiont | 0.69 | NSM | - |  |
| FL645075 | 3-beta-hydroxysteroid-delta -isomerase | Symbiont | 0.69 | NSM | P:biosynthetic process; P:lipid metabolic process; C:endoplasmic reticulum; C:cell; F:catalytic activity | EC:5.3.3.5 |
| FL643426, FL643644 | clathrin and vps domain-containing protein | Symbiont | 0.69 | 17137000 | P:anatomical structure morphogenesis; C:cytoplasmic membrane-bounded vesicle; F:signal transducer activity; F:cytoskeletal protein binding; P:ion transport; P:protein transport; C:Golgi apparatus; F:protein binding; C:cell; P:organelle organization; C:cytoskeleton; P:transport; P:cellular component organization; P:metabolic process; P:cell cycle; P:signal transduction; F:binding; C:plasma membrane; C:protein complex; C:cytoplasm; P:regulation of biological process; C:mitochondrion | - |
| FL645265 | NSM | Symbiont | 0.69 | NSM | - |  |
| FL645312 | NSM | Symbiont | 0.69 | NSM | - |  |
| FL642700 | organophosphate pesticide hydrolase | Symbiont | 0.69 | NSM | F:hydrolase activity | - |
| FL644972 | Hypothetical protein (*Trichomonas vaginalis* G3) | Symbiont | 0.69 | NSM | - |  |
| FL641015, FL643186 | succinyl- beta subunit | Symbiont | 0.69 | 21356231 | F:catalytic activity; C:organelle; C:cytoplasm; F:nucleotide binding; P:generation of precursor metabolites and energy; P:catabolic process; C:protein complex | EC:6.2.1.4 |
| FL644981 | dna mismatch repair protein | Symbiont | 0.69 | 24664545 | F:nucleotide binding; P:response to stress; P:DNA metabolic process; F:DNA binding | - |
| FL643430 | clathrin heavy chain | Symbiont | 0.69 | 17137000 | P:anatomical structure morphogenesis; F:signal transducer activity; C:Golgi apparatus; F:protein binding; P:transport; P:cellular component organization; P:protein transport; C:cell; C:cytoskeleton; C:plasma membrane; P:organelle organization; P:cell cycle; P:signal transduction; C:cytoplasmic membrane-bounded vesicle; C:protein complex; C:cytoplasm; P:regulation of biological process; C:mitochondrion | - |
| FL643121 | pecanex-like protein 1-like | Symbiont | 0.68 | NSM | C:integral to membrane |  |
| FL642793 | pctp-like protein | Symbiont | 0.68 | NSM | - |  |
| FL641435 | Hypothetical protein (*Tuber melanosporum* Mel28) | Symbiont | 0.68 | NSM | F:hydrolase activity, hydrolyzing O-glycosyl compounds; F:cellulose binding; P:carbohydrate metabolic process; C:extracellular region |  |
| FL642388 | rna (guanine-9-)-methyltransferase domain-containing protein 2-like | Symbiont | 0.68 | NSM | F:methyltransferase activity; F:transferase activity; P:methylation; F:molecular_function; P:biological_process; C:cellular_component |  |
| FL644823 | udp-glucose 6-dehydrogenase | Symbiont | 0.68 | 2114495 | F:catalytic activity; P:metabolic process; F:nucleotide binding | EC:1.1.1.0 |
| FL643401 | beta-tubulin | Symbiont | 0.68 | 24655746 | C:cytosol; P:cytoskeleton organization; P:cell cycle; C:cytoskeleton; C:protein complex; F:receptor binding; F:protein binding; P:cellular component organization; P:nucleobase, nucleoside, nucleotide and nucleic acid metabolic process; P:catabolic process; P:biological_process; F:hydrolase activity; F:structural molecule activity; F:nucleotide binding; P:signal transduction; C:plasma membrane | - |
| FL641034 | Hypothetical protein (*Trichomonas vaginalis*) | Symbiont | 0.68 | NSM | - |  |
| FL645364 | ubiquitin-conjugating enzyme e2 n | Symbiont | 0.68 | 17530929 | P:protein modification process; P:regulation of biological process; P:organelle organization; P:response to stress; P:DNA metabolic process; F:protein binding; C:cytosol; P:protein metabolic process; P:catabolic process; C:intracellular; C:protein complex; P:signal transduction; F:catalytic activity; P:biological_process; P:transcription; C:nucleus | EC:6.3.2.19 |
| FL641394 | heat shock protein 90 | Symbiont | 0.68 | 17647529 | P:protein metabolic process; C:plastid; P:response to stress; F:nucleotide binding; F:protein binding | - |
| FL642497 | ibr domain containing protein | Symbiont | 0.68 | NSM | F:protein binding; F:binding | - |
| FL642320 | 40s ribosomal protein s11 | Symbiont | 0.68 | 24652924 | F:structural molecule activity; C:ribosome; C:cytosol; P:translation; P:biological_process | - |
| FL641109, FL642604, FL643416 | phosphoenolpyruvate carboxykinase | Symbiont | 0.68 | 24655085 | C:cytoplasm; P:biosynthetic process; P:carbohydrate metabolic process; F:binding; F:nucleotide binding; F:kinase activity; P:metabolic process; F:catalytic activity; P:generation of precursor metabolites and energy; P:catabolic process | EC:4.1.1.32 |
| FL644142 | 60s ribosomal protein l11 | Symbiont | 0.68 | 17137026 | C:ribosome; F:structural molecule activity; P:translation; P:biological_process | - |
| FL642136 | transcription factor 2b | Symbiont | 0.67 | 19921082 | C:nucleus; P:transcription; P:regulation of biological process; P:multicellular organismal development; F:transcription factor activity; P:nucleobase, nucleoside, nucleotide and nucleic acid metabolic process; C:organelle; C:intracellular; P:anatomical structure morphogenesis; P:cellular component organization; P:cell differentiation; C:nucleoplasm; C:protein complex | - |
| FL644224 | Hypothetical protein (*Trichomonas vaginalis* G3) | Symbiont | 0.67 | NSM | - |  |
| FL643955 | NSM | Symbiont | 0.67 | NSM | P:biological_process; F:binding; P:response to stress; P:response to abiotic stimulus | - |
| FL641185 | dynein heavy chain axonemal-like | Symbiont | 0.67 | 320545435 | F:nucleotide binding; F:motor activity; P:biological_process; C:cytoskeleton; C:protein complex | - |
| FL641050, FL641187 | viral a-type inclusion protein | Symbiont | 0.67 | NSM | - |  |
| FL645023 | ef hand family protein | Symbiont | 0.67 | 17647231 | F:calcium ion binding | - |
| FL642968 | Hypothetical protein HMPREF1073_03039 (*Bacteroides uniformis* CL03T12C37) | Symbiont | 0.67 | NSM | - |  |
| FL642720 | NSM | Symbiont | 0.67 | NSM | - |  |
| FL644266 | NSM | Symbiont | 0.67 | NSM | - |  |
| FL645310 | rab gdp dissociation inhibitor alpha | Symbiont | 0.67 | 24583049 | P:protein transport; F:enzyme regulator activity | - |
| FL645472 | Predicted protein (*Naegleria gruberi*) | Symbiont | 0.67 | NSM | C:protein complex; C:plasma membrane; F:ion channel activity; P:ion transport; F:binding | - |
| FL643213 | Hypothetical protein TTHERM_01326860 (*Tetrahymena thermophila*) | Symbiont | 0.67 | NSM | - |  |
| FL643485 | serine threonine-protein kinase pk-1 (stopk-1) | Symbiont | 0.67 | 24658719 | F:protein kinase activity; F:nucleotide binding; P:protein modification process | - |
| FL641532 | NSM | Symbiont | 0.67 | NSM | - |  |
| FL644919, FL645501 | NSM | Host | 0.66 | NSM | - |  |
| FL642423 | clan family unassigned serine peptidase | Symbiont | 0.66 | NSM | P:protein metabolic process; P:catabolic process; F:peptidase activity | - |
| FL645409 | Hypothetical protein (*Trichomonas vaginalis* G3) | Symbiont | 0.66 | NSM | F:small GTPase regulator activity |  |
| FL641476 | pyruvate phosphate dikinase | Symbiont | 0.66 | NSM | F:catalytic activity; P:metabolic process | - |
| FL645336 | leucine rich repeat family | Symbiont | 0.66 | NSM | F:GTP binding |  |
| FL642983 | 40s ribosomal protein s24 | Symbiont | 0.66 | 20130247 | F:structural molecule activity; C:ribosome; P:translation; F:nucleotide binding | - |
| FL637315, FL641207 | Hypothetical protein (*Trichomonas vaginalis* G3) | Mixed | 0.66 | NSM | - |  |
| FL643714 | Conserved Hypothetical Protein (*Enterococcus faecalis* HH22) | Symbiont | 0.66 | NSM | - |  |
| FL644954, FL645084 | Predicted protein (*Nematostella vectensis*) | Symbiont | 0.66 | NSM | - |  |
| FL642161 | dynein light chain | Symbiont | 0.66 | 17137630 | P:regulation of biological process; P:growth; P:biosynthetic process; P:DNA metabolic process; P:reproduction; P:multicellular organismal development; P:anatomical structure morphogenesis; F:protein binding; P:cell proliferation; P:biological_process; P:transport; P:cellular component organization; P:embryonic development; P:cell cycle; C:cytoskeleton; C:protein complex | - |
| FL644888 | cytoplasmic heat shock protein 70 | Symbiont | 0.66 | 17737967 | F:nucleotide binding; P:response to stress | - |
| FL644097 | flagellar outer dynein arm heavy chain gamma | Symbiont | 0.66 | 219131049 | C:cytoskeleton; C:protein complex; P:metabolic process; C:organelle; C:intracellular; F:motor activity; P:biological_process | - |
| FL643402, FL645016 | clan family cathepsin l-like cysteine peptidase | Symbiont | 0.66 | 320543907 | P:protein metabolic process; P:catabolic process; F:peptidase activity | - |
| FL644480 | Hypothetical protein (*Trichomonas vaginalis* G3) | Symbiont | 0.66 | NSM | - |  |
| FL645309 | nadh dehydrogenase | Symbiont | 0.66 | 19921784 | F:catalytic activity; C:protein complex; C:mitochondrion; P:multicellular organismal development; P:generation of precursor metabolites and energy; P:biosynthetic process; P:ion transport; F:nucleotide binding | EC:1.6.5.3 |
| FL644966 | exo-beta- -glucanase exg0 | Symbiont | 0.65 | NSM | F:hydrolase activity | - |
| FL642617 | glycogen phosphorylase | Symbiont | 0.65 | 24581010 | F:transferase activity; P:carbohydrate metabolic process | EC:2.4.1.1 |
| FL642758 | tpr domain containing protein | Symbiont | 0.65 | NSM | F:binding |  |
| FL645581, FL645587, FL645594 | camk family protein kinase | Symbiont | 0.65 | 8356 | P:biological_process; F:protein kinase activity; F:protein binding; P:anatomical structure morphogenesis; P:cellular component organization; C:nucleus; C:microtubule organizing center | - |
| FL644464 | NSM | Symbiont | 0.65 | NSM | - |  |
| FL642593 | ghf3 protein | Symbiont | 0.65 | NSM | F:hydrolase activity; P:carbohydrate metabolic process | EC:3.2.1.0 |
| FL642925 | NSM | Symbiont | 0.65 | NSM | F:binding | - |
| FL643743 | NSM | Symbiont | 0.65 | NSM | - |  |
| FL643207 | dna replication factor a subunit ssb3 | Symbiont | 0.65 | NSM | C:heterotrimeric G-protein complex; F:signal transducer activity; C:membrane; P:signal transduction; P:G-protein coupled receptor protein signaling pathway; P:DNA replication; F:single-stranded DNA binding; C:cytosol; C:DNA replication factor A complex; P:telomere maintenance; C:nucleus |  |
| FL642307 | xylose isomerase domain-containing protein tim barrel | Symbiont | 0.65 | NSM | F:isomerase activity; F:DNA binding |  |
| FL644653 | NSM | Symbiont | 0.65 | NSM | - |  |
| FL645439 | guanine nucleotide-binding protein subunit beta-2-like 1 | Symbiont | 0.65 | 17137396 | P:regulation of biological process; P:metabolic process; P:cellular component organization; P:protein metabolic process; F:protein binding; C:intracellular | - |
| FL643411 | iq calmodulin-binding motif family protein | Symbiont | 0.65 | NSM | F:protein binding | - |
| FL643293 | NSM | Symbiont | 0.65 | NSM | - |  |
| FL643731 | Hypothetical Protein AthiA1_03397 (*Acidithiobacillus thiooxidans* ATCC 19377) | Symbiont | 0.64 | NSM | - |  |
| FL642051, FL644218, FL644973, FL645182, FL645369 | ef hand family protein | Symbiont | 0.64 | NSM | F:calcium ion binding | - |
| FL641311 | FYVE zinc finger family protein (*Trichomonas vaginalis* G3) | Symbiont | 0.64 | NSM | F:enzyme regulator activity; C:intracellular; P:signal transduction | - |
| FL644650 | xanthine dehydrogenase | Symbiont | 0.64 | 8831 | F:catalytic activity; P:metabolic process; F:nucleotide binding | - |
| FL643511 | ef hand family protein | Symbiont | 0.64 | NSM | - |  |
| FL642568 | chromosome segregation | Symbiont | 0.64 | NSM | C:chromosome; F:ATP binding; P:chromosome organization; F:protein serine/threonine kinase activity; P:positive regulation of centrosome duplication; P:protein amino acid phosphorylation; F:nucleotide binding; P:neural tube closure; P:signal transduction; C:spindle pole centrosome; C:intracellular; P:actin cytoskeleton organization; F:actin binding; F:phospholipid binding; P:mitotic chromosome condensation; C:nucleus; P:mitosis; P:cell division; P:chromosome condensation; C:condensin complex; F:protein heterodimerization activity; P:cell cycle; F:exonuclease activity |  |
| FL645458 | enolase | Symbiont | 0.64 | 17137654 | C:cell; C:cytosol; C:protein complex; F:catalytic activity; F:binding; P:carbohydrate metabolic process; P:generation of precursor metabolites and energy; P:catabolic process; P:biosynthetic process; P:cellular amino acid and derivative metabolic process | EC:4.2.1.11 |
| FL644157 | xanthine dehydrogenase | Symbiont | 0.64 | 8831 | F:nucleotide binding; F:catalytic activity; F:electron carrier activity; F:binding; P:metabolic process | - |
| FL642191 | NSM | Symbiont | 0.64 | NSM | - |  |
| FL645109 | kelch motif family protein | Symbiont | 0.63 | NSM | F:enzyme regulator activity; C:intracellular; P:signal transduction | - |
| FL641434 | icc family phosphohydrolase | Symbiont | 0.63 | NSM | F:hydrolase activity | - |
| FL645678 | Hypothetical protein (*Trichomonas vaginalis* G3) | Symbiont | 0.63 | NSM | F:transferase activity; P:nucleobase, nucleoside, nucleotide and nucleic acid metabolic process | EC:2.7.7.50 |
| FL641451 | protein kinase domain containing protein | Symbiont | 0.63 | NSM | F:protein kinase activity; F:nucleotide binding; P:protein modification process | - |
| FL644818 | serine/threonine-protein kinase mph1 (*Schizosaccharomyces japonicus* yFS275) | Symbiont | 0.63 | 24647737 | C:cytosol; F:protein kinase activity; P:regulation of biological process; P:organelle organization; P:cell cycle; P:biological_process; C:nucleus; P:cellular amino acid and derivative metabolic process; F:nucleotide binding; P:protein modification process | EC:2.7.11.0 |
| FL642123 | viral a-type inclusion protein | Symbiont | 0.63 | NSM | P:regulation of transcription, DNA-dependent; P:transcription, DNA-dependent; F:sequence-specific DNA binding; F:protein dimerization activity; F:transcription factor activity; C:nucleus |  |
| FL645733 | ankyrin repeat | Symbiont | 0.63 | 161082089 | F:protein binding | - |
| FL642980, FL643616, FL643838 | ef hand family protein | Symbiont | 0.63 | NSM | F:calcium ion binding | - |
| FL643875, FL644444 | ubiquitin c | Symbiont | 0.63 | 24640086 | P:viral reproduction; P:transport; P:cell cycle; P:signal transduction; P:response to stress; P:DNA metabolic process; C:cytosol; C:nucleoplasm; P:protein metabolic process; P:catabolic process; P:regulation of biological process; P:cell death; C:plasma membrane; P:transcription; F:protein binding; C:endosome; P:cellular component organization; C:cytoplasmic membrane-bounded vesicle; P:nucleobase, nucleoside, nucleotide and nucleic acid metabolic process | - |
| FL643172 | clan family cathepsin l-like cysteine peptidase | Symbiont | 0.63 | NSM | P:protein metabolic process; P:catabolic process; F:peptidase activity | - |
| FL645323 | peroxidase PPOD1 (*Naegleria gruberi*) | Symbiont | 0.62 | NSM | F:calcium ion binding; F:actin filament binding; F:protein binding, bridging; F:peroxidase activity; F:hydrolase activity |  |
| FL644814 | Hypothetical protein (*Trichomonas vaginalis* G3) | Symbiont | 0.62 | NSM | - |  |
| FL643389 | ribosomal protein l24e | Symbiont | 0.62 | 19921254 | F:structural constituent of ribosome; P:translation; C:ribosome |  |
| FL642862 | undecaprenyl diphosphate synthase | Symbiont | 0.62 | 18857969 | F:transferase activity | EC:2.5.1.0 |
| FL645394 | NSM | Symbiont | 0.62 | NSM | - |  |
| FL642734 | NSM | Symbiont | 0.62 | NSM | - |  |
| FL644051 | clan family metacaspase-like cysteine peptidase | Symbiont | 0.62 | NSM | F:peptidase activity; P:protein metabolic process; P:catabolic process | EC:3.4.22.0 |
| FL645401 | NSM | Symbiont | 0.61 | NSM | - |  |
| FL641971 | Hypothetical protein (*Trichomonas vaginalis* G3) | Symbiont | 0.61 | NSM | - |  |
| FL645538 | Family T1, proteasome beta subunit, threonine peptidase (*Trichomonas vaginalis* G3) | Symbiont | 0.61 | 21355629 | F:peptidase activity; C:intracellular; C:protein complex; P:protein metabolic process; P:catabolic process | EC:3.4.25.0 |
| FL645604 | coiled-coil domain containing 19 | Symbiont | 0.61 | NSM | F:molecular_function; P:biological_process; C:cellular_component; C:mitochondrion; C:soluble fraction; C:flagellum |  |
| FL644916 | trna binding domain containing protein | Symbiont | 0.61 | 21357223 | F:binding | - |
| FL639043 | NSM | Host | 0.61 | NSM | - |  |
| FL644389 | proteasome subunit alpha type 2 | Symbiont | 0.61 | 17737927 | C:protein complex; C:intracellular; P:protein metabolic process; P:catabolic process; F:peptidase activity | EC:3.4.25.0 |
| FL641086, FL641514, FL641529, FL641666, FL641891, FL641972, FL642427, FL643611, FL644186, FL644334, FL644958, FL645406, FL645332, FL645648 | alpha tubulin 1a | Symbiont | 0.61 | 17136564 | P:protein metabolic process; C:cytosol; P:cell cycle; P:cellular component organization; C:cytoskeleton; C:protein complex; P:nucleobase, nucleoside, nucleotide and nucleic acid metabolic process; P:catabolic process; P:biological_process; F:protein binding; F:hydrolase activity; F:structural molecule activity; F:nucleotide binding | - |
| FL642041, FL643519 | alpha-tubulin 1 | Symbiont | 0.61 | 17136564 | C:cytosol; P:cellular component organization; C:cytoskeleton; C:protein complex; P:nucleobase, nucleoside, nucleotide and nucleic acid metabolic process; P:catabolic process; P:biological_process; F:protein binding; F:hydrolase activity; F:structural molecule activity; F:nucleotide binding | - |
| FL645382 | leucine-rich repeat | Symbiont | 0.61 | NSM | P:anatomical structure morphogenesis; P:multicellular organismal development; P:biological_process; P:reproduction; P:behavior; F:protein binding; P:regulation of biological process; P:response to abiotic stimulus; C:cytoplasm; P:cytoskeleton organization; P:embryonic development | - |
| FL642513 | Hypothetical protein (*Trichomonas vaginalis* G3) | Symbiont | 0.61 | NSM | - |  |
| FL641996 | NSM | Symbiont | 0.60 | NSM | - |  |
| FL639840, FL644753 | NSM | Mixed | 0.60 | NSM | - |  |
| FL644943 | pf08719 domain protein | Symbiont | 0.60 | NSM | - |  |
| FL644087 | NSM | Symbiont | 0.60 | NSM | - |  |
| FL643208 | Iron only hydrogenase large subunit, C-terminal domain containing protein (*Trichomonas vaginalis* G3) | Symbiont | 0.60 | NSM | F:binding; F:catalytic activity | - |
| FL644956 | tpr repeat protein | Symbiont | 0.60 | NSM | F:protein binding | - |
| FL641384 | hydroxymethylglutaryl- synthase | Symbiont | 0.60 | 17933694 | P:biosynthetic process; P:lipid metabolic process; F:transferase activity; P:metabolic process; P:catabolic process; P:cellular amino acid and derivative metabolic process | EC:2.3.3.10 |
| FL644408 | NSM | Symbiont | 0.60 | NSM | - |  |
| FL644794 | 200 kda antigen p200 | Symbiont | 0.60 | NSM | - |  |
| FL641316, FL641726, FL642438, FL643645, FL643673, FL643781, FL644829, FL644647, FL644674, FL645032, FL645725, FL645455 | alpha tubulin 1a | Symbiont | 0.60 | 17136564 | P:protein metabolic process; C:cytosol; P:cell cycle; P:cellular component organization; C:cytoskeleton; C:protein complex; P:nucleobase, nucleoside, nucleotide and nucleic acid metabolic process; P:catabolic process; P:biological_process; F:protein binding; F:hydrolase activity; F:structural molecule activity; F:nucleotide binding |  |
| FL643385 | agc family protein kinase | Symbiont | 0.59 | 24643817 | P:anatomical structure morphogenesis; P:cellular component organization; P:cell differentiation; P:multicellular organismal development; C:nucleoplasm; P:regulation of biological process; P:cell death; P:signal transduction; P:response to stress; F:enzyme regulator activity; C:cytosol; F:protein kinase activity; P:cell-cell signaling; P:cellular amino acid and derivative metabolic process; F:nucleotide binding; P:protein modification process | EC:2.7.11.0 |
| FL642516 | sel1 domain protein repeat-containing protein | Symbiont | 0.59 | NSM | F:protein binding | - |
| FL641166 | linker histone h1 and h5 family protein | Symbiont | 0.59 | NSM | C:nucleosome; P:nucleosome assembly; C:nucleus; F:DNA binding |  |
| FL644856 | Hypothetical protein (*Trichomonas vaginalis* G3) | Symbiont | 0.58 | NSM | - |  |
| FL644630 | ankyrin repeat protein | Symbiont | 0.58 | 161082089 | F:protein binding | - |
| FL643974, FL644936, FL645181 | dynein heavy chain | Symbiont | 0.58 | 6644386 | F:motor activity; C:cilium; C:organelle; C:intracellular; C:cytoskeleton; C:protein complex; P:nucleobase, nucleoside, nucleotide and nucleic acid metabolic process; P:catabolic process; P:reproduction; F:nucleotide binding; P:biological_process; F:hydrolase activity | EC:3.6.1.3 |
| FL641300 | pyruvate ferredoxin flavodoxin oxidoreductase | Symbiont | 0.58 | NSM | F:binding; F:catalytic activity; P:metabolic process | - |
| FL643238 | Hypothetical protein (*Trichomonas vaginalis* G3) | Symbiont | 0.58 | NSM | - |  |
| FL641209 | Hypothetical protein (*Trichomonas vaginalis*) | Symbiont | 0.57 | NSM | - |  |
| FL643697 | xyppx repeat family protein | Symbiont | 0.57 | NSM | C:integral to membrane; C:membrane; F:molecular_function; P:biological_process; C:cellular_component |  |
| FL642059, FL644213, FL645137 | dynein heavy chain axonemal | Symbiont | 0.57 | 386769771 | F:motor activity; C:cytoskeleton; C:protein complex; P:nucleobase, nucleoside, nucleotide and nucleic acid metabolic process; P:catabolic process; F:nucleotide binding; P:biological_process; F:hydrolase activity | EC:3.6.1.3 |
| FL645455, FL641786, FL642213, FL643632 | beta tubulin | Symbiont | 0.57 | 158739 | C:cytoskeleton; C:protein complex; P:cellular component organization; P:nucleobase, nucleoside, nucleotide and nucleic acid metabolic process; P:catabolic process; P:biological_process; F:hydrolase activity; F:structural molecule activity; F:nucleotide binding | - |
| FL643896 | nudix family protein | Symbiont | 0.57 | NSM | F:hydrolase activity; C:plastid | - |
| FL645558 | beta-tubulin | Symbiont | 0.57 | 24655741 | C:cytoskeleton; C:protein complex; P:cellular component organization; P:nucleobase, nucleoside, nucleotide and nucleic acid metabolic process; P:catabolic process; P:biological_process; F:hydrolase activity; F:structural molecule activity; C:cytoplasm; F:nucleotide binding; C:nucleus | - |
| FL643404 | ef hand family protein | Symbiont | 0.57 | NSM | F:calcium ion binding | - |
| FL641089, FL641525, FL644174 | pyruvate:ferredoxin homodimeric | Symbiont | 0.57 | NSM | F:binding; F:catalytic activity; P:metabolic process | - |
| FL641107, FL641711, FL641566 | beta tubulin | Symbiont | 0.56 | 158739 | C:cytoskeleton; C:protein complex; P:cellular component organization; P:nucleobase, nucleoside, nucleotide and nucleic acid metabolic process; P:catabolic process; P:biological_process; F:hydrolase activity; F:structural molecule activity; F:nucleotide binding | - |
| FL644425 | endoplasmic reticulum heat shock 70 kda protein | Symbiont | 0.56 | 24641402 | C:cell; C:endoplasmic reticulum; C:protein complex; F:protein binding; F:enzyme regulator activity; P:signal transduction; P:anatomical structure morphogenesis; P:multicellular organismal development; P:regulation of biological process; P:embryonic development; P:nucleobase, nucleoside, nucleotide and nucleic acid metabolic process; P:catabolic process; P:response to external stimulus; P:response to stress; P:protein modification process; P:protein metabolic process; P:cell death; P:response to biotic stimulus; C:cytosol; P:transport; C:cytoplasm; F:binding; P:cell communication; F:nucleotide binding; F:hydrolase activity; C:cytoplasmic membrane-bounded vesicle; P:metabolic process; F:calcium ion binding; C:nucleus; C:extracellular region | EC:3.6.1.3 |
| FL641030, FL641317, FL641347, FL641425, FL641486, FL642148, FL642156, FL642649, FL642690, FL643055, FL642882, FL644391, FL644864, FL645271 | beta tubulin | Symbiont | 0.56 | 158739 | C:cytoskeleton; C:protein complex; P:cellular component organization; P:nucleobase, nucleoside, nucleotide and nucleic acid metabolic process; P:catabolic process; P:biological_process; F:hydrolase activity; F:structural molecule activity; F:nucleotide binding |  |
| FL641330 | Hypothetical protein (*Trichomonas vaginalis* G3) | Symbiont | 0.56 | NSM | F:binding |  |
| FL644366 | radial spoke head protein 4 homolog a | Symbiont | 0.56 | 20130335 | C:cell; C:intracellular | - |
| FL644230 | dnak protein | Symbiont | 0.56 | NSM | F:ATP binding; F:nucleotide binding |  |
| FL645215 | adhesin-like protein | Symbiont | 0.56 | NSM | C:extrachromosomal circular DNA |  |
| FL644371 | Hypothetical protein (*Trichomonas vaginalis* G3) | Symbiont | 0.56 | NSM | F:binding |  |
| FL642298 | surface antigen -like | Symbiont | 0.55 | NSM | - |  |
| FL641831, FL645399, FL644182, FL644655 | beta tubulin | Symbiont | 0.55 | 158739 | C:cytoskeleton; C:protein complex; P:cellular component organization; P:nucleobase, nucleoside, nucleotide and nucleic acid metabolic process; P:catabolic process; P:biological_process; F:hydrolase activity; F:structural molecule activity; F:nucleotide binding | - |
| FL643143 | Hypothetical protein (*Trichomonas vaginalis* G3) | Symbiont | 0.55 | NSM | - |  |
| FL643897 | NSM | Symbiont | 0.55 | NSM | - |  |
| FL645408 | surface antigen -like | Symbiont | 0.55 | NSM | F:hydrolase activity, hydrolyzing O-glycosyl compounds; P:carbohydrate metabolic process; P:polysaccharide catabolic process; F:GTP binding |  |
| FL644365 | plasmodium exported protein | Symbiont | 0.55 | NSM | C:cell wall; C:cell surface; C:membrane; P:phagocytosis; C:extracellular region; P:pathogenesis; P:xylan catabolic process; F:chitin binding; P:chitin metabolic process |  |
| FL641310 | beta tubulin | Symbiont | 0.55 | 24655737 | C:cytoskeleton; C:protein complex; P:cellular component organization; P:nucleobase, nucleoside, nucleotide and nucleic acid metabolic process; P:catabolic process; P:biological_process; F:hydrolase activity; F:structural molecule activity; F:nucleotide binding | - |
| FL642038, FL644900 | isoleucyl-trna synthetase | Symbiont | 0.55 | 24668543 | F:catalytic activity; C:cytoplasm; C:nucleus; P:translation; P:nucleobase, nucleoside, nucleotide and nucleic acid metabolic process; P:cellular amino acid and derivative metabolic process; F:nucleotide binding | - |
| FL643435 | tyrosyl-trna synthetase | Symbiont | 0.55 | NSM | F:catalytic activity; P:translation; P:nucleobase, nucleoside, nucleotide and nucleic acid metabolic process; P:cellular amino acid and derivative metabolic process; F:nucleotide binding; C:cytoplasm | - |
| FL642689 | psp-related protein | Symbiont | 0.55 | NSM | - |  |
| FL643899 | pepsinogen c | Symbiont | 0.55 | 17986011 | F:peptidase activity; P:protein metabolic process; P:catabolic process | EC:3.4.23.0 |
| FL644772 | Hypothetical protein (*Trichomonas vaginalis* G3) | Symbiont | 0.55 | NSM | - |  |
| FL641018 | Hypothetical protein (*Trichomonas vaginalis* G3) | Symbiont | 0.54 | NSM | - |  |
| FL644436 | dna replication licensing factor mcm7 | Symbiont | 0.54 | 17647617 | P:cell cycle; P:regulation of biological process; F:hydrolase activity; P:metabolic process; C:nucleoplasm; C:nuclear chromosome; C:protein complex; F:DNA binding; C:chromosome; P:DNA metabolic process; P:biosynthetic process; F:protein binding; P:response to stress; F:nucleotide binding | - |
| FL645381 | kelch motif family protein | Symbiont | 0.54 | NSM | F:enzyme regulator activity; C:intracellular; P:signal transduction | - |
| FL645190 | nudix family protein | Symbiont | 0.54 | NSM | F:hydrolase activity; C:plastid | - |
| FL641388 | low quality protein: protein nlrc3-like | Symbiont | 0.53 | NSM | P:negative regulation of NF-kappaB transcription factor activity; F:ATP binding; F:molecular_function; C:cytoplasm; F:nucleotide binding; P:I-kappaB kinase/NF-kappaB cascade; P:T cell activation |  |
| FL643377, FL643694, FL644892 | beta tubulin | Symbiont | 0.53 | 158739 | C:cytoskeleton; C:protein complex; P:cellular component organization; P:nucleobase, nucleoside, nucleotide and nucleic acid metabolic process; P:catabolic process; P:biological_process; F:hydrolase activity; F:structural molecule activity; F:nucleotide binding | - |
| FL645508 | surface antigen -like | Symbiont | 0.53 | NSM | F:unfolded protein binding; F:hydrolase activity, hydrolyzing O-glycosyl compounds; P:protein folding; P:polysaccharide catabolic process; F:heat shock protein binding |  |
| FL641072 | serine protease inhibitor-like spi-1 protein | Symbiont | 0.53 | NSM | F:peptidase activity |  |
| FL643070 | NSM | Symbiont | 0.53 | NSM | - |  |
| FL643940 | Hypothetical protein (*Trichomonas vaginalis* G3) | Symbiont | 0.53 | NSM | - |  |
| FL642565 | NSM | Symbiont | 0.53 | NSM | - |  |
| FL644279 | Hypothetical protein (*Trichomonas vaginalis* G3) | Symbiont | 0.53 | NSM | - |  |
| FL645024 | Hypothetical protein (*Trichomonas vaginalis* G3) | Symbiont | 0.53 | NSM | P:nucleobase, nucleoside, nucleotide and nucleic acid metabolic process; F:ATP binding; F:nucleobase, nucleoside, nucleotide kinase activity; F:binding; P:metabolic process; F:catalytic activity |  |
| FL642909 | glycylpeptide n-tetradecanoyltransferase 2 | Symbiont | 0.52 | 17647739 | F:transferase activity; P:biosynthetic process; P:protein modification process | EC:2.3.1.97 |
| FL644639 | hydrogenosomal fe-hydrogenase | Symbiont | 0.52 | NSM | F:binding; F:catalytic activity; P:metabolic process; C:intracellular; C:protein complex; F:electron carrier activity | EC:1.12.7.2 |
| FL643568 | cytochrome b5 domain-containing protein 1-like | Symbiont | 0.52 | NSM | F:binding | - |
| FL644580 | luminal binding protein | Symbiont | 0.51 | 24641402 | P:protein metabolic process; P:response to stress; P:response to abiotic stimulus; C:endoplasmic reticulum; P:reproduction; P:anatomical structure morphogenesis; P:cellular component organization; P:cell differentiation; P:multicellular organismal development; P:cell growth; F:nucleotide binding | - |
| FL641141 | camk family protein kinase | Symbiont | 0.51 | 21356537 | F:protein kinase activity; F:nucleotide binding; P:protein modification process | - |
| FL643746 | NSM | Symbiont | 0.50 | NSM | - |  |
| FL641105 | Hypothetical protein (*Trichomonas vaginalis* G3) | Symbiont | 0.50 | NSM | - |  |
| FL643556 | zinc mynd-type containing 12 | Symbiont | 0.50 | NSM | F:binding; C:intracellular | - |
| FL643503 | adhesin-like protein | Symbiont | 0.49 | NSM | F:peptidase activity | - |
| FL644799 | alpha-tubulin 1 | Symbiont | 0.48 | 17136564 | C:cytosol; P:cellular component organization; C:cytoskeleton; C:protein complex; P:nucleobase, nucleoside, nucleotide and nucleic acid metabolic process; P:catabolic process; P:biological_process; F:protein binding; F:hydrolase activity; F:structural molecule activity; F:nucleotide binding | - |
| FL642874 | NSM | Symbiont | 0.48 | NSM | - |  |
| FL641184 | adenylate kinase 7 | Symbiont | 0.48 | NSM | F:nucleotide binding; P:nucleobase, nucleoside, nucleotide and nucleic acid metabolic process; F:kinase activity | - |
| FL645093 | major facilitator superfamily transporter | Symbiont | 0.48 | 24652789 | P:response to stress; P:response to abiotic stimulus; P:transport; C:vacuole; F:transporter activity; P:response to endogenous stimulus; C:plastid; C:cell | - |
| FL644017 | NSM | Symbiont | 0.48 | NSM | - |  |
| FL643756 | Hypothetical protein (*Trichomonas vaginalis* G3) | Symbiont | 0.47 | NSM | - |  |
| FL643307 | glucose-6-phosphate isomerase | Symbiont | 0.47 | 17737445 | C:cytoplasm; P:biosynthetic process; P:carbohydrate metabolic process; F:catalytic activity; P:generation of precursor metabolites and energy; P:catabolic process; P:nucleobase, nucleoside, nucleotide and nucleic acid metabolic process; P:secondary metabolic process | EC:5.3.1.9 |
| FL642348 | gcn5-related n-acetyltransferase | Symbiont | 0.47 | NSM | F:transferase activity | - |
| FL643422 | sel1 domain protein repeat-containing protein | Symbiont | 0.47 | NSM | F:protein binding | - |
| FL644517 | heat shock protein 90 | Symbiont | 0.46 | 17647529 | P:protein metabolic process; C:cytoplasm; F:protein binding; F:nucleotide binding; P:response to stress | - |
| FL643902 | Hypothetical protein (*Trichomonas vaginalis* G3) | Symbiont | 0.46 | NSM | - |  |
| FL644285 | heat shock protein 83-1 | Symbiont | 0.46 | 17647529 | P:protein metabolic process; P:response to stress; P:response to biotic stimulus; C:microtubule organizing center; F:nucleotide binding; F:protein binding; F:hydrolase activity; P:response to abiotic stimulus | - |
| FL639242 | NSM | Host | 0.46 | NSM | - |  |
| FL643489 | leucine-rich repeat-containing protein 48 | Symbiont | 0.45 | NSM | C:cytoplasm | - |
| FL643268 | tcp-1 cpn60 chaperonin family protein | Symbiont | 0.45 | 24649027 | P:protein metabolic process; C:cytoplasm; F:protein binding; F:nucleotide binding | - |
| FL643390 | NSM | Symbiont | 0.44 | NSM | - |  |
| FL645067 | ef hand family protein | Symbiont | 0.43 | NSM | C:cell; F:calcium ion binding | - |
| FL642860 | camk family protein kinase | Symbiont | 0.43 | NSM | F:kinase activity; F:ATP binding; F:protein kinase activity; P:phosphorylation; F:nucleotide binding; P:protein amino acid phosphorylation; F:protein serine/threonine kinase activity |  |
| FL642605 | heat shock protein | Symbiont | 0.43 | 17647529 | P:protein metabolic process; C:cytoplasm; F:protein binding; F:nucleotide binding; P:response to stress | - |
| FL643526 | NSM | Symbiont | 0.42 | NSM | - |  |
| FL645627 | NSM | Symbiont | 0.40 | NSM | - |  |
| FL641526 | 6-phosphofructokinase | Symbiont | 0.40 | 28573326 | P:carbohydrate metabolic process; P:metabolic process; F:kinase activity; C:cytosol; C:protein complex; F:nucleotide binding; P:generation of precursor metabolites and energy; P:catabolic process; P:biosynthetic process; P:nucleobase, nucleoside, nucleotide and nucleic acid metabolic process; P:secondary metabolic process | EC:2.7.1.11 |
| FL641533, FL643251 | radial spoke protein 11 | Symbiont | 0.39 | NSM | P:signal transduction; F:enzyme regulator activity | - |
| FL642512 | transcription factor | Symbiont | 0.38 | 24583511 | C:nucleus; P:transcription; P:regulation of biological process; P:multicellular organismal development; F:transcription factor activity; P:nucleobase, nucleoside, nucleotide and nucleic acid metabolic process; C:organelle; C:intracellular; P:anatomical structure morphogenesis; P:cellular component organization; P:cell differentiation; C:nucleoplasm; C:protein complex | - |
| FL642764 | ef hand family protein | Symbiont | 0.35 | NSM | F:calcium ion binding | - |
| FL643652 | cysteine synthase a | Symbiont | 0.32 | 313103969 | F:binding; F:catalytic activity; P:biosynthetic process; P:cellular amino acid and derivative metabolic process; F:transferase activity; P:metabolic process; C:protein complex; C:cytoplasm | EC:2.5.1.47 |

Table S1C. Fold enrichment values of JH upregulated ESTs of host origin.

| **S1C**  **GO Term** | **GO ID** | **Fold Enrichment** | ***p*-Value** |
| --- | --- | --- | --- |
| Ferrous Iron Binding | GO:0008198 | 170.28 | 0.01 |
| Ferric Iron Binding | GO:0008199 | 85.14 | 0.02 |
| Kinase Binding | GO:0019900 | 34.06 | 0.06 |
| Structural Constituent of Chitin-based Cuticle | GO:0005214 | 6.97 | 0.06 |
| Structural Constituent of Cuticle | GO:0042302 | 6.39 | 0.08 |
| Iron Ion Binding | GO:0005506 | 3.93 | 0.07 |
| Structural Molecule Activity | GO:0005198 | 3.36 | 0.03 |
| Peptidase Activity | GO:0008233 | 2.76 | 0.03 |
| Peptidase Activity, Acting on L-amino Acid Peptides | GO:0070011 | 2.50 | 0.08 |

Table S1D. Fold enrichment values of JH downregulated ESTs of host origin.

| **S1D**  **GO Term** | **GO ID** | **Fold Enrichment** | | ***p*-Value** | |
| --- | --- | --- | --- | --- | --- |
| Tetrapyrrole Binding | GO:0046906 | 14.14 | 0.02 | |  |
| Heme Binding | GO:0020037 | 14.14 | 0.02 | |  |
| Electron Carrier Activity | GO:0009055 | 9.80 | 0.03 | |  |
| Iron Ion Binding | GO:0005506 | 7.61 | 0.05 | |  |
| Serine-type Endopeptidase Activity | GO:0004252 | 7.07 | 0.06 | |  |
| Serine-type Peptidase Activity | GO:0008236 | 6.43 | 0.07 | |  |
| Serine Hydrolase Activity | GO:0017171 | 6.39 | 0.07 | |  |
| Endopeptidase Activity | GO:0004175 | 5.78 | 0.02 | |  |
| Peptidase Activity, Acting on L-amino Acid Peptides | GO:0070011 | 4.31 | 0.05 | |  |
| Peptidase Activity | GO:0008233 | 4.07 | 0.05 | |  |

Table S1E. Fold enrichment values of JH downregulated ESTs of symbiont origin.

| **S1E**  **GO Term** | **GO ID** | **Fold Enrichment** | ***p*-Value** |
| --- | --- | --- | --- |
| Ribosomal Protein S6 Kinase Activity | GO:0004711 | 102.83 | 0.02 |
| Succinate-CoA Ligase (GDP-forming) Activity | GO:0004776 | 51.42 | 0.04 |
| Transferase Activity, Transferring Acyl Groups, Acyl Groups Converted into Alkyl on Transfer | GO:0046912 | 29.38 | 0.07 |
| Intramolecular Oxidoreductase Activity, Interconverting Aldoses and Ketoses | GO:0016861 | 29.38 | 0.07 |
| Succinate-CoA Ligase Activity | GO:0004774 | 29.38 | 0.07 |
| rRNA Binding | GO:0019843 | 17.88 | 0.00 |
| Translation Elongation Factor Activity | GO:0003746 | 15.42 | 0.02 |
| Structural Constituent of Ribosome | GO:0003735 | 9.82 | 0.00 |
| Structural Constituent of Cytoskeleton | GO:0005200 | 7.35 | 0.06 |
| SUMO Binding | GO:0032183 | 6.33 | 0.02 |
| Small Conjugating Protein Binding | GO:0032182 | 6.23 | 0.03 |
| Unfolded Protein Binding | GO:0051082 | 5.48 | 0.04 |
| Structural Molecule Activity | GO:0005198 | 4.96 | 0.00 |
| GTPase Activity | GO:0003924 | 4.75 | 0.01 |
| GTP Binding | GO:0005525 | 4.62 | 0.00 |
| Guanyl ribonucleotide Binding | GO:0032561 | 4.55 | 0.00 |
| Guanyl Nucleotide Binding | GO:0019001 | 4.52 | 0.00 |
| Purine Ribonucleotide Binding | GO:0032555 | 2.90 | 0.00 |
| Ribonucleotide Binding | GO:0032553 | 2.90 | 0.00 |
| Purine Nucleotide Binding | GO:0017076 | 2.70 | 0.00 |
| ATP Binding | GO:0005524 | 2.47 | 0.00 |
| Nucleotide Binding | GO:0000166 | 2.47 | 0.00 |
| Adenyl Ribonucleotide Binding | GO:0032559 | 2.47 | 0.00 |
| Adenyl Nucleotide Binding | GO:0030554 | 2.28 | 0.00 |
| Purine Nucleoside Binding | GO:0001883 | 2.27 | 0.00 |
| Nucleoside Binding | GO:0001882 | 2.25 | 0.00 |

Table S2A. SHE up-regulated transcripts (Fold change ratio >1.18, with P<0.05). (NSM= No significant matches). Individual sequences and contigs are listed according to fold change. For contigs, average fold change values are provided. The putative sequence identities, gene ontology (GO) and enzyme codes were obtained from BLAST2GO. *Drosophila melanogaster* homologs were obtained by BLASTX. Enzyme codes were not found for any of the sequences.

| **S2A**  **Accession #** | **Putative Sequence Identity** | **Origin** | **Fold Change** | ***Drosophila  melanogaster*  Homolog** | **GOs** | **Enzyme codes** |
| --- | --- | --- | --- | --- | --- | --- |
| FL636972 | NSM | Host | 2.038 | NSM | - |  |
| FL643723 | NSM | Symbiont | 1.273 | NSM | - |  |
| FL640552 | pro-resilin | Host | 1.201 | 24654243 | F:structural constituent of cuticle |  |

Table S2B. SHE down-regulated transcripts (Fold change ratio <0.84, with P<0.05). (NSM= No significant matches). Individual sequences and contigs are listed according to fold change. For contigs, average fold change values are provided. The putative sequence identities, gene ontology (GO) and enzyme codes were obtained from BLAST2GO. *Drosophila melanogaster* homologs were obtained by BLASTX. Enzyme codes were not found for any of the sequences.

| **S2B**  **Accession #** | **Putative Sequence Identity** | **Origin** | **Fold Change** | ***Drosophila melanogaster* Homolog** | **GOs** | **Enzyme codes** |
| --- | --- | --- | --- | --- | --- | --- |
| FL642199 | gtp-binding protein | Symbiont | 0.835 | 24581168 | F:GTP binding; F:GTPase activity; P:GTP catabolic process; F:nucleotide binding |  |
| FL644436 | dna replication licensing factor mcm7 | Symbiont | 0.811 | 17647617 | P:DNA replication; F:ATP binding; P:DNA replication initiation; F:nucleotide binding; F:nucleoside-triphosphatase activity; C:nucleus; F:DNA binding; P:DNA unwinding involved in replication; F:hydrolase activity; P:cell proliferation; P:regulation of phosphorylation; C:MCM complex; P:response to DNA damage stimulus; P:cell cycle checkpoint; P:M/G1 transition of mitotic cell cycle; P:mitotic cell cycle; P:DNA strand elongation involved in DNA replication; C:chromatin; F:ATP-dependent DNA helicase activity; P:S phase of mitotic cell cycle; P:G1/S transition of mitotic cell cycle; F:single-stranded DNA binding; C:nucleoplasm; F:protein binding; F:DNA helicase activity; P:response to drug; P:cellular response to epidermal growth factor stimulus; P:regulation of transcription, DNA-dependent; F:DNA-dependent ATPase activity; P:cellular response to organic substance; C:cytosol |  |
| FL644818 | serine/threonine-protein kinase mph1 (*Schizosaccharomyces japonicus* yFS275) | Symbiont | 0.733 | 24647737 | F:ATP binding; F:protein kinase activity; P:protein amino acid phosphorylation; F:protein serine/threonine kinase activity; F:kinase activity; P:phosphorylation; F:nucleotide binding; F:transferase activity; P:cellular protein localization; P:mitosis; C:nucleus; P:mitotic cell cycle spindle assembly checkpoint; P:cell division; C:cytosol; P:cell cycle |  |

Table S3A. LR up-regulated transcripts (Fold change ratio >1.18, with P<0.05). (NSM= No significant matches). Individual sequences and contigs are listed according to fold change. For contigs, average fold change values are provided. The putative sequence identities, gene ontology (GO) and enzyme codes were obtained from BLAST2GO. *Drosophila melanogaster* homologs were obtained by BLASTX.

| **S3A**  **Accession #** | **Putative  Sequence Identity** | **Origin** | **Fold Change** | ***Drosophila  melanogaster*  homolog** | **GOs** | **Enzyme Codes** |
| --- | --- | --- | --- | --- | --- | --- |
| FL636990 | NSM | Host | 2.17 | NSM | - |  |
| FL637910, FL639730 | serine protease 13 | Host | 2.14 | 24658993 | F:peptidase activity | - |
| FL635003 | Hypothetical protein EAG_11594 (*Camponotus floridanus*) | Host | 1.52 | NSM | C:membrane; P:ion transport |  |
| FL637565 | alpha-amylase | Host | 1.45 | 17137384 | F:hydrolase activity; F:binding; P:carbohydrate metabolic process | EC:3.2.1.1 |
| FL643467 | diacylglycerol kinase (*Plasmodium vivax* Sal-1) | Symbiont | 1.31 | NSM | - |  |
| FL643545 | NSM | Symbiont | 1.26 | NSM | - |  |
| FL636931 | Hypothetical protein TcasGA2_TC011099 (*Tribolium castaneum)* | Host | 1.22 | 24654487 | C:organelle; C:intracellular | - |
| FL643000 | NSM | Symbiont | 1.21 | NSM | - |  |
| FL637796 | NSM | Host | 1.20 | NSM | - |  |

Table S3B. LR down-regulated transcripts (Fold change ratio <0.84, with P<0.05). (NSM= No significant matches). Individual sequences and contigs are listed according to fold change. For contigs, average fold change values are provided. The putative sequence identities, gene ontology (GO) and enzyme codes were obtained from BLAST2GO. *Drosophila melanogaster* homologs were obtained by BLASTX. Enzyme codes were not found for any of the sequences.

| **S3B**  **Accession #** | **Putative Sequence Identity** | **Origin** | **Fold Change** | ***Drosophila melanogaster* homolog** | **GOs** | **Enzyme Codes** |
| --- | --- | --- | --- | --- | --- | --- |
| FL645211 | NSM | Symbiont | 0.83 | NSM | - |  |
| FL644365 | plasmodium exported protein | Symbiont | 0.83 | NSM | C:cell wall; C:cell surface; C:membrane; P:phagocytosis; C:extracellular region; P:pathogenesis; F:chitin binding; P:chitin metabolic process; P:xylan catabolic process |  |
| FL641166 | linker histone h1 and h5 family protein | Symbiont | 0.63 | NSM | C:nucleosome; P:nucleosome assembly; C:nucleus; F:DNA binding |  |

Table S4A. LS up-regulated transcripts (Fold change ratio >1.18, with P<0.05). (NSM= No significant matches). Individual sequences and contigs are listed according to fold change. For contigs, average fold change values are provided. The putative sequence identities, gene ontology (GO) and enzyme codes were obtained from BLAST2GO. *Drosophila melanogaster* homologs were obtained by BLASTX.

| **S4A**  **Accession #** | **Putative Sequence Identity** | **Origin** | **Fold change** | ***Drosophila melanogaster* Homolog** | **GOs** | **Enzyme Codes** |
| --- | --- | --- | --- | --- | --- | --- |
| FL641666 | alpha-tubulin 1 | Symbiont | 1.21 | 17136564 | P:protein metabolic process; C:cytoskeleton; C:protein complex; P:cellular component organization; P:cytoskeleton organization; P:nucleobase, nucleoside, nucleotide and nucleic acid metabolic process; P:catabolic process; P:biological_process; F:protein binding; F:hydrolase activity; F:structural molecule activity; C:cytoplasm; F:nucleotide binding | - |
| FL637563, FL637681, FL638273, FL638189, FL638500, FL639734, FL635306, FL636517, FL636689 | lysozyme ii | Host | 1.45 | 17136658 | F:hydrolase activity | - |
| FL636041 | high affinity choline transporter | Host | 1.22 | 21356865 | C:cell; P:ion transport; F:transporter activity; C:plasma membrane; P:biosynthetic process; P:cell-cell signaling; P:cellular amino acid and derivative metabolic process | - |
| FL635011, FL637865, FL636990 | c-type lectin precursor | Host | 1.49 | 28574695 | F:carbohydrate binding | - |
| FL639382 | alpha amylase | Host | 2.35 | 34808817 | F:hydrolase activity; F:binding; P:carbohydrate metabolic process | EC:3.2.1.1 |
| FL638734 | alpha-amylase | Host | 1.95 | 34808817 | F:hydrolase activity; F:binding; P:carbohydrate metabolic process | EC:3.2.1.1 |
| FL638301 | arylsulfatase b | Host | 1.31 | 281363223 | F:catalytic activity | - |
| FL642515 | ankyrin repeat protein | Symbiont | 1.24 | NSM | F:metal ion binding; F:zinc ion binding |  |
| FL644447 | Hypothetical protein BRAFLDRAFT_218619 (*Branchiostoma floridae*) | Symbiont | 1.34 | NSM | F:ATPase activity, coupled to transmembrane movement of substances; C:integral to membrane; P:transport; P:ATP catabolic process; F:nucleotide binding; F:nucleoside-triphosphatase activity; F:ATP binding; F:ATPase activity; P:transmembrane transport; F:transferase activity |  |
| FL642952, FL644473 | Hypothetical protein BRAFLDRAFT_218619 (*Branchiostoma floridae*) | Symbiont | 1.34 | NSM | F:binding | - |
| FL641250 | Hypothetical protein BRAFLDRAFT_218619 (*Branchiostoma floridae*) | Symbiont | 1.33 | NSM | F:ATPase activity, coupled to transmembrane movement of substances; C:integral to membrane; P:transport; P:ATP catabolic process; F:nucleotide binding; F:nucleoside-triphosphatase activity; F:ATP binding; F:ATPase activity; P:transmembrane transport |  |
| FL642545, FL644677 | Hypothetical protein BRAFLDRAFT_218619 (*Branchiostoma floridae*) | Symbiont | 1.33 | NSM | F:ATPase activity, coupled to transmembrane movement of substances; C:integral to membrane; P:transport; P:ATP catabolic process; F:nucleotide binding; F:nucleoside-triphosphatase activity; F:ATP binding; F:ATPase activity; P:transmembrane transport |  |
| FL639927 | lysozyme c-1 | Host | 1.42 | NSM | - |  |
| FL637519 | NSM | Host | 2.48 | NSM | - |  |
| FL639040 | NSM | Host | 2.00 | NSM | - |  |
| FL640824 | NSM | Host | 1.40 | NSM | - |  |
| FL641064 | NSM | Symbiont | 1.24 | NSM | - |  |
| FL637566 | NSM | Host | 1.24 | NSM | - |  |
| FL635544 | PREDICTED: venom allergen 3-like (*Megachile rotundata*) | Host | 2.67 | NSM | C:extracellular region |  |

Table S4B. LS down-regulated transcripts (Fold change ratio <0.84, with P<0.05). (NSM= No significant matches). Individual sequences and contigs are listed according to fold change. For contigs, average fold change values are provided. The putative sequence identities, gene ontology (GO) and enzyme codes were obtained from BLAST2GO. *Drosophila melanogaster* homologs were obtained by BLASTX. Enzyme codes were not found for any of the sequences.

| **S4B**  **Accession #** | **Putative Sequence Identity** | **Origin** | **Fold change** | ***Drosophila melanogaster* Homolog** | **GOs** | **Enzyme Codes** |
| --- | --- | --- | --- | --- | --- | --- |
| FL645673 | NSM | Symbiont | 0.84 | NSM | - |  |
| FL641569 | NSM | Symbiont | 0.82 | NSM | - |  |
| FL644587 | serine threonine-protein kinase | Symbiont | 0.82 | 227118 | F:protein kinase activity | - |
| FL641826 | actin | Symbiont | 0.80 | 17647133 | C:cytoskeleton; C:cytoplasm; F:nucleotide binding | - |
| FL645061 | NSM | Symbiont | 0.80 | NSM | - |  |
| FL636097 | NSM | Host | 0.76 | NSM | - |  |
