# Supplementary material for: Differential impacts of juvenile hormone, soldier head extract and alternate caste phenotypes on host and symbiont transcriptome composition in the gut of the termite Reticulitermes flavipes
Source: BMC Genomics. 2013 Jul 19;14:491. doi: 10.1186/1471-2164-14-491 (PMC3731027; doi:10.1186/1471-2164-14-491)

**Figure S1.** Peptide translation of the 50kDa midgut protein-encoding cDNA. The translation and alignment were obtained using the “Sixframe” tool at SDSC Biology Workbench (<http://workbench.sdsc.edu/>). Numbers at the left and right indicate nucleotide positions. The start and stop codons (atg and taa), polyadenylation signal (aataa) and poly-A tail (aaaaaa_n_) are underlined. Yellow shading indicates the predicted signal peptide and “?” its predicted cleavage site. Gray shading indicates amino acids predicted to be phosphorylated (17 total).

**Figure S2**. (A) Reverse phase C18 HPLC chromatogram showing the elution profiles and retention times for major components of soldier head extract. The SHE components ɣ-cadinenal (ALD) and ɣ-cadinene (CAD) and the internal standard 1-napthol are marked in the figure. (B) SHE-HPLC chromatogram from Tarver et al. (2011). The similarity of elution profiles in (A) and (B) demonstrates that SHE used in the present study is identical to that studies previously from other termite colonies.

(A)


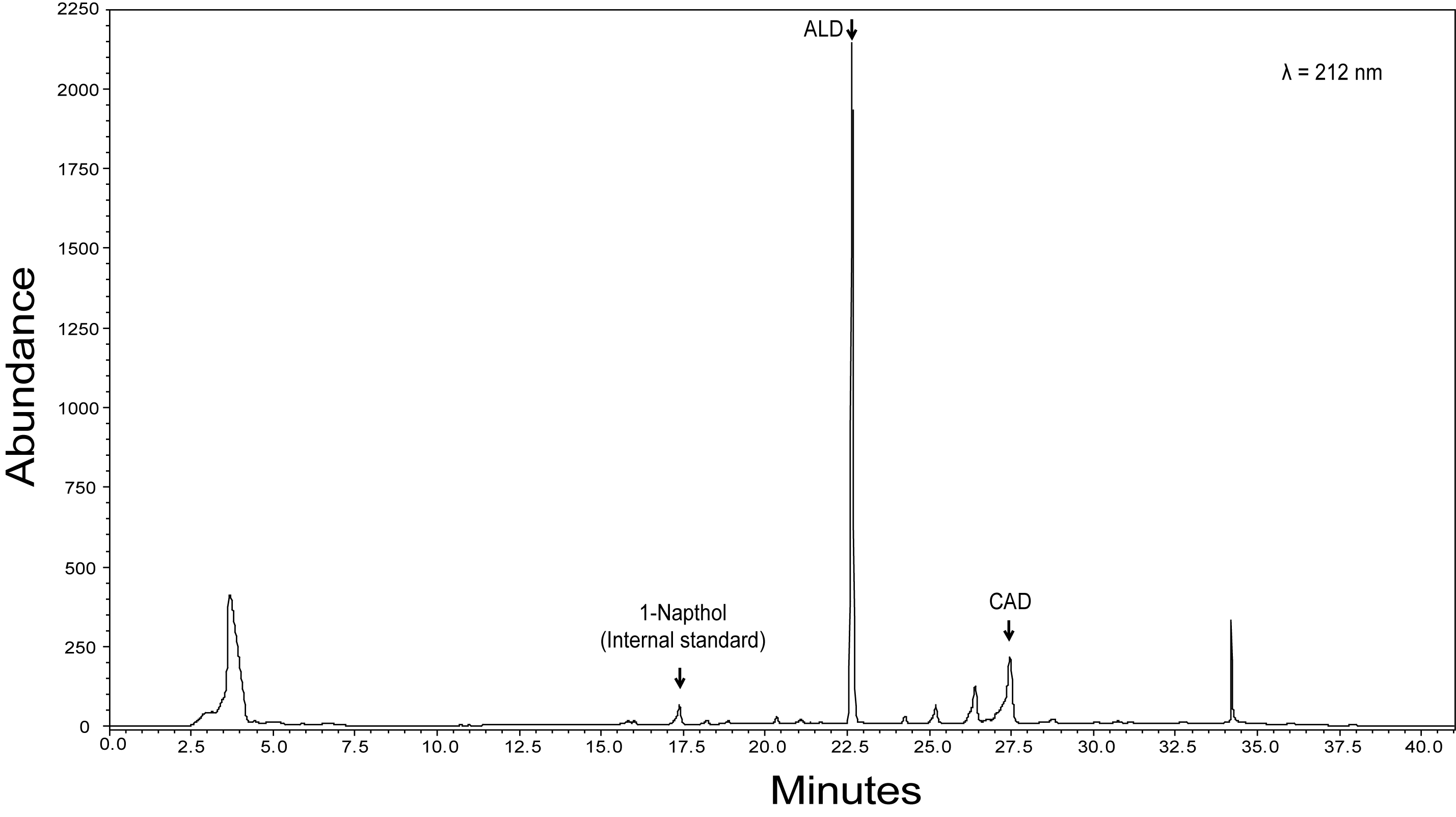


(B)


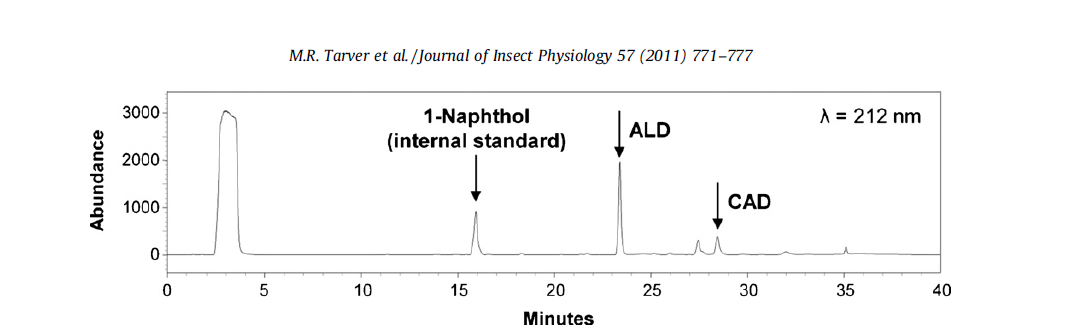

Supplement: Additional file 2 — 50 kDa midgut protein translation (Additional file 2: Figure S1) and HPLC analysis of soldier head extract (Additional file 2: Figure S2). [file 1471-2164-14-491-S2.docx]
