# Supplementary material for: Differential impacts of juvenile hormone, soldier head extract and alternate caste phenotypes on host and symbiont transcriptome composition in the gut of the termite Reticulitermes flavipes
Source: BMC Genomics. 2013 Jul 19;14:491. doi: 10.1186/1471-2164-14-491 (PMC3731027; doi:10.1186/1471-2164-14-491)
Supplement: Additional file 3 — Summary of ΔΔC T values for repeat bioassay qPCRs (Additional file3: Table S5) and the list of primers used for qPCR validations (Additional file3: Table S6). [file 1471-2164-14-491-S3.docx]

Table S5. ΔΔC_T_ values calculated from the cycle threshold value of qPCR with cDNA prepared from the RNA extracted from treatments with exposure to Live reproductive (LR) live soldiers (LS) and Soldier head extract (SHE). For primer details see Table S6.

| EST | Nature of expression | Fold change | ΔΔC_T_ values | | |
| --- | --- | --- | --- | --- | --- |
|  |  |  | SHE | LR | LS |
| TG_305_M8 | SHE up-regulated | 2.037821 | -0.188 |  |  |
| TS_42_D5 | SHE down-regulated | 0.811056 | 0.843 |  |  |
| TS_43_E6 | SHE down-regulated | 0.732792 | 1.019 |  |  |
| TG_00_B5 | LR up-regulated | 1.51658 |  | -2.076 |  |
| TG_02_C3 | LR up-regulated | 1.449932 |  | -0.758 |  |
| TG_305_F17 | LR up-regulated | 2.170865 |  | -2.902 |  |
| TG_03_E8 | LS up-regulated | 1.382973 |  |  | -1.943 |
| TG_07_D4 | LS up-regulated | 1.56197 |  |  | -0.890 |
| TG_09_A11 | LS up-regulated | 1.314489 |  |  | 3.174 |
| TG_17_H11 | LS up-regulated | 1.950465 |  |  | -0.034 |
| TG_23_E6 | LS up-regulated | 2.354824 |  |  | -1.428 |
| TG_309_G17 | LS up-regulated | 2.670969 |  |  | -3.686 |

Table S6. List of primers used for qPCR validation. Primer names starting with TG denote that they are from the host library and TS denote that they are from symbiont library. Successful primers could not be designed from the down-regulated ESTs of treatments with live reproductive and live soldiers.

| Treatment | Primer name | Original EST accn number | Primer sequence |
| --- | --- | --- | --- |
| JH up-regulated | PS_G05_F1 |  | CCGCCGTAGTTGCTAAAG |
|  | PS_G05_R1 |  | CAGCGTATCCCAGAGGAG |
|  | TG_01_G11_F1 | FL639806 | GCCGAAATTCCGATTACAAG |
|  | TG_01_G11_R1 |  | TGGAGTGACAGACCTACT |
|  | TG_03_D2_F1 | FL637656 | CACGCTCACAAGAACATCAG |
|  | TG_03_D2_R1 |  | CCCACACTTCCGAACACT |
|  | TG_03_D3_F1 | FL637666 | TGAGCTAGTCCCTTTCTAACC |
|  | TG_03_D3_R1 |  | ATGGCAAGAGATAACACAACAG |
|  | TG_07_B2_F1 | FL637824 | CCACTCTACTACTGGCTACTC |
|  | TG_07_B2_R1 |  | GAAACCGTCCGTTCTTCTC |
|  | TG_07_C9_F1 | FL637852 | CCTGGGAGAATGGAATGAAAT |
|  | TG_07_C9_R1 |  | CTGGTGAGAGGGTGAAATG |
|  | TG_09_B10_F1 | FL638311 | TTCAACCAGGACACCTATC |
|  | TG_09_B10_R1 |  | GGTATTCATCTCCGAAGAAACT |
|  | TG_13_B2_F1 | FL638494 | CGCTCTACTACTGGCTACTC |
|  | TG_13_B2_R1 |  | GAAACCGTCCGTTCTTCTC |
|  | TG_17_H11_F1 | FL638855 | ACCCGATCCCACCAAACTG |
|  | TG_17_H11_R1 |  | GTCGATGGAGGGTCTGTA |
|  | TG_23_C3_F1 | FL639359 | GCGTAACATCCCAGAAGAG |
|  | TG_23_C3_R1 |  | GCCGCCAAAGATCAGTAG |
|  | TG_29_C2_F1 | FL639931 | TTCATGCCTAGAGTTCTTCAC |
|  | TG_29_C2_R1 |  | GTAGTCGTATCACATTATCGTTTG |
|  | TG_302_D23_F1 | FL636096 | TGGCAATTACGCTCTTTCTG |
|  | TG_302_D23_R1 |  | CACACCTTGGTATGATCTTCA |
|  | TG_305_F13_F1 | FL636982 | GCACCTTGCGGTTAGTTAT |
|  | TG_305_F13_R1 |  | CTTTGCCTCTTGGTGTTC |
|  | TG_309_M9_F1 | FL635573 | TCGACACTCGACATACAGA |
|  | TG_309_M9_R1 |  | AGCCAGTCCACTTCCAAA |
|  | TG_33_E9_F1 | FL640421 | ACGAAGTACCTGGGAGAATG |
|  | TG_33_E9_R1 |  | CTGGTGAGAGGGTGAAATG |
|  | TG_33_H1_F1 | FL640448 | GGCATCAGGGTATTCACAA |
|  | TG_33_H1_R1 |  | TCACGATTCAGCTCTGGTAG |
|  | TG_36_E4_F1 | FL640694 | GCGTTGCAGTTTGGTTTC |
|  | TG_36_E4_R1 |  | TCGTGATGGTTCCATGTTC |
|  | TG_37_B8_F1 | FL640754 | CAGAAGTATAAGCAGCAGTAAGAG |
|  | TG_37_B8_R1 |  | GAGCATTGTGGTTGGTAGTC |
| JH down-regulated | TG_03_C7_F1 | FL637679 | TGACCACATCCTTAACACAAC |
|  | TG_03_C7_R1 |  | AGCCCGACACATAGAACATC |
|  | TG_03_D12_F1 | FL637679 | GGCTTGCTGTTGGTCTTAC |
|  | TG_03_D12_R1 |  | GCTGCTGGATAGCCATTTACT |
|  | TG_14_C6_F1 | FL638604 | CACAGCGACAAGCCTTATG |
|  | TG_14_C6_R1 |  | AGCTTCAGGTCACGGATCAA |
|  | TG_19_C10_F1 | FL639066 | GTGCAGTGAGGCACAATAAG |
|  | TG_19_C10_R1 |  | AAGACACTCAGCCAGAAGA |
|  | TG_20_B8_F1 | FL639157 | ACATCAGCATCGCCAACTAC |
|  | TG_20_B8_R1 |  | TCAGGCTGTGGGAACAAAT |
|  | TG_30_G7_F1 | FL640250 | GTACTTCACCACTTTATGTCTCTA |
|  | TG_30_G7_R1 |  | AACCCTGTATCGTCTGTCC |
|  | TG_310_A18_F1 | FL635688 | GCATCGACTTCGGGACTT |
|  | TG_310_A18_R1 |  | AGAGCTGGTGACGGGTATCT |
|  | TS_00_H9_F1 | FL641022 | CACGAGTGGTCCTCATTTC |
|  | TS_00_H9_R1 |  | CTTGTTGGTAAGCTCTTGTGATTT |
|  | TS_15_D6_F1 | FL642377 | TCCTGTCGATCACCCTCAT |
|  | TS_15_D6_R1 |  | TGGCAGCTATCATACCAACT |
|  | TS_23_C3_F1 | FL642833 | CTTTGGGTCCTGCCTTTA |
|  | TS_23_C3_R1 |  | TGAACTCTTTCAGCATTACGA |
|  | TS_24_A4_F1 | FL643430 | GATGCTCTGGTAGTACGGAA |
|  | TS_24_A4_R1 |  | CAGCAGTCGGAAGCTCAAT |
|  | TS_27_H9_F1 | FL643251 | CGCGGAACAGATTCAGGT |
|  | TS_27_H9_R1 |  | TCAGTTGTCTTGCTTCTAATCAAAG |
|  | TS_28_F12_F1 | FL643402 | CGCTGTTCTCGTGATTTCTG |
|  | TS_28_F12_R1 |  | GCTTCGTCTCCGGTTGTT |
|  | TS_33_D3_F1 | FL643896 | GCCTACGGACAAACCAAA |
|  | TS_33_D3_R1 |  | GCGAGTGCTTCATCTAACAA |
|  | TS_36_E2_F1 | FL644174 | CCAATGCAACACGATGTTCTA |
|  | TS_36_E2_R1 |  | ATGCTGGTCCATGTCCCTTT |
|  | TS_43_A11_F1 | FL644772 | AGCCACCAGAGGAAGGAA |
|  | TS_43_A11_R1 |  | TTTGTCGCAGAGCTAATGA |
|  | TS_43_B5_F1 | FL644787 | AACCTGCTGGGAGTCCTATT |
|  | TS_43_B5_R1 |  | CATCGCCACTCATCATTTCAT |
|  | TS_47_H3_F1 | FL645126 | GGGTATGATCGAACTCTTCTTG |
|  | TS_47_H3_R1 |  | TCCTCCGAACTTCTTTGGT |
|  | TS_48_A2_F1 | FL645137 | GCTTTGAAAGGCATTGTAGT |
|  | TS_48_A2_R1 |  | GGGTTTAGTTGACGGATAAC |
|  | TS_48_D11_F1 | FL645182 | ATGGCACTGATGGGAGACTTAC |
|  | TS_48_D11_R1 |  | GATCGGACTCAGATTCAGGAA |
|  | TS_52_A2_F1 | FL645325 | GCCAAGTTCCACCTCAATA |
|  | TS_52_A2_R1 |  | TCGATCTGTTCCAACATCA |
|  | TS_54_D1_F1 | FL645581 | GCTGATGACGTTGTTAATCTTG |
|  | TS_54_D1_R1 |  | CAGGTGCCATAAATGTGGA |
| Soldier head extract up-regulated | TG_305_M8_F1 | FL637031 | CCGGTCTGACACTTGTTATG |
|  | TG_305_M8_R1 |  | CGAAGTCGAAATCTGTGTTCT |
| Soldier head extract down- regulated | TS_42_D5_F1 | FL644436 | GAGCCCTTCTTGCTGTTT |
|  | TS_42_D5_R1 |  | CCTTCATCTCGTTCAATACTTTC |
|  | TS_43_E6_F1 | FL644818 | CCGCTGAAACAACGAGTAT |
|  | TS_43_E6_R1 |  | CAAATCGCGTCGAAATGA |
| Live reproductive up-regulated | TG_00_B5_F1 | FL635003 | TCGCAAGATGGAATGTAGTC |
|  | TG_00_B5_R1 |  | TCGGGTGTTATTGGGTAGT |
|  | TG_02_C3_F1 | FL637565 | TTGTCGTAACATCTCCATCTC |
|  | TG_02_C3_R1 |  | AAGCCGCCTCATTTCCAG |
|  | TG_305_F17_F1 | FL636990 | CAGCAAGCAGCAGGTAAAG |
|  | TG_305_F17_R1 |  | AGCTGTCGGAGCATGATGG |
|  | TS_29_D11_F1 | FL643545 | TGAGTTACGACGAGTTTCTA |
|  | TS_29_D11_R1 |  | GTCACCACAGTCCACCTAT |
| Live soldier up regulated | TG_03_E8_F1 | FL637681 | TCTGTCTGATCCTGGTTATTG |
|  | TG_03_E8_R1 |  | CTCGGTGCCTTCTTAGTTC |
|  | TG_07_D4_F1 | FL637865 | AGCCTCAGCCCGACAATC |
|  | TG_07_D4_R1 |  | GTTCAGAATCGACAGACAAGA |
|  | TG_09_A11_F1 | FL638301 | TGGACCCTTGTGAAATGAATG |
|  | TG_09_A11_R1 |  | CCAGTGTGGCAGTGTAGT |
|  | TG_15_G5_F1 | FL638734 | GCAGTCATTTCCACTCCAC |
|  | TG_15_G5_R1 |  | CCTCACGTTGTTTGCATCT |
|  | TG_23_E6_F1 | FL639382 | CGAGGAAACAACCCTATCAA |
|  | TG_23_E6_R1 |  | ACACCAAAGCATCTCCATCAG |
|  | TG_309_G17_F1 | FL635544 | ACGAAGAACTGGCGAGAATAG |
|  | TG_309_G17_R1 |  | ATGAGGCTTCACCCAAGGAG |
